# Acknowledgment to the Reviewers of *Materials* in 2022

**DOI:** 10.3390/ma16030987

**Published:** 2023-01-20

**Authors:** Materials Editorial Office

**Affiliations:** MDPI AG, St. Alban-Anlage 66, 4052 Basel, Switzerland

High-quality academic publishing is built on rigorous peer review. *Materials* was able to uphold its high standards for published papers due to the outstanding efforts of our reviewers. Thanks to the efforts of our reviewers in 2022, the median time to first decision was 15 days and the median time to publication was 38 days. Regardless of whether the articles they examined were ultimately published, the editors would like to express their appreciation and thank the following reviewers for the time and dedication that they have shown *Materials*:
A. B. M. Amrul KaishLucio MaragoniA. DurgaLucio Strazzabosco DornelesA. F. M. Anhar Uddin BhuiyanLucyna DymińskaA. HalimLudek StratilA. Hosseini MonazzahLudger WeberA. J. BabafemiLudi MiaoA. Muthu ManokarLudmila RudiA. Raja AnnamalaiLudovic CharpentierA. Rajesh KannanLudovica NucciA. S. S. SekarLudovica ParisiA. Sierra-FernandezLudovico MegaliniA. SkaropoulouLudwig CardonA. Yu. IvannikovLuigi Angelo CastriottaA.J. Sam SpearingLuigi CalabreseA.M. PereiraLuigi Carlo CapozziA.Yu. KonstantinovLuigi CoppolaAaditya KhanalLuigi De NardoAamer NazirLuigi Di BitontoAamir JalilLuigi Di SarnoAamod DesaiLuigi FortunaAaron FullerLuigi Mario ViespoliAaron S. Schwartz-DuvalLuigi NicolaisAaron Yu-Jen WuLuigi PigaAase ReyesLuigi SequinoAasif HelalLuis A. Román-RamírezAbasal HussainLuis Alberto AngurelAbay UsseinovLuis AlvesAbbas BahramiLuís BernardoAbbas HaddadiLuis CáceresAbbas J. Al TaieLuis Carlos Ardila TéllezAbbas ShafieeLuis CerdanAbdallah ElsayedLuís CoelhoAbdallah ShokryLuís CorreiaAbdalrhman MiladLuis Eduardo DíazAbdelaal AhmedLuis Felipe De Paula SantosAbdelaziz AboraiaLuis Felipe Verdeja GonzálezAbdelaziz ElgamouzLuis Gerardo ArriagaAbdelghani BenyoucefLuís Giner-TarridaAbdelghani IddarLuís Gonçalo Correia BaltazarAbdelhakim ElmouwahidiLuis Guerra RosaAbdelhakim MesloubLuis Jesús Villarreal-GómezAbdelhalim EbaidLuis LezamaAbdelhameed IbrahimLuis Miguel Moreno-RamírezAbdelhamid LayadiLuis OlmosAbdelilah AsserghineLuis Pallarés RubioAbdelkader AbderrahmaneLuis Picado-SantosAbdelkader AissatLuís PinhoAbdelkrem EltaggazLuís Pinto Da SilvaAbdellah El-hadj AbdellahLuis RosoAbdellatif Ibrahim SelmiLuís SousaAbdelmajid TimoumiLuis V. A. ScalviAbdelmalek Khorief NacereddineLuis Viana Da SilvaAbdelrahman ZkriaLuís VilhenaAbderrahim SamaoualiLuis Zamora PeredoAbderrahime SekkatLuisa BarbieriAbderrahmane AyadiLuisa Maria Gomez-SaineroAbderrazak BoutramineLuisa MedinaAbdessalem HajlaouiLuisa SpallinoAbdessamad DidiLuiz Antonio Alcântara PereiraAbdi AmirLuiz Antonio Ribeiro JuniorAbdollah AllahverdiLuiz Carlos SalayAbdollah BahadorLuiz Eduardo Dos Santos PaesAbdolmajid AlipourLuiz Fernando C. De OliveiraAbdolreza JamilianLuiz Fernando Romanholo FerreiraAbdolvahed KamiLuiz Henrique De AlmeidaAbdul Azeez Abdu AliyuLuiza BoninAbdul Hadi Bin AzmanLuka PavicAbdul Hakeem DeshmukhLukas BucinskyAbdul KhanLukas RycekAbdul MajeedLukáš StřižíkAbdul MohamedŁukasz Jacek SzparagaAbdul Muntaqim NajiŁukasz Jan LaskowskiAbdul Mutalib JaniŁukasz KonatAbdul Qadeer DayoŁukasz KucharskiAbdul RasheedŁukasz MajAbdul RohmanŁukasz NowakowskiAbdul Samad MahomedŁukasz PajchelAbdul ShaikŁukasz PałkaAbdul WadoodŁukasz ReimannAbdulallah ZeyadLukasz SadowskiAbdulaziz AlsaifŁukasz SadowskiAbdulaziz BubshaitŁukasz ŻyłkaAbdulaziz S. AlaboodiLuke MizziAbdulfatah Abdu YusufLuke NealAbdulgalim IsaevLuke ParryAbdülhakim ZeybekLuminiţa Maria NicaAbdullah Al MamunLuminita MarinAbdullah Al-HusseinLung-Chien ChenAbdullah AlmansourLuntao WangAbdullah Amru Indera LuthfiLupescu StefanAbdullah AslanLuqmanulhakim BaharudinAbdullah EkinciLusi ErnawatiAbdullah H. SofiyevLuyao ZhengAbdullah S. AlshammariLykourgos MagafasAbdullah TogayLyubomir LazovAbdullah Yahia AlFaifyLyubomir M. IvaskevichAbdulnaser M. AlshoaibiLyudmila M. LyalinaAbdulrahman AlwarthanLyudmila NyrkovaAbdulRazak AdnanLyudmila ParfenovaAbed Al Waheed HawilaM. Ali ButtAbeer SalahM. Alwan AlwanAbel VieiraM. Anthony XaviorAbhayraj S. JoshiM. Asun CanteraAbheetha PeirisM. BalasubramaniaAbhijit ChandraM. BalasubramanianAbhijit MajumdarM. Beatriz SilvaAbhijit SarkarM. D. KhomenkoAbhinav MalhotraM. Dolores La RubiaAbhisekh SahaM. Erden YildizdagAbhishek DasM. Federica De RiccardisAbhishek MishraM. Ijaz KhanAbhishek NagM. ManjaiahAbhishek Nitin DeshpandeM. Mansoor AhammedAbhishek SharmaM. NagamadhuAbhishek SrivastavaM. Pierre GibotAbhishek TevatiaM. R. M. AsyrafAbidhan BardhanM. Ramachandra BhatAbihijit KadamM. Raveendra KiranAbiodun Olagoke AdenijiM. S. BarakAbir De SarkarM. Sai Bhargava ReddyAbolfazl MohebbiM. SathishkumarAbraham FinnyM. Sezai DincerAbraham Loera MuroM. Shafiul AlamAbraham Vazquez-GuardadoM. Shaheer AkhtarAbrahams MwashaM. Siva Pratap ReddyAbraiz KhattakM. Vinod KumarAbrar BaluchM. Zubair IqbalAbu Bakar Mohd SupianM.Carmen Román-MartínezAbu Naser Md Ahsanul HaqueMabao LiuAbu Ul Hassan Sarwar RanaMabrouk SamiAbu Zafar Al MunsurMaciej BieniekAbudukarim SakaMaciej BikAbul Kalam AzadMaciej BojanowskiAca JovanovićMaciej ChotkowskiAcacio Rincon RomeroMaciej DobrzyńskiAchilleas ConstantinouMaciej HarasAchmad ZubaydiMaciej Koz̊uchAdalberto Benavides-MendozaMaciej ManeckiAdalgisa Rodrigues De AndradeMaciej MatysAdam CenianMaciej MotykaAdam ClancyMaciej NiedostatkiewiczAdam CwudzińskiMaciej PodgórskiAdam EkielskiMaciej PrzybyłekAdam GadomskiMaciej RadzieńskiAdam H. ClarkMaciej RoskoszÁdám HaimhofferMaciej SkotakAdam HamrouniMaciej SuligaAdam JakubasMaciej SydorAdam Khan (India)Maciej SzelągAdam Khan (Pakistan)Maciej ZubkoAdam KonefaŁMacmanus NdukwuAdam LipskiMadalena LiraAdam MasłońMadalina CiobanuAdam PiaseckiMădălina DumitriuÁdám VidaMadalina PandeleAdam WatrasMaddaka ReddeppaAdán Ramírez-LópezMaddalena MarchelliAdarsh Nayarassery NarayananMade SuciptaAdawiya J. HaiderMadeva NagaralAdebayo Olatunbosun SojobiMadhan JeyaramanAdedapo O. AdeolaMadhar A. HaddadAdel Al-GheethiMadhav ChavhanAdél LenMadindwa MashininiAdel Sadeghi‐NikMadis RatasseppAdela DumitrascuMadjid SoltaniAdelia AquinoMadlen UllmannAdhimar OliveiraMads BrandbygeAdib J. SaminMaeno TomoyoshiAdil AbdullahMafalda CostaAdil MarjaouiMagda MensiAdina SantanaMagdalena BarwiolekAditya Rio PrabowoMagdalena Bieda-NiemiecAdnan AbbasiMagdalena BłachnioAdnan MujezinovicMagdalena BrodaAdnan YounisMagdalena GierszewskaAdnane NoualMagdalena GizowskaAdolfo Di FioreMagdalena GłąbAdrian AntosikMagdalena GromadaAdrian AugustyniakMagdalena KopernikAdrian BurlacuMagdalena KrólAdrian Cǎtǎlin PuitelMagdalena LepickaAdrian ChlandaMagdalena Luty-BłochoAdrian CiutinaMagdalena M. MichelAdrian NeaguMagdalena Machno (Brazil)Adrian NicoaraMagdalena Machno (USA)Adrian RadońMagdalena MatuszakAdrián RojasMagdalena MichelAdriana BalanMagdalena Niemczewska-WójcikAdriana Burlea-SchiopoiuMagdalena OsialAdriana Eres-CastellanosMagdalena PaczkowskaAdriana EstokovaMagdalena RadovicAdriana Haydee Martínez RegueroMagdalena RogulskaAdriana M. CalvoMagdalena Skunik-NuckowskaAdriana UrdăMagdalena UrbaniakAdriano A. MendesMagdalena Valentina LunguAdriano BressaneMagdalena WróżyńskaAdriano Michael BernardinMagdalena Zawada-MichałowskaAdriano PiattelliMagdalena ZdanowiczAdriely GoesMagdalini D. TitirlaAfaq AhmadMagna GabrieleAfaque ManzoorMagnus J. AndersonAfonso AzevedoMagued IskanderAfonso Rangel Garcez De AzevedoMaha A. TonyAfrin HossaiMaha HassanAfshan BeyMahander Pratap SinghAfshin MaraniMahaveer KurkuriAgata BartyzelMahdi HasanzadehAgata JasikMahdi Niknam ShahrakAgata Lisinska-CzekajMahdi ZarifAgata M. NiewczasMahdieh AlipourAgata NiemczykMahdieh KhedmatiAgata PanethMahdieh SafyariAgata SawkaMahdieh Shahmardani FirouzjahAgata StempkowskaMahendra Kumar SamalAgata Szłapa-KulaMaher A. Al-BaghdadiAgata WawrzyńczakMahesh Ashok ShindeAgathe RobissonMahesh GanesapillaiAgathoklis A. KrimpenisMahesh KumarAggelos AvramopoulosMahesh Kumar JoshiAgnaldo M. FariasMahesh P. BhatAgnes CsiszárMahesh ParitAgnes PurwidyantriMahfooz SoomroAgni Raj KoiralaMahir UzunAgnieszka ChoroszuchoMahjoub JabliAgnieszka DrozdzikMahmood AhmadAgnieszka DudziakMahmood AliofkhazraeiAgnieszka DulskaMahmood Mastani JoybariAgnieszka Gadomska-GajadhurMahmood ShafieeAgnieszka JankowskaMahmood SharifitabarAgnieszka Kijo-KleczkowskaMahmoud AbadaAgnieszka KochmańskaMahmoud AbbasiAgnieszka KopiaMahmoud Abdel-HafiezAgnieszka KrólickaMahmoud Abo El-WafaAgnieszka LaskowskaMahmoud BakrAgnieszka LeszczyńskaMahmoud BaratiAgnieszka ŁukiewskaMahmoud EniebAgnieszka MarcinkowskaMahmoud Essam Abd El AzizAgnieszka NawrockaMahmoud Fawzy Mahmoud MoustafaAgnieszka SkoczylasMahmoud H. AkeedAgnieszka StolarczykMahmoud I. AbbasAgnieszka SzkliniarzMahmoud KhedrAgnieszka TercjakMahmoud ManiAgnieszka Teresa KrawczynskaMahmoud NasrAgnieszka TomczykMahmoud RasrasAgnieszka WoszukMahmoud Sayed (Egypt)Agostino MaurottoMahmoud Sayed (Japan)Agostino OcchiconeMahmoud Shaaban Sayed AhmedAgris BerzinsMahmoud Z. IbrahimAguinaldo FraddosioMahnaz NajafiAgusril SyamsirMahsa SalehiAgustin L. Herrera-MayMahyar KhorasaniAhad JavanmardiMahyar RamezaniAhamed IrshadMahzan JoharAhila Singaravel ChidambaranathanMaialen Espinal-ViguriAhlam NematiMainak K. GhoshAhmad AbdullahMaithilee MotlagAhmad AdewunmiMajda PavlinAhmad AminzadehMajed H. WakidAhmad Beng Hong KuehMajid A. MohammadiAhmad GolbabaiMajid Ali (Capital UniversiAhmad Hosseini-BandegharaeiMajid Ali (National UniversityAhmad Hussaini JagabaMajid BaniadamAhmad JamlehMajid GholhakiAhmad MajidMajid KhoramiAhmad MostafaMajid M.A. KadhimAhmad NourianMajid Movahedi RadAhmad Ostovari MoghaddamMajid Niaz AkhtarAhmad SadekMajid VahedAhmad SerjoueiMajid VaseghiAhmad T. AlmutawaMakino KotaroAhmad ZeeshanMakoto EndoAhmadipour MohsenMakoto TakiAhmar RaufMakram JafarAhmed A. GheniMaksim BoldinAhmed A. MohamedMaksim KrinitcynAhmed Abdelmottaleb OmarMakusu TsutsuiAhmed Abdelraheem FarghalyMalcolm GriffithsAhmed Abdulhamid MahmoudMałgorzata E. ZakrzewskaAhmed Al-JanabiMalgorzata HolynskaAhmed AlzamlyMalgorzata KacAhmed BakryMałgorzata KaczorowskaAhmed BelaadiMalgorzata KarolusAhmed DeifallaMałgorzata Musztyfaga-StaszukAhmed DorrahMałgorzata NorekAhmed Eid AlprolMałgorzata PająkAhmed ElbannaMałgorzata Perek-NowakAhmed ElbeihMałgorzata Szultka-MłyńskaAhmed El-FiqiMałgorzata SzymiczekAhmed ElkaseerMałgorzata Tatarczak-MichalewskaAhmed Elsayed AbouelregalMałgorzata WojtaszekAhmed F. FaheemMałgorzata Zienkiewicz-StrzałkaAhmed FatimiMałgorzata ZubielewiczAhmed FoulyMaligi Anantha SunilAhmed Fouzi TarchounMalik Adeel UmerAhmed H. MaamounMalik Muhammad NaumanAhmed I. A. Abd El-MageedMalika SaadaouiAhmed IbrahimMalin BrundinAhmed JalalMalinee SriariyanunAhmed Jamal Abdullah Al-GburiMalkeshkumar PatelAhmed KhaledMallarapu Gopi KrishnaAhmed L. AbdelhadyMalo RosemeierAhmed M. EbidMalvin N. JanalAhmed M. ElgarahyMamata SinghviAhmed M. TarabiaMamdouh EissaAhmed Ma A. El-SayedMamookho Elizabeth MakhathaAhmed MaamounMamoun FellahAhmed MehaneyMamzi AfrasiabiAhmed Mohammed Abu-Dief MohammedMan XuAhmed MouftiManab KunduAhmed N. AbdallaManabu KodamaAhmed Nuri KursunluManas Kumar MondalAhmed OmarManas Kumar SarkarAhmed S. DalaqManas Ranjan MajhiAhmed S. El-ShafieMandeep Singh Jit SinghAhmed S. OudaManesj YewaleAhmed Saad RashedMangesh MadurwarAhmed Salih MohammedMangilal ChoudharyAhmed SenouciMani DhananchezianAhmed Serag FariedManić NebojšaAhmed ShakerManickam MinakshiAhmed TahwiaManickam RameshAhmed Tareq NoamanManickam RavichandranAhmed TawfikManieniyan V. ManieniyanAhmed ThabetManika J.N.V. PrasadAhmed Yaseen AlqutaibiManikandan NarendranAhmed Yehia ShashManikanta Ravindra Kumar VakkalagaddaAhmet CanManinder SinghAhmet GuralManish KumarAhmet Hakan YilmazManish PandeyAhmet Hayrullah SevinçManjeet SinghAhmet YildizManjunath MaddikeariAida RahmaniManjunath Patel GowdruAidin Bordbar-KhiabaniManjunath ShettarAikaterini Ioannis VavourakiManjusha BattabyalAikaterini RogkalaManlio SantilliAike QiaoMannix P. BalanayAimée Maria GuiottiManny MinakshiAimin ChangManohar GuttikondaAires CamõesManoharan ManikandanAirton DeppmanManoli M. ZubiturAirton Germano Bispo-JrMansoo JounAissa HarhiraMansoor AlshehriAissam AiroudjMansoor BozorgAit Addi AbdelazizManuel AngstAitor C. RaposeirasManuel AurelianoAivars ĀboltiņšManuel CarpioAjay AnnamareddyManuel De Jesús Castro-RománAjay D. PingaleManuel EvaristoAjay Kumar ChinnamManuel Filipe Pereira Cunha Martins CostaAjay Vikram SinghManuel Francisco Costa PereiraAjaya Kumar SinghManuel H. De La Torre IbarraAjit BeheraManuel J. Lis AriasAjitanshu VedrtnamManuel Jesús Hermoso-OrzáezAkbar Afaghi KhatibiManuel JordánAkbar AlibeiglooManuel José FreitasAkbar HeidarzadehManuel Marques FerreiraAkbar HojattiManuel MelendrezAkhil KhajuriaManuel PiñeroAkhil TayalManuel ToscanoAkif KutluManuel Uceda-RodríguezAkihiko FujiwaraManuel V. RamalloAkihiro HachikuboManuela CurcioAkikazu ShinyaManuela FerraraAkil AhmadManuela ListAkinobu UsamiManuela Moreira Da SilvaAkira MurakamiManvendra VermaAkm S. RahmanManzoore Elahi M. SoudagarAko DaraeiMao-Hua ZhangAkram Hosseinian SerajehlooMaqsood MalikAkshay ModiMara TerziniAkshay MoudgilMarat EseevAkter HosenMarat KhayrutdinovAlaa Al-RkabyMarc Calvo-SchwarzwälderAlaa El Din MahmoudMarc GenestAlaa FahmyMarcel F. KunrathAlaa Yassin Al-AhmadMarcel FedákAlaeddin Burak IrezMarcel KunrathAlain MoissetteMarcela E. TrybulaAlam ShahMarcela Elisabeta Barbinta PatrascuAlan CarterMarcela PekarcikovaAlan HellierMarcela PokusováAlar JustMarcello BertoAlawi SulaimanMarcello IasielloAlbena PaskalevaMarcelo AssisAlbert E. ParkerMarcelo Bender PerotoniAlbert E. PattersonMarcelo E.H. Maia Da CostaAlbert Estrugo-DevesaMarcelo MaruchoAlbert ManeroMarcelo RibeiroAlbert PattersonMarcelo Yudi IcimotoAlbert PoaterMarcin BanachAlbert SerràMarcin BarburskiAlbert Thomas AnastasioMarcin BasiagaAlbert Wen Jeng HsueMarcin ChutkowskiAlberto AlfanoMarcin ChybińskiAlberto BaldelliMarcin D. SyczewskiAlberto BenatoMarcin GnybaAlberto DebernardiMarcin GodzierzAlberto FraileMarcin GołąbczakAlberto García-PeñasMarcin GórnyAlberto GregoriMarcin GrabaAlberto Herrera-GómezMarcin JesionekAlberto MonsalveMarcin K. WidomskiAlberto MuscioMarcin KnapińskiAlberto PardoMarcin KoniorczykAlberto Rodríguez-ArchillaMarcin KremieniewskiAlcineia OliveiraMarcin LemanowiczAldo GhisiMarcin LiberaAldobenedetto ZottiMarcin LosAldona KrawczykowskaMarcin ŁośAlejandro CornejoMarcin MadejAlejandro DuránMarcin MaździarzAlejandro González-PociñoMarcin NabiałekAlejandro Manzano-RamírezMarcin SajdakAlejandro May-PatMarcin ŚrodaAlejandro Pérez LariosMarcin StaszukAlejandro RodriguezMarcin StawarzAlejandro YawnyMarcin SzczechAleksandar ĆirićMarcin SzymańskiAleksandar KondinskiMarcin WekwejtAleksandar LolićMarcin WinnickiAleksandar MatkovićMarcin WłochAleksandar SedmakMarcin WojtyniakAleksander HejnaMárcio RodriguesAleksander Yu DolgoborodovMárcio Zaffalon CasatiAleksandr G. KasikovMarco Actis GrandeAleksandr GokhmanMarco AnniAleksandr KlimovMarco Arturo García-RenteríaAleksandr OreshonkovMarco BonoperaAleksandr PishtshevMarco CaniatoAleksandr S. KlimovMarco CapogniAleksandr S. PozdnyakovMarco ChiariniAleksandr SakhnevychMarco CiveraAleksandr VedernikovMarco ColellaAleksandr VolkovMarco CorradiAleksandra DeptuchMarco DolciAleksandra Grząbka-ZasadzińskaMarco FerrariAleksandra JarońMarco Filippo FerrottoAleksandra KozłowskaMarco GandolfiAleksandra KrampikowskaMarco GirolamiAleksandra Kuryłowicz-CudowskaMarco LambertiAleksandra MałachowskaMarco Lamperti TornaghiAleksandra MarciniakMarco LezzeriniAleksandra MišanMarco ManciniAleksandra Smejda KrzewickaMarco MenegozzoAleksandra WdowczykMarco MilanesioAleksandrs PlatonenkoMarco MiliucciAleksei ObrosovMarco MontemurroAleksei V. AlmaevMarco NaiaAleksej ZarkovMarco PeresAleksey A. VedyaginMarco PizzorniAleksey AbrashovMarco PortelliAleksey KuznetsovMarco Roberto CavallariAleksey M. VishnyakovMarco Sanna AngotziAleksey NokhrinMarco ScaviniAleksey V. ZaitsevMarco SchowalterAleksey YaremchenkoMarco SeracchianiAlemayehu Nana KoyaMarco SimoncelliAlena OčkajováMarco TallaricoAlena Opalkova SiskovaMarco TanganelliAlena PietrikovaMarco TatulloAlena PribulováMarco TogniAlena VimmrováMarco TruccatoAlena ZemanováMarco ZannottiAleš FidlerMarcondes Lima Da CostaAles MizeraMarcos A. L. Dos ReisAleš NagodeMarcos Augusto De Lima NobreAleš StražeMarcos BatistellaAlessandra AmatoMarcos De Oliveira JuniorAlessandra LuccheseMarcos DiazAlessandra SorienteMarcos Flavio De CamposAlessandra ZizzariMarcos GutiérrezAlessandro AntonelliMarcos I. FloresAlessandro CarrozzaMarcos Rodríguez MillánAlessandro CavalloMarcus DymondAlessandro D’EliaMarcus KleinAlessandro Dell’EraMarcus StoetzerAlessandro Di MarcoMarcus Vinícius Tavares Da CostaAlessandro FaisMarcus YioAlessandro GalendaMarek BarskiAlessandro MarradiMarek BouskaAlessandro VeltriMarek ChaleckiAlessandro ZonaMarek GancarzAlessia ArtesaniMarek GaworskiAlessia Di VitoMarek GlińskiAlessia FilipponeMarek GuziewiczAlessia GiordanaMarek IwańskiAlessia Serena PernaMarek KlimczakAlessio AndreoniMarek KowalikAlessio CascardiMarek KrólikowskiAlessio LampertiMarek LiederAlessio MezziMarek MajAlessio VallettaMarek OchowiakAlessio VitaMarek PokornyAlessio ZulianiMarek PolanskiAlex Bruno Lobato RodriguesMarek PszczolaAlex De OliveiraMarek PszczołaAlex Elías-ZúñigaMarek SzkodoAlex KuchumovMarek SzostakAlex Palma-CandoMarek TulejAlexander A. GromovMarek UrbanskiAlexander A. KuznetsovMarek VojtkoAlexander A. MinakovMarek Vrabel’Alexander Alexandrovich GromovMarek WasiucionekAlexander AroninMarek WesołowskiAlexander AxelevitchMarek WieruszewskiAlexander B. StraumalMarek WisniewskiAlexander BakerMarek WojtaszekAlexander BerezinMarek WróbelAlexander BerrocalMargaret GradovaAlexander BungeMargarida M. J. QuinaAlexander ChausMargarita A. KhimichAlexander ChenMargarita GoldbergAlexander DegtyarevMargarita GrozevaAlexander E. MayerMargarita Tecpoyotl-TorresAlexander EliseevMargherita IzziAlexander FominMargret Sibylle EngelAlexander G. KolpakovMari Ángeles TenaAlexander GagarinMari KoikeAlexander GoncharovMaria A. ChiacchioAlexander GramlichMaria AguilarAlexander IvannikovMaria Asunción IllarramendiAlexander KalinkinMária BehúlováAlexander KomissarovMaria BrzhezinskayaAlexander KonevMaria ButakovaAlexander KromkaMaria ChernyaevaAlexander M. KorsunskyMaria CinefraAlexander MuravskyMaria CortazarAlexander P. PyatakovMaría CriadoAlexander PestovMaria Cristina Garcia-AlonsoAlexander PogrebnjakMaria Cristina VilaAlexander PonomarevMaría del Mar AlonsoAlexander RodinMaría Del Refugio Castañeda-ChávezAlexander RybakMaria DemeterAlexander S. AntonovMaría Dolores Fernández RamosAlexander S. BrandMaría Dolores Gómez PulidoAlexander S. FominMaria Dolores Rubio-CintasAlexander S. KrylovMaría Elena Sánchez-VergaraAlexander SafonovMaria Elisa TataAlexander SamokhvalovMaria Elisabete C.D. Real OliveiraAlexander SavinMaria Erminia GenoveseAlexander ShcherbakovMaria Evarista Arellano-GarciaAlexander ShkarovskiyMaria Francesca Di FilippoAlexander SmirnovMaria GazdaAlexander SobolevMaria Giovanna FrancipaneAlexander TarasovMaria GolubevaAlexander TsouknidasMaria GomezAlexander V. Artem’evMaria Graciela AguayoAlexander V. GlushkovMaria Grazia OrtoreAlexander VelmuzhovMaria GreabuAlexander VtyurinMaria HarjaAlexander WelleMaria Helena BragaAlexander Yu ChuryumovMaria Helena CasimiroAlexander ZherebkerMaria Helena FigueiralAlexander СherpakovMaria Icíar Arteagoitia CalvoAlexandr A. LyapinMaría Isabel Lamas GaldoAlexandr KazachenkoMaria J HerreroAlexandr KolesnikovMaria KalivaAlexandr SadovnikovMaria KonstantakiAlexandr ShchegolkovMaría Laura VeraAlexandra KuriganovaMaria LazaridouAlexandra MocanuMaria Leilani TorresAlexandra NistorMaria LeporeAlexandra VinagreMaria Lia NapoliAlexandre Da Costa SenaMaria Luísa Braga FarinhaAlexandre GiacobboMaria Luisa GrilliAlexandre Luiz Amarante MesquitaMaria Luminita ScutaruAlexandre M. P. BotasMaria LundgrenAlexandre N. MagasinskiMaria Martín-MoralesAlexandre PierreMaría MeloAlexandre Queiroz BracarenseMaría Natividad AntónAlexandre ZakharovMaria NetoAlexandrina MunteanMaria NikolicAlexandros K. NikolaidisMaria NikolovaAlexandru Mihai GrumezescuMaria Pia FerrazAlexei VinogradovMaria PomoniAlexey A. SerdobintsevMária PorubskáAlexey A. VereshchakaMaria PrincipeAlexey BeskopylnyMaria RâpăAlexey G. KolmakovMaria RaposoAlexey GorevoyMaria Rita CicconiAlexey KomolovMaría Rizo-GorritaAlexey N. FedorenkoMaria Rosaria AcocellaAlexey ProsviryakovMaria Rosaria MasulloAlexey RodinMaria RybarczykAlexey RomanovMaria SarapultsevaAlexey S. KubasovMaria SokolikovaAlexey S. LipatievMaria StarowiczAlexey SalimonMaria StefanidouAlexey ShutovMaria SvedaAlexey SmolinMaria TsoutsouvaAlexey TarasovMaria Victoria CabañasAlexey VereschakaMaria VikulovaAlexey YapryntsevMaria WiesławaAlexios PapacharalampopoulosMaria ZieleckaAlfian NoviyantoMaria-Carmen AsensioAlfonsas DaniūnasMariachiara TrapaniAlfonso EscamillaMaria-Dolores AvilésAlfonso Fernández-CanteliMariagrazia FortinoAlfonso MartoneMarialucia GalloriniAlfredo AguadoMarialuigia RaimondoAlfredo Flores ValdésMariamelia StanzioneAlfredo GomesMarian DrienovskyAlfredo IandoloMarian Gabriel BanciuAlfredo MoralesMarian GrigorasAlfredo SuárezMarian IonAlfrendo SatyanagaMarián KučeraAli Abdulhussein ShubbarMarian PiwowarskiAli AhmadMarián ReiffersAli AlghamdiMariana Altenhofen Da SilvaAli AlkhataniMariana Amorim FragaAli AlwahibMariana Domnica StanciuAli ArabMariana PacurarAli ArmanMariana SouzaAli BahadurMarianela RipaniAli CherifMariangela LonghiAli DadMariangela QuartoAli DalalbashiMariann HarangiAli DavarMarianna GniadekAli Dehghan FiroozabadiMarianna Marciszko-WiąckowskaAli Farokhi NejadMarianna RinaldiAli GhamariMarianne PrévôtAli GünenMariano Fernandez FairénAli H.A. Al-WaeliMariano RomeroAli JabbariMariateresa LettieriAli JohariMarie A. CornelisAli KasalMarie-Christine RecordAli KhalfallahMarie‐Laure JourdainAli LashkariMarie-Pierre SantoniAli M. AbdelhafeezMarija IvanovicAli Mohamed Kamal AhmedMarija KrsticAli Mohammed BashaMarija M. VukčevićAli Osman AtahanMarija M. VuksanovićAli PaknahadMarija MilanovićAli PassianMarija MojicevicAli RazaMarija StojmenovićAli Reza RezvaniMarijana Hadzima-NyarkoAli Reza ZanjanijamMarijana PonjavicAli SadeghianmaryanMarijo BuzukAli SahafMarilou Cadatal-RadubanAli SalehizadehMarimuthu BhuvaneswariAli SalemMarin MicutzAli ShabaniMarin UgrinaAli ShehadehMarina Alekseevna FominaAli ShokriMarina AmaralAli ShubbarMarina AndreevaAli SoltaniMarina FialloAli TafarojnoruzMarina G. ShelyapinaAli TalimianMarina GravitAli TasallotiMarina I. KozhukhovaAli ZaidiMarina Ilakovac KvederAli ZarrabiMarina KirillovaAli. J. ChamkhaMarina KrjuchkovaAliakbar AshkarranMarina ShavelkinaAliakbar EmdadiMarina V. BaidakovaAliaksandr A. KasachMarina V. Il’inaAliaksandr ShaulaMarina VukojeAlice BerardoMarina Yu. StogniyAlice HospodkováMarina ZoccolaAlicja Krystyna KrellaMarinela I. PanayotovaAliev Firdavs AbdusamievichMarino BrčićAlin DinitaMario Alberto García RamírezAlina Adriana MineaMario BeinerAlina Bianca PopMario CaggianoAlina Ioana BadanoiuMario GutiérrezAlina KarabchevskyMario InclánAlina Marinela IonescuMário João S. F. SantosAlina MateiMario ProsaAlina RaditoiuMário R. VasconcelosAlina VladescuMario RaspantiAlireza Akhavan-SafarMario Rui ArrudaAlireza Bagher ShemiraniMario ŠokacAlireza EnshaeianMário Vanoli ScatolinoAlireza FarhadizadehMarique AucampAlireza FarzampourMaristella E. VoutetakiAlireza GhanizadehMarius GhejuAlireza HadiMarius PrelipceanuAlireza JavadianMariusz BoberAlireza KarimiMariusz DąbrowskiAlireza ModirMariusz DejaAlireza MoradkhaniMariusz FabijańskiAlireza Nezamzadeh-EjhiehMariusz GackowskiAlireza OstadrahimiMariusz HasiakAlireza Reza FarrokhiMariusz HoltzerAlisson Mendes RodriguesMariusz JaczewskiAljoša ŠajnaMariusz JaśniokAlka GuptaMariusz KamińskiAlma ValorMariusz KosobudzkiAlmaz SaifutdinovMariusz KrupińskiAlok Kumar DasMariusz MajchrzakAlokesh PramanikMariusz MrózekAlpay Tamer ErtürkMariusz TryznowskiAlperen DegirmenciMariusz WalczakAlrowaili Ziyad A.Mariusz WinieckiAltan KayranMariusz ZoltowskiAltino LoureiroMariya AleksandrovaAlvaro Della BonaMariya SpasovaÁlvaro GonzálezMariya V. EdelevaAlvaro González-AngelesMarjan SalariÁlvaro González-GarcinuñoMarjan TusarÁlvaro Martínez-CamarenaMárk AntalAlvaro RidruejoMark ArnouldÁlvaro Silva RibeiroMark B. FramptonAlvaro Y. TesioMárk FráterÁlvaro Zubizarreta-MachoMark GolitkoAmador Menéndez-VelázquezMark James JacksonAmahyar KhorasaniMarkel VinogradovAmaia Soto BeobideMarkku LeskeläAmal NassarMarko JakovacAmalraj MarshalMarko NagodeAmanda C. MillsMarko OreškovićAmanda Maria De Oliveira Dal PivaMarkos PetousisAmani DiremMarkus GahleitnerAmany Mohammed FekryMarkus HeßAmarajothi DhakshinamoorthyMarkus OeserAmardeep SinghMarkus PristovsekAmarendra N. MisraMarkus RatzkeAmbra GuarnaccioMarkus SchleeAmbrina A QureshiMarlene Andrade GuelAmbrish SinghMarlous KampAmerica CalifanoMaros MartinkovicAmérico Bortolazzo CorrerMarta Álvarez-LealAmerico ScottiMarta BarbaAmerigo GiudiceMarta C.P.C. CorvoAmey KhanolkarMarta CaroselliAmilton BarbosaMarta Dyszkiewicz-KonwińskaAmin AbbasiMarta FerociAmin Al-FakihMarta FerraroAmin Amiri DeloueiMarta GrochowiczAmin Babaei-GhazviniMarta KadelaAmin BahramiMarta KałużaAmin Hamed MashhadzadehMarta M. MarínAmin HekmatnejadMarta MikuśkiewiczAmin KhajehdezfulyMarta OrlowskaAmin MojiriMarta Prześniak-WelencAmin NozariasbmarzMarta Roig FloresAmin RabieiMarta SerranoAmin ShavandiMarta SkafAmine BendarmaMarta SłowikAmir AfzalMarta WaloAmir Ebrahimi-MoghadamMartha Guerrero-MataAmir ElzwawyMarthias SilwambaAmir Ershad-LangroudiMartin BojinovAmir H. MosaviMartin CvekAmir Hossein BaghdadiMartin DaňoAmir M. YousefiMartin DeckyAmir Mahyar KhorasaniMartin DoškářAmir MellatiMartin Ek RosénAmir MofidiMartin FiskAmir Muhammad AfzalMartin FriákAmir NajibiMartin FrießAmir NavidfarMartin FujdaAmir NouraniMartin HeilmaierAmir RostamiMartín Herrera-TrejoAmir SafieyMartin HušekAmir TabakovićMartin KeppertAmir WaseemMartin Lopez-GarciaAmir ZadaMartin MelicharAmirhosein ShabaniMartin MichálekAmirhossein HasaniMartin MorelliAmirhossein MadadiMartín Ortiz-DomínguezAmiri Taher YousefiMartin Ostoja-StarzewskiAmirjalal JalaliMartin PetrAmirkianoosh KianiMartin PetrunAmirreza KhodadadianMartin SahulAmirreza MasoodiMartin SedlmajerAmit BansalMartin SharpAmit GoyalMartin SteinbrückAmit Kumar VermaMartin StěničkaAmit MahajanMartin StockingerAmit Md Estiaque ArefinMartin TsvetkovAmit ThakurMartin VeselýAmitav TikadarMartin VögeleAmitava MoulickMartin ZelenyAmjad AlbayatiMartina GerkenAmjad El-QanniMartina LaubertovaAmjad HussainMartina RomaniAmm Nazmul AhsanMartina VračevićAmmar Abdulrahman JairounMartis GeorgianaAmmar PatelMárton SzabadosAmna SafdarMarwa AliAmna SiddiqueMarwa ElkadyAmnon ShirizlyMarwan EffendyAmol V. PansareMarwan El MobadderAmparo Verdú-VázquezMarwan KhraishehAmr A. NassrMary BatistaAmr ElnemrMary Beth Browning MonroeAmr FoudaMary Lyn C. LimAmr KaoodMaryam AleemAmrit Kumar ThakurMaryam KhosraviAmrit ŠorliMaryam ShaterianAmrita BhattacharyaMaryam YazdanmehrAmrita KunduMarzanna KsiazekAmro El BadawyMarzena KurpinskaAna AlmeidaMarzena LachowiczAna ArauzoMarzena Mitoraj-KrólikowskaAna Beatriz Morales CepedaMarzena PawlikAna C. PinhoMarzio GrassoAna Catarina DuarteMasafumi UnnoAna Catarina PinheiroMasahide HaradaAna Catarina SousaMasahiko YamaguchiAna CazacuMasahiro KusanoAna Cristina FreireMasahiro SakataAna De SousaMasaki MichihataAna DuarteMasakuni YamaguchiAna Filipa PiresMasamichi AkazawaAna Gómez-VarelaMasanobu MiyataAna Isabel Gracia LostaoMasanori HaraAna Laura Luna BarronMasanori KohnoAna Luísa RodriguesMasanori YamamotoAna M. BeltranMasao IrieAna Margarida PereiraMasao YoshinoAna Maria Dos Santos Rosa Da CostaMasaomi IkedaAna Maria Julieta PopescuMasaru AniyaAna Maria MendezMasashi IkegamiAna Maria PiresMasashi SekineAna Maria PutzMasashi WatanabeAna Marta De MatosMasataka KuboAna Morales-RodríguezMasato TanakaAna Paula Betencourt Martins AmaroMasatoshi FutakawaAna Paula MacedoMasaya IchimuraAna Paula PiedadeMasayuki OkugawaAna Paula Serafini ImmichMasayuki ShimojoAna PavlovicMasayuki YamaguchiAna Pilar Valerga PuertaMasfer AlkahtaniAna PilipovicMasfueh RazaliAna PimentelMasood S. AlivandAna SilvaMasoud AhmadiAna Teresa Corte-RealMasoud MoshtaghiAna TomićMasoud Nazarian-SamaniAna Virginia SocaliciMasoud Rais-RohaniAnagh BhaumikMasoud SabziAna-Maria IordacheMasoumeh GhalkhaniAnamarija FarkašMasoumeh OzmaianAnand ChokkalingamMasrina Binti Mohd NadzirAnand GauravMassimiliano AvalleAnand Kishore KolaMassimo CarossaAnand N.Massimo CorsaliniAnand YadavMassimo LorussoAnandram VenkatasubramanianMassimo MarielloAnantharamaiah P. N.Massimo MarzinottoAnanya BaksiMassimo NocenteAnastasia MikhaylovskayaMassimo VivianiAnastasia NazarovaMastoura Mohamed EdreesAnastasia PournaraMasumi InoueAnastasiadis SpirosMateescu CarmenAnastasios GotziasMateus Garcia RochaAnastasiya TönjesMateusz BarczewskiAnastassiya A. MashentsevaMateusz GierszewskiAnat RuangrassameeMateusz IwańskiAnatoli PopovMateusz JackowskiAnatolii DarinskiiMateusz JaniszewskiAnatoliy KovalevMateusz KopecAnatoliy RinkevichMateusz KopećAnatoly A. RogovoyMateusz KowalikAnatoly Dvurechenskii Mateusz KowalskiAnatoly I. ProstomolotovMateusz KudasikAnatoly MitrofanovMateusz KuklaAnatoly ResnyanskyMateusz MrukiewiczAnawati AnawatiMateusz PizAnca CojocaruMateusz WyrzykowskiAnca DumbravaMatheus PolettoAnca Maria JuncanMathieu DomenjoudAnca MazareMathilde ChampeauAnca Niculina CadinoiuMathilde MonachonAnchalee ManonukulMatic Jovičević-KlugAnders HenningsenMatija JezeršekAnders JohanssonMatija ZlatarAnderson Joel SchwankeMatilda ZemanováAnderson PukasiewiczMatjaž KristlAndi TrimulyonoMatjaz SkrinarAndjela DraganićMatlab N. MirzayevAndjelika BjelajacMatteo AlberghiniAndras Jozsef TothMatteo Bruno LodiAndrâs RoószMatteo CirilloAndre CastroMatteo MastelloneAndré ChristoforoMatteo SambucciAndre ContinMatteo ZanoccoAndré FurtadoMatthew CavalliAndré L. BogadoMatthew MarkoAndré L. Menezes De OliveiraMatthew Matthew N. CavalliAndré MartinMatthew O’ReillyAndré P. TschiptschinMatthew PanAndré PereiraMatthew SwensonAndré RangelMatthew WadgeAndré Rolim BabyMatthias EiflerAndré SchulzMatthias J. RoggendorfAndre StrydomMatthias KarlAndré Ulisses Dantas BatistaMatthias KindAndrea Anna SowislokMatthias KroegerAndrea Antonino ScamporrinoMatthias NeumannAndrea AtreiMatthias RoedigerAndrea BalielloMatthias WangenheimAndrea BernasconiMatthias ZehnderAndrea BraccialiMatthieu MangeatAndrea CafarelliMattia BartoliAndrea Di SchinoMattia FrascioAndrea EhrmannMattias ThuvanderAndrea Francesco CiuffiniMatúš KováčAndrea GattoMatúš MolčanAndrea GrazianiMauricio A. Sarabia-VallejosAndrea LabouriauMauricio LlaverAndrea LazoMauricio RodriguezAndrea LongobardoMaurizio AngeloneAndrea Mariano SiliMaurizio ArenaAndrea MescolaMaurizio CasarinAndrea MichelettiMaurizio FerrariAndrea MontaninoMaurizio ForteAndrea Navarro-QuezadaMaurizio OrlandoAndrea PastoreMaurizio PascadopoliAndrea PetrellaMauro BassettiAndrea PiccolroazMauro ChinappiAndrea PrannoMauro CoduriAndrea RónaváriMauro D’ArcoAndrea ScribanteMauro MagnoniAndrea SerafiniMauro Mitsuuchi TashimaAndrea SiliMauro ZarrelliAndrea SimonMax MarianAndrea SütőováMaxim A. TyulenevAndrea TerenziMaxim DvornikAndrea ToffoliMaxim GorkunovAndrea ZanichelliMaxim KhomutovAndrea ZilleMaxim M. KorshunovAndreas EndruweitMaxim OzerovAndreas HoffmannMaxim ShandrikovAndreas KlingMaxim ZdorovetsAndreas KulovitsMaxime BernierAndreas LeineweberMaxmilian SokolukAndreas RiesMayadhar DebataAndreas RosenkranzMayank ChaturvediAndreas SapalidisMayank GuptaAndreea CostasMayank MishraAndrei BogatovMayank PatelAndrei DontuMayara AmarioAndrei EgorinMayara Sarisariyama Siverio LimaAndrei IvanetsMaykel ManawanAndrei KlyndyukMayra Polett GurrolaAndrei KosterovMazen AlshaaerAndrei KrasilinMaziar DehghanAndrei RineiskiMd Abdul Kader ZilaniAndrei ShishkinMd Akhtar HossainAndrei TurutinMd ArifuzzamanAndrei V. KovalevskyMd Azree Othuman MydinAndrei V. MostovshchikovMd Jihad MiahAndrei V. ShevelkovMd Mahbubur RahmanAndrei Vasile NastutaMd Milon HossainAndrei Victor SanduMd Mofasser MallickAndreia RuivoMd Mottahir AlamAndrej ThurzoMd Najib AlamAndrés Alonso Agudelo-SuárezMd Rashadul IslamAndres Antonio Torres AcostaMd Reza E. RabbyAndrew BallantyneMd Saiful IslamAndrew ChuangMd Shahariar ChowdhuryAndrew GrygućMd. AkhtaruzzamanAndrew I. M. GreerMd. Akter HosenAndrew J. GrossMd. Ashraful Islam MollaAndrew J. SlifkaMd. Irfanul Haque SiddiquiAndrew N. HrymakMd. Nahid PervezAndrew PaluchMd. Nizam UddinAndrey A. DubrovskiyMd. Reazuddin ReponAndrey A. KirsankinMd. Sofiqul IslamAndrey A. SamokhvalovMeda-Lavinia NegrutiuAndrey B. YaroslavtsevMedard MakrenekAndrey BelyakovMeei Mei GuiAndrey Budimirovich PonomarevMegat Ahmad Kamal Megat HanafiahAndrey E. KrauklisMegat HanafiahAndrey FilippovMegawati ZunitaAndrey G. MochugovskiyMeghanshu VashistaAndrey GatinMeghna SharmaAndrey I. BazlovMehdi DerradjiAndrey KrauklisMehdi Ebadi JamkhanehAndrey MarkinMehdi Hatefi-ArdakaniAndrey MaximenkoMehdi KhojastehpourAndrey MazilkinMehdi KiaAndrey MedvedevMehdi MehraliAndrey MereshchenkoMehdi MoayyedianAndrey NovitskyMehdi PanjiAndrey O. DoroshenkoMehdi PourabdoliAndrey O. ZhigachevMehdi SafariAndrey OsipovMehdi ShahrakiAndrey P. ZhdanovMehdi Shakourian-FardAndrey SamokhinMehdi SolaimaniAndrey SarikovMehdi Torabi-KavehAndrey SidorenkoMehmet Akif ErdenAndrey SolovyevMehmet Ali GülgünAndrey SuzdaltsevMehmet AvcarAndrey V. BondarevMehmet Erdi KorkmazAndrey V. EmelyanovMehmet Ergün HatirAndrey V. FilippovMehmet EsenAndrey VorontsovMehmet GürsoyAndrey ZubarevMehmet KılıçAndri KusbiantoroMehmet KodalAndriana SurlevaMehmet Onur FenAndrii G. KostryzhevMehmet PalanciAndrii KostyniukMehmet SenelAndrii KoveriaMehmet YilmazAndrii MurdzaMehrab NodehiAndrii VelychkovychMehran KhanAndriy BanduraMehrdad MesgarpourAndriy BurbelkoMehrdad NikravechAndriy KvashaMehrous AliAndryushin Konstantin PetrovichMehtap OzdemirAndrzej AmbroziakMehtap SahinerAndrzej BiessikirskiMeiyong LiaoAndrzej BorawskiMelanie TimpelAndrzej BrudnickiMelinda KrebszAndrzej DzierwaMelis OlcumAndrzej GrabowskiMelkie Getnet TadesseAndrzej Jarosław PanasMeltem YanilmazAndrzej KatuninMelvin DiazAndrzej KiełbusMemduh KaralarAndrzej KochańskiMeng Fang LinAndrzej KomorekMeng GaoAndrzej KozłowskiMeng JiangAndrzej KrukMeng WuAndrzej KubitMengfei YuAndrzej KulczyckiMeng-Hsiu TsaiAndrzej MamalaMenglim HoyAndrzej MiklaszewskiMengqiang ZhaoAndrzej MiszczykMengtao SunAndrzej MolakMengye WangAndrzej PerecMengyu ChaiAndrzej PolańczykMeng-Yun ChungAndrzej PtokMerajuddin KhanAndrzej PuszkaMeram AbdelrahmanAndrzej ŚlebarskiMerce PaciosAndrzej StefanikMert GuneyAndrzej SzajekMert VuralAndrzej TrytekMeshal AlzaidAndrzej UbyszMetin ÇalışırAndrzej ŻakMeysam BayatAndrzej ZielinskiMeysam KeshavarzAndy Wai Kan YeungMian Sohail AkramAnele MpupaMiaoyan CaoAneta BartkowskaMiće JakićAneta HerbutMichael AdabreAneta JakubusMichael ArkasAneta PetelskaMichael ArulAneta SlodekMichael BartonAnette MüllerMichael BererAng ZhangMichael BertocchiAngel AnchevMichael DaubÁngel De La Rosa VelascoMichael FischlschweigerAngel Miguel Pitarch RoigMichael FleckAngel MoralesMichael GoepelAngel MurcianoMichael HornAngel PeralMichael I. OjovanAngela BarredaMichael JaffeAngela De BonisMichael KnitschkeÁngela I. López-LorenteMichael KovalevAngela IvaskMichael LeitnerAngela Jedlovszky-HajduMichael M. SlepchenkovAngela KinoshitaMichael MangoldAngela LongoMichael MaukschAngela NigroMichael MayAngela SpoialăMichael Oluwatosin BodunrinAngeliki PapalouMichael PissasAngelo PutignanoMichael R. KoblischkaAngelo Savio CalabreseMichael RegevAngelo TroedhanMichael RethmeierAngelos P. MarkopoulosMichael Rivera MananghayaAngga HermawanMichael RohlfingAnghel CernescuMichael Rudolf KoblischkaAnh HuynhMichael SchefflerAnh TranMichael SchmidtAnh Tuan HoangMichael SchwarzeAnhe WangMichael SemenovAni IdrisMichael SinapiusAníbal Maury-RamírezMichael VigdorowitschAnibal MendesMichael WinokurAnil Krishna BattuMichael ZinigradAnil KumarMichaela GkantouAnil Kumar DasMichaela SojkovaAnil Kumar SethyMichał BatschAnil Kumar YedluriMichal BlahoAnil KunwarMichał BoćkowskiAnil Reddy MarriMichal DudekAnil YadavMichal HolubcikAniruddh VashisthMichał JurekAnish KhanMichal KelemenAnish PhilipMichał KotkowiakAnish ShivaramMichal KrbaťaAnisha D’souzaMichał KubiśAnisoara CimpeanMichal KulkaAnita HeczelMichał LandowskiAnita KwaśniewskaMichał MusiałAnita Olszówka-MyalskaMichal NiemczakAnita TarbukMichał NowickiAnita Trenczek-ZajacMichal PetruAnjan BediMichał RachwalskiAnjan SilMichał RejdakAnjanapura V. RaghuMichal RymsAnjela Koblischka-VenevaMichal ŠajgalíkAnka Trajkovska PetkoskaMichał SarulAnke KlingnerMichal SedlačíkAnkica ŠarićMichal SejnohaAnkit GuptaMichal ŠoferAnkit SharmaMichał Stefan LachAnkit SonthaliaMichał SzotaAnkur BhogayataMichal SzuckiAnkur ChauhanMichał TarnowskiAnkur GuptaMichel ArrigoniAnkur SoodMichel Picanço OliveiraAnmin ZhengMichel ShengoAnming HuMichela BotticelliAnna AbramovaMichela NocettiAnna BacarditMichela RiccaAnna BastrzykMichele BiciAnna CardinaleMichele BonninAnna ChurakovaMichele CacciaAnna D. ZervakiMichele D’AmatoAnna DanihelováMichele Di CosolaAnna DettlaffMichele DondiAnna DobkowskaMichele Fabio GranataAnna DziubińskaMichele FerrariAnna Erkiert-PolgujMichele MaglioneAnna Fajdek-BiedaMichele MonnoAnna FakhardoMichele PaolantonioAnna Georgievna KnyazevaMichelina CatauroAnna GóralMichelle SalviaAnna GuzanováMichio ShimotomaiAnna JurowskaMicka BahAnna KawalekMieczysław JałochowskiAnna KnaislováMieczyslaw PietrzykAnna Knitter-PiątkowskaMien Van TranAnna KonstanciakMieszko WieckiewiczAnna KornyushchenkoMiguel A. García-SánchezAnna KostyukMiguel A. Montes MoránAnna KowalewskaMiguel A. OrtegaAnna KulakowskaMiguel A.G. ArandaAnna KusiorMiguel Adolfo PonceAnna ŁapińskaMiguel Andrés Hernández RodríguezAnna M. BrudziszMiguel Angel Baltazar-ZamoraAnna M. GrabiecMiguel Angel De La RubiaAnna M. OzerovaMiguel Ángel Fernández-BarreraAnna M. RakoczyMiguel Angel Gracia PinillaAnna M. SalviMiguel Ángel Hidalgo SalazarAnna Małgorzata KawałekMiguel Angel Olivares-RoblesAnna Maria SzekeresMiguel Ángel Ruíz CabreraAnna MarkiewiczMiguel Ángel SanjuánAnna MkrtchyanMiguel Angelo SchettinoAnna MłynarczykowskaMiguel Aythami Ayuso SebastianAnna NieróbcaMiguel BravoAnna NocivinMiguel Castro-ColinAnna PajdakMiguel CiriaAnna Paradowska-StolarzMiguel Gisbert-GarzaránAnna PiccirilloMiguel Gomez-HerasAnna RabajczykMiguel LorenzoAnna RudawskaMiguel Ponce-VargasAnna S. SemisalovaMiguel Ramon Pecci LLoretAnna ShelyugMiguel Torres RodríguezAnna SpadloMiha LukšičAnna StarczewskaMihael BrunčkoAnna StepienMihael BučkoAnna SygudaMihaela BacalumAnna Szymczak GraczykMihaela CosnitaAnna TurekMihaela M. TrandafirAnna VinattieriMihaela Raluca CondruzAnna WróblewskaMihaela SofronieAnna Zawada-TomkiewiczMihai AnastasescuAnna ZykovaMihai EftimieAnnalisa FrancoMihai JuncarAnnalisa PaoloneMihai OaneAnnamaria GerardinoMihai SăndulescuAnnarita StringaroMihai TircoleaAnne HofmeisterMihai Valentin PredoiAnne TalneauMihai Victor PricopAnnie-claude Bayeul-lainéMihaiela IliescuAnouar El MagriMihai-Ionut SturzaAnselm HornMihail LunguAnshul YadavMihail SecuAnshuman PatraMihail TarassovAntal RockenbauerMihee HongAntal UdvardyMihir PendharkarAnte PrkićMihnea-Ioan NicolescuAnthony HarkerMiho JeongAnthony J. SmithMijia YangAnthony ThuaultMijo NikolicAntolino GallegoMike BambachAnton BergantMikhail A. Eron’YanAnton ChepurnenkoMikhail A. Il’yushinAnton KhrustalyovMikhaïl A. LebyodkinAnton M. KuzminMikhail BeklemishevAnton ManakhovMikhail BelogolovskiiAnton NikiforovMikhail DunaevskiyAnton ShalyginMikhail G. BrikAnton TiutiunnykMikhail GuzevAnton V. PoluninMikhail KryuchkovAnton Yu. NalivaikoMikhail Mikhailovich SkripalenkoAnton ZubrikMikhail O. EreminAntonella RizzoMikhail P. KozochkinAntoni CladeraMikhail P. Kuz’minAntonia Martínez-LuévanosMikhail PetrovAntonia ResslerMikhail PopovAntonietta Lo ConteMikhail ProskurninAntonietta M. TaurinoMikhail R. BaklanovAntonietta SicilianoMikhail SeleznevAntonija TadinMikhail ShamoninAntonina I. KarlinaMikhail ShestakovAntonina MaizelisMikhail SlobodyanAntonino PolimenoMikhail StarostenkovAntónio AlmeidaMikhail TurbakovAntonio Alvarez Fernandez-BalbuenaMikhail V. DorokhinAntonio AngeloMikhail V. GolubAntonio ApicellaMikhail Y. ElistratkinAntonio BacciagliaMikhail Yu. SkripkinAntonio Bernabé-AntonioMikihito TakenakaAntonio BrunettiMikio KumitaAntonio CapezzaMikko Markus HupaAntónio Castelo FilhoMiklós BakAntonio CentofantiMikołaj BernasowskiAntonio ConcilioMikolaj OettingenAntonio ConsoliMilad BahamirianAntonio D’AndreaMilad JanalipourAntonio Del VecchioMilad MoradianAntonio Di BartolomeoMilad SheydaeiAntonio Doménech-CarbóMilada PezoAntonio Dominguez-AlfaroMilan B. RadovanovićAntonio E. Salas-ReyesMilan BanićAntonio Elia ForteMilan BoušaAntonio Esau Del Rio CastilloMilan ČervenkaAntonio FabaMilan K. SadanAntonio FerraroMilan KadnárAntonio FormisanoMilan KragovićAntonio GaglianoMilan PaleiAntonio GesualdoMilan RackovAntónio GinjeiraMilán SzőriAntonio Giovanni SolimandoMilan TomaAntonio GloriaMilan ZeljkovicAntonio GrandeMile IvandaAntonio GrecoMilena DraganovaAntonio H. De AzaMilena KušnerováAntónio HS DelgadoMilena PavlíkováAntonio IsalgueMilena RajicAntonio Jesus FernandezMilica AcimovicAntónio João CruzMilica SpasojevicAntonio LeoMilica VasicAntonio LocciMiljana MirkovićAntonio Lopez UcedaMiljana PricaAntónio Manuel Gonçalves PedroMilka PotrebićAntonio Mario LocciMilos B. DjukicAntónio Mário PereiraMiloš MadićAntonio MonteparaMiloš MilovančevićAntonio MonzónMilos NenadovicAntonio PantanoMiloš OgnjanovićAntónio PereiraMiloš RadosavljevićAntonio PerejonMilosh HuberAntonio PiccininniMiłosz GrodzickiAntonio PinedaMiłosz HuberAntonio Riul Jr.Miloud IbrirAntonio Riveiro RodriguezMiloud RahmouneAntonio Rodero SerranoMin HongAntonio S. B. SombraMin Jae ParkAntonio SantagataMin LuoAntonio SpinarelliMin ParkAntonio ViscusiMin QiaoAntonio ZuorroMin Wook LeeAntonios N. PapadopoulosMin XiongAntonis PeppasMin YangAntony AnanthMin YuAntoon J.M. LigtenbergMin ZhuAntreas KantarosMina M. MedićAnuar IshakMina TadrosAnubhav SrivastavaMincheol HanAnuj KumarMindaugas GedvilasAnuja YadavMindy LevineAnup PandithMinfang ZhangAnup PaulMing HuAnuraag BoddupalliMing HuangAnuradha KhetwalMing JiangAnurag GuptaMing LiAnurag PisupatiMing LiuAnurag SatpathyMing TangAnurag SharmaMing XiaoAnuraj KshirsagarMingfeng KaiAnuraj VaryambathMinggang XiaAnush ChandrappaMinghao LiuAnusha AdityaMinghao ZhaoAnuwat AttachaiyawuthMinghe ZhangAnwar KhitabMing-Hsi ChiangAnwar SaeedMinglei QuAnxin MaMingli CaoAnyanee KamkaewMingliang WangAnyang WangMingliang ZhangAo LiMingming LiAphichart RodchanarowanMingming TongApip AmrullahMingqing WangApostolos KorlosMingren ShenApurv YadavMingshuai HuoAraa Mebdir HoliMingtao ChenArabinda MeherMingtao WangArackal Narayanan JinoopMingwen BaiAram SaharianMingxing GuoArash AhmadivandMingxuan LuArash JamaliMingyuan ZhengArash Yazdanpanah-GoharriziMingzhe AnAravin Prince PeriyasamyMingzhu LiArbaz SajjadMinh Phung VanArbind PrasadMinh Tan Ton-ThatArcady DyskinMinh Vien LeArdiansyah TaufikMinh-Dung TruongAref Abbasi MoudMin-Ho HongAref Sadeghi-NikMinho O.Argaw GurmuMinh-Tan HaArianit RekaMinjung KangArianna De MoriMinoru OsanaiArie WardhonoMinseok KimAriel Colaço De OliveiraMinsu JungArindam PramanikMinsub ChungAriosto MedinaMiorita UngureanuAris GiannakasMircea DragomanAristos ChristouMircea Horia ŢiereanArit DasMircea NasuiAritra GhoshMircea TampaArivazhagan NatarajanMirco PeronArjun Prasad TiwariMirela DraganArjun SinghMirela GalićArkadeb MukhopadhyayMirela MatićArkadij SobolevMirela PanainteArkadiusz ArtyszakMirela-Fernanda ZaltariovArkadiusz BednarzMirella Gutiérrez-ArzaluzArkadiusz CiesielskiMiriam F. ScelzaArkadiusz SzarekMiriding MutailipuArkady FinkelsteinMirjana ĐurasevićArkady SerikovMirjana KostićArkajit MandalMirko KarizArkamitra KarMirko PoljakArkom KaewrawangMiroslav BlatnickýArlin Bruno TchambaMiroslav DramicaninArmagan KaramanliMiroslav GombarArman Amani BabadiMiroslav ManasArman HoseinpurMiroslav NěmecArman ZarebidakiMiroslav PiskaArmando Aguilar-MeléndezMiroslav PremrovArmando Roman-FloresMiroslav VarinyArmando SalinasMiroslav ZecevicArmin RajabiMirosław DudekArnab Sen GuptaMiroslaw GalazkaArnaldo CasalottiMiroslaw KordosArnaldo Leal-JuniorMirosław KruszewskiArnaud CaronMirosław KwiatkowskiArnaud DuchosalMiroslaw RuckiArnaud MacadreMirosław S. BonekArnaud PerrotMiroslaw SzalaArno SchindlmayrMiroslaw SzybowiczArooran SounthararajahMirosław TupajArpad Mihai RostasMirosław WyszkowskiArsalan KhushnoodMiroslawa PawytaArseni V. UshakovMirza RubelArseniy KiryakovMirza Wasif BaigArshad JamalMisun KangArshad RiazMitar SimićArslan AkbarMithil MazumderArtem A. SelyutinMithilesh DikshitArtem EreminMithun PalitArtem MarchenkovMitja PetricArtem MarikutsaMitsuaki MatsuokaArtem RogachevMitsuhiro ShigeishiArtem SharkoMitsuru TashiroArtemi CerdaMitsuyasu IwanamiArtemiy AborkinMiusi ShiArthi GopalakrishnanMizan AhmedArtit ChingsungnoenMizuho KondoArtur Camposo PereiraMladomir MilutinovićArtur ChrobakMochamad AsrofiArtur CzupryńskiMoghadam Mehrdad AbdiArtur J. M. ValenteMoh YaseenArtur KhannanovMohamad Fahrul Radzi HanifahArtur KrzyżakMohamad Faiz ZainuddinArtur Mariano De Sousa MalafaiaMohamad Fakhrul Ridhwan SamsudinArtur NowoświatMohamad GhaffarianArtūras ŽalgaMohamad Hafiz MamatArturo GonzalezMohamad ShahgholiArturo Rodríguez-GómezMohamad Yusri AmanArturo Sanchez-PerezMohamad Zul Hilmey MakmudArturs BundulisMohamadreza AfrasiabiArtyom BogdanovMohamadreza ZarastvandArul AndersonMohamed A. BasyooniArun ArjunanMohamed A. ShenashenArun ButreddyMohamed AbdelazeemArun GhoshMohamed Abdelsabour FahmyArun K. SinghMohamed AbdelwahabArun Kumar TeotiaMohamed Abuelseoud Abdelzaher AliArun MuraliMohamed AdelArun PatilMohamed Ahmed TahaArun Prabhu RameshbabuMohamed AldlemyArun Prakash PeriyasamyMohamed AwadArun ThirumuruganMohamed AyeldeenAruna Kumar BarickMohamed B. ZakariaArunkumar BongaleMohamed BakarArxel De LeónMohamed BassyouniAryan AfzalianMohamed EgizaAsaad MigotMohamed El GarahAsad HanifMohamed El-NewehyAsad Ullah KhanMohamed ElsafiAsadullah KhalidMohamed EltaherAsadullah MemonMohamed Emam-IsmailAsandulesa MihaiMohamed G. ShehataAseem ChawlaMohamed Gamal MohamedAsfandyar KhanMohamed GoudaAsgar KayanMohamed H. MussaAsghar Heydari AstaraeeMohamed Hamdy ElsafiAshane FernandoMohamed HarhashAshfaque Ahmed JhatialMohamed Hechmi El OuniAshis PrajapiMohamed IbrahiemAshish GoyalMohamed M. El-Sayed SelemanAshish KulkarniMohamed MeghilAshish SrivastavaMohamed Mehawed AbdellatifAshkan BighamMohamed Mohamed Zaky AhmedAshkan Nabavi-PelesaraeiMohamed MostafaAshok Kumar Kumar SahooMohamed Musa HanafiAshok YadavMohamed Rafik QureshiAshraf AliMohamed SadikiAshraf El-BindaryMohamed SalaheldeenAshton Keith CowanMohamed SharafeldinAshutosh Ashutosh KumarMohamed SolimanAshutosh KumarMohamed SuleimanAshutosh SharmaMohamed SulymanAshwani KumarMohamed Thariq SultanAshwin Kumar MyakalwarMohamed ZbairAshwin Kumar SaikumarMohamed ZellaguiAshwin RautMohammad Abdul MannanAshwini A. SalunkheMohammad AbediAsim JilaniMohammad Afsar UddinAsit Kumar GainMohammad AkramiAsla Abdullah Al-ZahraniMohammad AlamAsma KhoobiMohammad AlbakriAsma PerveenMohammad Alhuyi NazariAsmaa M. FahimMohammad Ali MosaberpanahAsmita BanstolaMohammad Ali RezvaniAsta TamulevičienėMohammad AlihaAstrid L. GiraldoMohammad AlrwashdehAswin Kumar AnbalaganMohammad Amin Hariri-ArdebiliAsyraf RizalMohammad ArefiAtaç BascetinMohammad Asaduzzaman ChowdhuryAtakan TekgülMohammad Bagher RahmaniAtchudan RajiMohammad BaraheniAthanasia PetalaMohammad BazzazAthanasia VarvaresouMohammad Ehtisham KhanAthanasios C. MitropoulosMohammad El KhatibAthanasios KatsourasMohammad ErfanmaneshAthanasios TiliakosMohammad FasihiAtheer AwadMohammad Fatehi MarjiAthinarayanan SundaresanMohammad GolmohammadAtichat WongkoblapMohammad HajeerAtiđa SelmaniMohammad HanifehzadehAtila KumbasarogluMohammad HashemiAtin PramanikMohammad HassanAtiphan PimkhaokhamMohammad Hazhir MozaffariAtiq Ur RehmanMohammad Heidari-RaraniAtsunobu MasunoMohammad HojjatiAtsushi SugiharaMohammad Hossein PaydarAttila BaksaMohammad I. HossainAttila BendeMohammad Ilyas KhanAttila BonyárMohammad Iman KhodakaramiAttila CsíkMohammad IslamAttila GéczyMohammad IsmailAttila GergelyMohammad IzadiAtul BabbarMohammad Javad MoradiAtul SharmaMohammad KashfiAudrey PotdevinMohammad Kazem Hassanzadeh-AghdamAudrius ŽundaMohammad Kazem MohammadiAugusto Cannone FalchettoMohammad KhanehbadAugusto Cesar Da Silva BezerraMohammad Marvi-MashhadiAura RusuMohammad MirzaieAura-Catalina MocanuMohammad Mizanur RahmanAurang ZaibMohammad MomeniAurea Immacolata LumbauMohammad NajjarAurel DiaconMohammad Norouzi BanisAurel Mihail TituMohammad Qasim ShaikhAurelia Cristina NechiforMohammad Rahimi-GorjiAurelia VisaMohammad RavandiAurelian Sorin PascaMohammad Reza JandaghiAurélie Julian-JankowiakMohammad Reza KhalesiAurélien DoitrandMohammad Reza Zamani-MeymianAurelio García-ValenzuelaMohammad Ruhul Amin BhuiyanAuxi BarbudoMohammad S. RouhiAvanish MishraMohammad SaadatiAvik Kumar DasMohammad Salim KaiserAvik SamantaMohammad SharifipourAvinash LakshmikanthanMohammad Sultan AlakhaliAvinash P. ManianMohammad Taha MasturaAvinkrishnan A. VijayachandranMohammad Tajul IslamAvishek ChandaMohammad TaufikAvneesh KumarMohammad TauviqirrahmanAvni BerishaMohammad ThaljiAvto TavkhelidzeMohammad YaghoubAwal NoorMohammad Yazdi HarminAwni QasaimehMohammad YekrangniaAxel GriescheMohammad YosofvandAxel MarquardtMohammad Yousaf AshfaqAxel PramannMohammad Z. KabirAyad M. TakhakhMohammad ZaidAyar PooyanMohammadmehdi Shahzamanian SichaniAyaz AhmadMohammadreza DaroonparvarAybüke İsbir TuranMohammadreza IzadifarAydan YeltikMohammadsadegh BarkhordariAydi AbdelkarimMohammed A. KhelkhalAydin Bordbar-KhiabaniMohammed A. TahaAydin GülsesMohammed Ali DheyabAyla BilginMohammed AlsultanAylin M. DeliormanliMohammed F. HamzaAyman El-FahamMohammed FattahAyman El-ZohairyMohammed GrawishAyman H. KamelMohammed HawashAyman M. MostafaMohammed Ibrahim SayyedAyman MostafaMohammed KoubitiAymen AssadiMohammed M. RahmanAyoub MoghadamMohammed Metwally GomaaAyse Aslan CanpolatMohammed Mu’azuAyşegül ErdoğanMohammed Nadeem BijleAyub ElahiMohammed Rafi ShaikAzad A. MohammadMohammed RasheedAzat TipeevMohammed Rizwan KhanAzeem MustafaMohammed S. AlsuhybaniAzhar Ali ZafarMohammed S. GumaanAzhar HussainMohammed SaidAzim GökçeMohammed Salah NasrAziz KhanMohammed SarhanAziz Ullah AwanMohammed SayedAzizi HosseinMohammed Seddik MeddahAzlin F. OsmanMohammed SobhyAzmah HanimMohammed Y. AbdellahAznan IsmailMohammed Y. EmranAzusa N. HattoriMohammed Z. SwalmehB. C. ChanyalMohammednoor Khalil AltarawnehB. C. PrasannakumaraMohammod AminuzzamanB. NagarajaganeshMohandoss SonaimuthuBabak BagheriMohanraj MurugesanBabak ChehroudiMohaseen TamboliBabak EslamiMohd AhmedBabar AliMohd BuraidahBabar ShabbirMohd Hafiz Mohd ZaidBadawi AnisMohd Hazim Mohamad AminiBadr M. ThamerMohd Idzat IdrisBadreddine RatniMohd Mahadi HalimBagh AliMohd Mustafa Al Bakri AbdullahBahador BahramiMohd Mustafa Awang KechikBahareh AzimiMohd Muzamir MahatBahareh Ghane MotlaghMohd Najib Mohd YasinBahri BaşaranMohd Natashah NorizanBaichang LiMohd Nazri AhmadBaiquan LiuMohd Nor Faiz NorrrahimBaizeng FangMohd Nurazzi NorizanBakalar GoranMohd RafatullahBakhtiar KhanMohd Raizamzamani Md ZainBaksha ArivazhaganMohd Ridzwan Abd HalimBalaji RengarajanMohd RosliBalakrishna MurthyMohd Salahuddin Mohd BasriBalakumar S.Mohd Shahbudin MasdarBalaraman VedhanarayananMohd Shahneel SaharudinBalasankar Meera PriyadarshiniMohd Sharif NurulakmalBalasubramaniam StalinMohd ShkirBalázs IllésMohd Shukor SallehBalázs NagyMohd Shukur Zainol AbidinBalazs TothMohd UbaidullahBaljeet SinghMohit SharmaBaltatu SimonaMohri FoudilBaltej Singh RupalMohsan JelaniBaltus Cornelius BonseMohsen AbbaspourBangda WangMohsen MhadhbiBaokuan LiMohsen PadervandBaoping GongMohsen Saffari PourBaoyang LuMohsen YazdanianBappa AcherjeeMohsen ZandiBarbara BonelliMohsin JavedBarbara CorteseMohsin Usman QureshiBarbara GawdzikMoisés Oswaldo Bustamante RúaBarbara GhinassiMojisola UsikaluBarbara HajdukMojtaba AsadiBarbara JadachMojtaba EsmailzadehBarbara KosmowskaMojtaba GhateeBarbara KozubMojtaba JafarianBarbara ŁapińskaMojtaba Lezgy-NazargahBarbara LisMokarram HossainBarbara MiroslawMolham EyadehBarbara PanunziMona Al-ShemyBarbara PawłowskaMona Aly AbbassyBarbara PietruszkaMoneeb GenedyBarbara RuffinoMonia VadrucciBarbara S. BezerraMonica GanioBarbara Šetina BatičMonica IordacheBarbara Slomka-SlupikMonica TonelliBarbara SurowskaMonica Villaquirán-CaicedoBarbara WlodarczykMonika BosackaBari KlaudioMonika FurkoBaris Baykant AlagozMonika GwoździkBaris BiniciMonika JenkoBaris SayinMonika KarkováBarkat KhanMonika MichaelisBarrie MintzMonika NaumowiczBarry LavineMonika PooniaBartek KaplanMonika ZajemskaBartłomiej AmbrożkiewiczMoniruddoza AshirBartłomiej CieślikMonssef Drissi-HabtiBartłomiej GórskiMontserrat TobajasBartlomiej JankiewiczMood MohanBartłomiej JeżMoon-Jo KimBartłomiej ZapotocznyMoorthi PichumaniBartolomeo MegnaMorad M. El-HendawyBartosz FikusMoreno LelliBartosz GurzędaMoreno MarcelliniBartosz HandkeMorgan HawkerBartosz TrawińskiMorgane Dos SantosBasant A. AliMoritz BraunBasant RoondheMoritz LiesegangBasim Mohammad Ismail Abu-JdayilMortaza Saeidi-JavashBassam A. TayehMorteza MohammadiBassiouny SalehMorteza Sasani GhamsariBasuraj BhowmikMorteza TaheriBatsuren DorjsurenMoruf YusufBayram GündüzMosab KaseemBeata BorakMoses KarakouzianBeata Butruk-RaszejaMoses M. SolomonBeata JaworskaMoshood OnifadeBeata KrupińskaMostafa AsadizadehBeata KurcMostafa El-ShafieBeata Łaźniewska-PiekarczykMostafa HabibiBeata Leszczyńska-MadejMostafa M. A. KhaterBeata StrzemieckaMotasem AlazaizaBeata ZimaMoti Lal RinawaBeatrice SalieriMotoki OkamotoBeatriz GonzálezMotoki UedaBeatriz Martin-PerezMotonobu TomodaBegoña SavoiniMouhsine GalaiBegoña VerdejoMourad KeddamBehbod GhalamkariMourad NachtaneBehin JamshidMourouzis PetrosBehrooz BeidokhtiMousa Hamouda M.Behzad AvishanMoussa OuakkiBehzad NiroumandMoustafa DarwishBehzad V. FarahaniMoustafa MahmoudBehzad VaferiMoustafa Mahmoud Yousry ZaghloulBelal DawoudMriganka Shekhar ChakiBelén Onecha PérezMrinmoy GaraiBen AlcockMsomi VelaphiBen B. XuMu Chun WangBen Souf AmineMu Ku ChenBence Tamás SzabóMu’tasim Abdel-jaberBénédicte LepoittevinMucahit YilmazBenedikt KötterMudaser UllahBengi UsluMugahed AmranBenito Serrano RosalesMuhamad Kamil YaakobBenjamin E. MacdonaldMuhammad A. DalhatBenjamin Le OuayMuhammad AamirBenjamin MitchellMuhammad AbdullahBenjamin TawiahMuhammad Adly Rahandi LubisBenjamín ValdezMuhammad AdnanBenjaram Mahipal Vijayasimha ReddyMuhammad Adnan AsgharBenoit PicouxMuhammad AfzaalBenqing ZhouMuhammad Ahsan SaeedBenson KariukiMuhammad AjazBeow Keat YapMuhammad Akhsin MuflikhunBerceste GülerMuhammad AliBere PaulMuhammad Ali ButtBernadette Emoke TelekyMuhammad Ali SikandarBernardeta DębskaMuhammad Altaf NazirBernardo Gonçalves AlmeidaMuhammad AmjadBernd-Arno BehrensMuhammad Aniq ShazniBernhard J. HoendersMuhammad Arif (Abdul WaliBernhard MiddendorfMuhammad Arif (University Bernuzzi ClaudioMuhammad ArshadBertrand CambouMuhammad Asad JanBertrand FournierMuhammad AshfaqBesarion MeskhiMuhammad AsifBettina CaminMuhammad Asif Zahoor RajaBettina WollankeMuhammad AswinBetül Aldemir DikiciMuhammad Awais JavedBetül Çelebi-SaltikMuhammad AyazBhadra Prasad PokharelMuhammad AyyoobBharat SinghMuhammad Azeem ArshadBharath RamaswamyMuhammad Baseer HaiderBharati NeelamrajuMuhammad Bilal Bilal ShakoorBhausaheb TawadeMuhammad Bilal HanifBhavneet KaurMuhammad FahadBhavya B. KrishnaMuhammad Faisal JavedBhowmick MithunMuhammad Fayzan ShakirBhupendra JoshiMuhammad Hafiz HassanBiagio RaponeMuhammad HarrisBiagio SantellaMuhammad HumayunBiao FuMuhammad IkramBiao GengMuhammad Imam AmmarullahBidikoudi MariaMuhammad Imran (Pakistan)Bijender Kumar‪Muhammad Imran (Qatar)Bilal AhmadMuhammad Imran MalikBilal KhanMuhammad IqbalBilel SelmiMuhammad Irfan SiyalBilge Coşkuner FilizMuhammad Irfan-ul-HassanBilge SaruhanMuhammad JavedBilgin KaftanoĝluMuhammad JunaidBiljana ArsicMuhammad Junaid MunirBiljana BujanovicMuhammad Kalimur RahmanBiljana ŠljukićMuhammad KashifBill DavidsMuhammad KhalidBillel ChenitiMuhammad Khan (Malaysia)Bin HongMuhammad Khan (Saudi Arabia)Bin HuangMuhammad Masood RafiBin JiangMuhammad MuzamilBin LiMuhammad NabeelBin LiuMuhammad NaeemBin LüMuhammad NasirBin WangMuhammad Nasir MuradBin WuMuhammad NaveedBin YangMuhammad Nurul IslamBin YuMuhammad Prisla KamilBin YuanMuhammad QaiserBin ZhangMuhammad QamarBindiganavile Anand PraveenaMuhammad RaheelBindu SubhadraMuhammad Raziq Rahimi KoohBindu Vijaya RamnathMuhammad Roil BiladBing DuMuhammad SamiuddinBing LiMuhammad SaqibBing LiuMuhammad ShafiqueBing LuMuhammad ShahidBing YeMuhammad Shahzad AslamBinglin LaiMuhammad Shahzad KamalBingsheng LiMuhammad Shakeel AhmadBingxin ZhaoMuhammad SohailBinoy MaitiMuhammad Sohail MalikBinsheng ZhangMuhammad Sohail ZafarBipasha BoseMuhammad Sufyan JavedBiranchi PandaMuhammad SulaimanBirgit LangeMuhammad SultanBishnu GautamMuhammad TahirBishnubrata PatraMuhammad TawalbehBishweshwar PantMuhammad Tayyab NaqashBissera PilichevaMuhammad Umar FarooqBiswa Nath BhadraMuhammad UsmanBiswajit BhattacharyyaMuhammad Usman HameedBiswajit PramanickMuhammad Usman HanifBiswajit SahaMuhammad Wakil ShahzadBjörn ErikssonMuhammad YasirBjørn SkallerudMuhammad ZafarBlair TuttleMuhammad Zaheer SaleemBlas ChaparroMuhammad ZahidBlaž BelecMuhammad Zeeshan KhalidBlaza StojanovicMuhammed Naseem AkhtarBłażej ScheibeMuhammet CeylanBlessen Skariah ThomasMuhannad Al-WailyBluma G SoaresMuharrem KaraaslanBo ChenMuhsin J. JweejBo FengMuhtar MuhtarBo FuMuidh H. AlheshibriBo GaoMujahid FaridBo LuMujtahid KaavessinaBo QuMukesh Kumar (MaharshiBo SongMukesh Kumar (Shree Guru Bo WangMukhtiyar SinghBo WuMukund TantakBob HowellMunder BilemaBobo YangMuneeb IrshadBochuan TanMuneer BaigBodor MariusMunise Didem DemirbaşBogdan A. SavaMunteanu CorneliuBogdan Alexandr SavaMurali DamaBogdan Alexandru ChiritaMurali Mohan CheepuBogdan AntoszewskiMuralimohan CheepuBogdan BitaMuralithran KuttyBogdan Cătălin ȘerbanMurat Kaya YapiciBogdan CulicMurat Olgaç KangalBogdan Ene-IordacheMurat TürközBogdan GilevMurat YaylacıBogdan IstrateMurillo-Gómez FabiánBogdan KowalskiMurni Nazira SarianBogdan Marian TofanicaMurtaza HasanBogdan NedićMurtaza TambuwalaBogdan RutkowskiMurthy ChavaliBogdan SłodkiMurugan Senthil Mani RajanBogdan VasylivMurugan VeerapandianBogdan Viorel NeamtuMurugathas ThanihaichelvanBogdan WyrwasMusa AdamuBogdana MituMushriq AbidBohan XuMuslim AbdurrahmanBoin TaiMuslum DemirBoitumelo Joyce MatsosoMustafa BayramBojan MiljevicMustafa CinarBojana M. RadojkovićMustafa GünayBojana VoncinaMustafa KuntogluBokhimi XimMustafa Mamduh Mustafa AwdBolesław SzadkowskiMustafa ÖzerBolutife OlofinjanaMustafa Serdar KarakaşBonaccorsi Di Patti Maria CarmelaMustapha JouiadBong Guen HongMustapha RaihaneBongchul KangMuthaiah ShellaiahBonnie R. AntounMuthia ElmaBora GaripcanMuthukumar ThangaveluBoris B. KhinaMuthuraman Meenakshi SundaramBoris ErshovMutian HuaBoris GolmanMuttaqin HasanBoris KrichevtsovMuzamil KhatriBoris MajaronMuzamir IsaBorislav SavkovicMy Loan Phung LeBor-Shiunn LeeMythili PrakasamBorut ŽužekMyung Su YiBo-Shiuan LiN. AnandBo-Tau LiuN. KantiranisBouafia AbderrhmaneN. Rajesh Jesudoss HynesBouraoui IlahiNa GongBowen GongNabil DaghboujBowen LiangNabil HossineyBoyko G. TsyntsarskiNabil KherroubaBoyko TsyntsarskiNabil NemerBoyong KimNaďa BeronskáBožana ČolovićNadeem A. KhanBožana Lončar BrzakNadejda HorchidanBozena Burtan-GwizdalaNader DolatabadiBożena CzechNader ShehataBożena JarząbekNadezda StankovicBożena Szczucka-LasotaNadezhda A. NebogatikovaBozhidar StefanovNadezhda A. ZhukBožidar ŠarlerNadezhda DudovaBozo VazicNadezhda PolekhinaBradley J. RothNadia AkramBrain ViaNadia ArrousseBram VandorenNadia LicciardelloBrandon Scott TaysomNadia Mahmoudi KhatirBranislav MarkovićNadia MartuccielloBranko ŠtrbacNadia SaoulaBretislav SkrbekNadiia MamekaBrian E. GrottkauNadja KrögerBrian J. BellottNadlene RazaliBrian MeldeNaechul ShinBriana AguilaNafisa GullBrigida AlfanoNafisah OsmanBrigit MittelmanNagalakshmi RamamoorthiBrigitte ClausenNagaraj NagaprasadBrigitte GrosgogeatNagaraj NandihalliBritta BergfeldtNagaraj P. ShettiBrock HedegaardNagaraj P. Shetti ShettiBronson PhillippaNagaraj R. BanaprmathBrubeck Lee FreemanNagaraju KerruBruna CallegariNagaraju PothukanuriBruna SinjariNagasivamuni BalasubramaniBruno AzambreNagavendra KommineniBruno Rafael MachadoNagentrau MuniandyBruno Sena Da FonsecaNahla Naji HilalBruno SoaresNaida AdemovićBruno TiburcioNaiguang WangBruno TrindadeNail M. ShavaleevBruno WeiseNajam Ul HassanBryan M. WongNaji KharoufBryan WongNajm M. Al-HosinyBulat N. GalimzyanovNalladurai KaliyanBulent AktasNallaperumal NallamuthuBundet BoekfaNam V. NguyenBungo OchiaiNambu KoichiroBünyamin ÇiçekNan ZengBurachat ChatveeraNancy LinBurak GerisliogluNand Gosvami NityaBurak TüzünNanda Gopal SahooBurak YönNaofumi KozaiBurduhos-Nergis Diana-PetronelaNaoufel Ben HamadiBushra JabarNaoum KarchevBuyung KosasihNaoya YamaguchiBwalya A. WitikaNaoyuki NomuraByeng Dong YounNaraindas BheelByeongil KimNarasimha Rao PillalamarriByung Jun JungNarayanan VenkateshwaranByung-Joo KimNarayanasamy Sabari ArulByungkyun KangNarcis BârsanC. K. SumeshNarcís MestresC. R. RejeeshNarendra KumarCaglar YalcinkayaNaresh DuvvaCaifu QianNaresh KumarCaihong XueNaresh Kumar MarojuCaihua ZhouNareshbabu KamathamCaisheng WuNaresworo NugrohoCamelia CerbuNarihito OkadaCamelia Elena LuchianNariman AshrafiCamilo Zamora-LedezmaNariman EnikeevCan GungorenNarinder SinghCan JiangNarmina O. BalayevaCanan AksoyNarsimha MamidiCandan GokceogluNarumi OhtaCandelaria Tejada-TovarNasar AhmedCândida MalçaNaseeb AhmadCanel EkeNaseem AbbasCangyu QuNaseer AhmedCanio NoceNashrul Fazli Mohd NasirCanyang ZhangNasim ChiniforushCarina BalcoşNasser Aedh AlreshidiCarl P. TrippNasser Al-NuaimiCarla GasbarriNatale PerchiazziCarla ZogheibNatalia A. WójcikCarlo Alberto BiffiNatalia AbramenkoCarlo BertoldiNatalia AtamasCarlo C. VersacéNatalia BourguignonCarlo MolardiNatalia GladkikhCarlo OlivieriNatalia Ivanovna KozhukhovaCarlo PaternosterNatalia KozhukhovaCarlo RossoNatalia OrekhovaCarlo SantulliNatalia PorotnikovaCarlos A. Martínez-HuitleNatalia S. MartynenkoCarlos Andrés Palacio GómezNataliya Borisovna PugachevaCarlos BoenteNataliya KazantsevaCarlos BoubetaNataliya SakharovaCarlos Cuevas-SuárezNataliya ShaburovaCarlos Enrique Cuevas SuárezNataliya V. KazantsevaCarlos FarinhaNatalya PolyakovaCarlos Fernando MourãoNatalya ShaburovaCarlos G. Garay ReyesNatanael Cuando-EspitiaCarlos GracianoNatarajan ArunadeviCarlos Guedes SoaresNatarajan RavikumarCarlos HernandezNataša NáprstkováCarlos Humberto MartinsNataša NastićCarlos J. CarrascoNataša ObradovićCarlos LeivaNatella EnukashvilyCarlos Manuel Alves Da SilvaNathália Beretta TomazioCarlos MarquesNathalia RamosCarlos MartinsNathalie Zink LorreCarlos Miguel MartoNathan CowiesonCarlos MoroNathan R HalcovitchCarlos MoyaNatividad Gómez-CerezoCarlos Nelson EliasNatraj YedlaCarlos Ocampo-LópezNatt MakulCarlos Pérez-Albacete MartínezNattawut OsakooCarlos Rivera-GómezNauman IjazCarlos Roberto GrandiniNaurang SainiCarlos Rolando Ríos-SoberanisNavadeep ShrivastavaCarlos Romero MoralesNavaratnarajah KuganathanCarlos RovereNavaratnarajah SathiparanCarlos Santiuste RomeroNavdeep SinghCarlos SerpaNaved MalekCarlos Torres-TorresNaveed AhmedCarlos Vargas-SalgadoNaveen Manhar ChavanCarlos ViegasNavid AslfattahiCarlos Vinicios OpeltNavid GhasemiCarlos William de Araujo PaschoalNavid Jubaer AyonCarlos-Alberto Cruz-VillarNavid SohrabiCarlotta FranciaNavneet Chandra VermaCarmela GurauNawal Mohammed DawoodCarmelo MajoranaNayan Ranjan SinghaCarmelo SgarlataNazir AhmadCarmen Cornelia GaidauNdue KanariCarmen GutiérrezNebojša BogojevićCarmen MihailescuNebojsa GnjatovicCarmen MitaNebojša NikolićCarmen SavinNectarios VidakisCarmen ZahariaNeelesh K. JainCarmine AttanasioNeelesh Kumar MehraCarol BarryNeelima MahatoCarole CharavetNeelima SatyamCaroline BorderonNeeraj SharmaCaroline MoraesNeha KondekarCarolyn M. PrimusNeha SharmaCasper AgatonNeil R. DilleyCataldo De BlasioNeith PachecoCatalin FetecauNektarios KalyvasCatalin I. PruncuNelson E. V. RodriguesCătălin MaximNelson Wilbur Pech-MayCatalin PruncuNemanja GavrilovCatalin VitelaruNemanja P. TrišovićCatalina Natalia Cheaburu YilmazNemany HanafyCatalina-Alice BrandusNenad RadovićCatarina FarinhaNenad ŠekularacCatherine AmiensNeus Jornet-MartinezCatinca SecuianuNeus VilaCattin LindaNeven BarišićCecilia MortalòNeven UkrainczykCédric BourgèsNevenka RajicCédric GiryNevzat ÖzgürCelal CakirogluNgakan Ketut Acwin DwijendraCelalettin KaradoganNghia HuynhCelia Polop JordáNgoc Hieu DinhCéline PerlotNgoc San HaCemal BasaranNgoc Vinh NguyenCenk KarakurtNgoc-Hien TranCesar A.G. ArraisNgoc-Vinh NguyenCesar Costa-VeraNguyen Dinh DucCesar JuarézNguyen Hoa HongCésar L. FolciaNguyen Huu HieuCesar O. AvellanedaNguyen Thanh TungCesar SaldiasNguyen Thi Hoang OanhCesare AtzoriNguyen Tuan HungCesare CamettiNguyen Van AnhCesare SignoriniNguyen Xuan-MungCevat YamanNhat Duc HoangCeyhun AksoyluNhon Nguyen-ThanhCezar ComanescuNi YangCezary CzosnekNicanor CimpoesuCezary KraśkiewiczNicholas A. BrakeCezary SzydlowskiNicholas DerimowCh’ng Huck YwihNicholas F. MatererChadi MaaloufNick SilikasChae-Ho YimNickolai I. KlyuiChaehoon KimNickolas D. PolychronopoulosChaganti Phaneendra KiranNickolay S. IvanovChaitanya P PuranikNickolay SibirevChaitanya Reddy ChilakamarryNicola Alberto ValenteChaiyasit BanjongprasertNicola BaldoChallapalli SubrahmanyamNicola ContuzziChaminda BandaraNicola FabrisChan Jung KimNicola MolinariChanaka NavarathnaNicolae Catalin ZoitaChanapa KongmarkNicolae PopChanchal ChakrabortyNicolás AmigoChandan PandeyNicolas BertonChander PrakashNicolas CouvratChandra B. KhatriNicolas GodbertChandra Babu NaiduNicolas JolyChandra ReedyNicolas LebonChandrabalan SasikumarNicolas R. VaxelaireChandrakant Kumar NiralaNicolás Ramos- BerdullasChandrakant SonawaneNicolas RollandChandran MasiNicolas SöhlingChandrasekhar BhojarajuNicolas SoroChandrashekhar VoshavarNicoleta Nicole RaduChang Hoon LeeNicoleta PleşuChang JunNicoleta PredaChang LiuNicoleta RaduChang Whan LeeNicoleta UngureanuChanghong CaoNicoletta DitarantoChangjin XuNicoletta PedemonteChangjun ZhouNicolo GrilliChang-Shen LinNicolò LagoChangshui ChenNidal JaradatChangtong MeiNidhi JyotsanaChangwon JeongNihat ArıkanChangyong LiuNikesh GuptaChangyoon JeongNikhil Kumar KambojChangyu ZhouNiki D. BeskouChangzhou YuanNikita ChaudharyChao ChangNikita MartyushevChao GaoNikita TepliakovChao LiuNikita TsvetovChao MengNikitin DaniilChao TanNikola SakačChao WangNikola ŠpanićChao Wei TangNikola ZdolšekChao ZhenNikolai Andreevitch SobolevChaobo HuangNikolai BoshkovChaogang LouNikolai P. MalomuzhChaomin ZhangNikolai S. PerovChaoqun XiangNikolai V. TsvetkovChaoyang LiNikolai VatinCharles LochenieNikolaj MoleCharles ReichhardtNikolaos BouropoulosCharles YousifNikolaos C. KokkinosCharnnarong SaikaewNikolaos ChalmpesChatragadda RameshNikolaos CharisiouChau Nguyen DinhNikolaos E. KarkalosChawki AwadaNikolaos KoukouzasChee Ban CheahNikolaos MargaritisChel Ken ChiamNikolaos NikolaidisChellapandian MaheswaranNikolaos PolitakosChen E TerasaNikolaos SoldatosChen HuNikolaos TapoglouChen JunNikolaos V. KantartzisChen LiNikolay BezhinChen WangNikolay DimovChen XindeNikolay DjourelovChen YunhuiNikolay DolgovChen ZhaoNikolay GorshkovChenchen YuanNikolay MchedlovCheng HuangNikolay RodkevichCheng Yong HeahNikolay ShurovCheng ZhuNikolay SmaginChengang JiNikolic MijoCheng-Chen ChenNikolina ZivaljicChengcheng TaoNikos KarayiannisChenggao LiNikos LagarosCheng-Hsun-Tony ChangNilesh GuraoChenglong MaNilesh JhaChengming JiangNileshi SarafChengyang MoNiloy KhutiaChengying BaiNils EllendtChenhui QianNilufer YesilirmakChenyang XieNima AhmadiCheol Min LeeNima Gharaei-MoghaddamCheonwoo JeongNima HaghdadiCherdsak SuksiripattanapongNima MovahediCherng-Yuan LinNimer MurshidCheryl JaworowskiNimisha Chinmay ShahChetna MohabeerNina DimchevaChew Beng SohNina FincurChi Feng LinNina G. ShelushininaChi Hou LeiNina N. ObradovicChi Ling PanNina S. SelyutinaChi Vinh NgoNina V. KanevaChi Wai KanNing LiChi Yuan YangNing Zhang (Canada)Chia Her LinNing Zhang (USA)Chia Hung HungNirajan OjhaChia Hung LeeNirav JoshiChia Lin LiNirmal MazumderChia-Jung ChoNisarat RuangsawasdiChiao-Chi LinNisha ChoudharyChiara BedonNishant Raj KapoorChiara BottaNishant RanjanChiara CastellaniNishant TripathiChiara GioncoNitesh MittalChiara MandolfinoNitin MehraChiara MolinariNitin TiwariChiara PassoniNitin WasekarChiara PortesiNiyaz MahmoodiChiara ValsecchiNiyaz Mohammad MahmoodiChiayi HuangNizar B. AlsharifChidambaram SubramanianNnanake-Abasi OffiongChie Shaan SuNobufumi UeshimaChien Wei HuangNobuo SasakiChien WernNoel KonaiChienhsin WuNoelia BajalesChih Chin YangNoemi BaldinoChih Chun HsiehNoemí EncinasChih Hao LeeNoer IlmanChih Hsien ChengNoha Abdel Mawla El-WassefyChih Liang WangNoha ElessawyChih Wei LuNonthaphong PhonphuakChih Yu ChangNoor A. SanbhalChih Yuan LinNoor Azline Mohd NasirChiheng DongNoor Saeed Khan KhattakChik Him WongNoor Zaman KhanChika UjahNoorina Hidayu JamilChin Chung WeiNoorshida AliChin San WuNor Bahiyah BabaChin Wei LaiNoradin GhadimiChin Yu LinNorazriena YusoffChing Chi HsuNorbert Krisztián KovácsChing Wen LouNorbert M. NemesChinmay SatamNorbert RadekChinmaya Kumar SahooNorbert SchwesingerChinmaya Kumar SarangiNorbert SteinfeldtChinmaya MahapatraNorbert TarjányiChinna D. BathulaNorbert Venantius KangChinnasamy RajendranNorberto FeitoChinnawut PipatpanukulNorihiro KamamichiChiradeep GhoshNoriko SataChirag Batukbhai GodiyaNorio SogawaChiranjeevi KorupalliNorma-Aurea Rangel-VázquezChithirai Pon SelvanNorman S. AllenChitiphon ChuaichamNorman ToroChittaranjan DasNorshahidatu Akmar Mohd ShohaimiChiung Wu SuNorshamsuri AliCho Pei JiangNorsuzailina Mohamed SutanCholid JunaediNorul Hisham HamidChong QiNorzilawati Binti MohamadChong WangNoto Susanto GultomChongchong TangNour F. AttaiaChonghang ZhaoNoureddine AbidiChonghui WangNoureddine ElboughdiriChongjie KangNoureddine MenadChongyang LiuNourredine ArabiChongyu ZhuNovina MalviyaChoonghyun KangNowsheen GoonooChoonsoo KimNowwarote NunthawanChris BlackmanNtuthuko Wonderboy HlongwaChris G. KarayannisNuggehalli M. RavindraChris Ying CaoNuha MashaanChris ZhangNuha S. MashaanChrisna GouwsNuman DedeoǧluChristian ApelNumpon MahayotsanunChristian GaussNunes Luiz Carlos Da SilvaChristian GriffithsNuno Monteiro AzevedoChristian HaritoNuno Ricardo Maia PeixinhoChristian HöhneNunzia GalloChristian KuklaNupur BihariChristian LenserNur Izzi YusoffChristian MartellaNuraldeen Maher Al-KhanatiChristian PariggerNurcan BuduneliChristian SpreaficoNuria AlegretChristian TantardiniNuria GarcíaChristian ZollnerNursel Altan ÖzbekChristiane ThielemannNursev ErdoganChristie Ying Kei LungNurul Akidah BaharuddinChristina VöllmeckeNurxat NurajeChristine BorchersNyoman J. WistaraChristine YoungOana AlmasanChristoforos RekatsinasOana CadarChristoph HartlOana DodunChristoph HartmannOana Dragos-PinzaruChristopher ArnuschObaid AfzalChristopher Curtis LongObeid ObeidChristopher HolmesObinna OnuaguluchiChristopher HurrenOcak Yusuf SelimChristopher KnightOctavian DanilaChristopher OshmanOctavian DuliuChristos A. GogosOctavian Narcis IonescuChristos G. PapakonstantinouOctavio Elizalde SolisChristos G. TakoudisOday Ibraheem AbdullahChristos MichailOdey AlshboulChuan LiOfelia Cornelia CorbuChuang CaiOfelia Hernández-NegreteChuang DongOğuz DoğanChuangye YaoOguzhan DemirChuanming DuOksana KoplakChuanyu SunOkto DinaryantoChuhong WangOladipo FolorunsoChul Hee LeeOlaf StefanczykChul Kyu JinOlaf Van Der SluisChun LiOlatokunbo M. OfuyatanChun Liang YehOlatunji Oladimeji OjoChun LiuOle Øystein KnudsenChun Rong LinOleg ButovChun TangOleg KhasanovChun Ying HuangOleg KovtunChung Chen TsaoOleg M. GradovChungang XuOleg ManaenkovChung-Kwei LinOleg PetuhovChung-Sung LeeOleg ShevchukChunguang YangOleg ShichalinChung-Wei YangOleg SilyukovChun-Ho LinOleg StolbovChunhui WangOleg V. GradovChunhui YangOleg V. KononenkoChunjin WangOleg V. MatvienkoChunlei YangOleg V. ZakharovChunqi WangOleg VyvenkoChunui LeeOlegas ČernašėjusChurna BhandariOleksandr BondarChuyan ZhangOleksandr KovalchukCi SathishOleksandr PshykCian HughesOleksandr TisovCihan BayindiOleksandr TomchukCíntia Helena Coury SaraceniOleksandr TrudonoshynCioclea DoruOlena I. OkhayCiro D’AmicoOlesja StarkovaCiro SantusOlesya O. KapitanovaCitlalli TiburcioOlfa KanounClaude R. PhippsOlga BoytsovaClaude VerdierOlga BylyaClaudia ContiniOlga DaneykoClaudia DettoriOlga DymshitsClaudia EsproOlga GajtkoClaudia FerrarisOlga JakšićClaudia LabiancaOlga LebedevaClaudia MazzitelliOlga M. SharonovaCláudia OliveiraOlga MusskayaClaudia R. VistasOlga NetskinaClaudia TodaroOlga SamoilovaClaudinei Dos SantosOlga SedelnikovaClaudio AmovilliOlga SmirnovaClaudio Ariel DanónOlga TkachevaClaudio GervasiOlga TrhlíkováClaudio GiardiniOlga TsivilevaClaudio OggeriOlga V. Frank-KamenetskayaClaudio Oyarzo-VeraOlga V. MalyshkinaClaudio Rossi (Italy）Olga YakovtsevaClaudio Rossi (Spain)Olga ZinovievaClaudio Téllez SotoOlha ZvirkoClaudio TestaniOliver HöfftClaudio ZannoniOliver JärvikClaudiu AciuOliver TschaunerClaudiu Constantin ManoleOliver WeicholdClaudiu NicolicescuOlivier SireCleber A. AmorimOllie Yiru YuClemens MostertOlli-Ville LaukkanenClément KellerOlukayode Samuel AkinwamideClement MugemanaOluwasogo DadaClinton AigbavboaOluwatosin AbegundeColette LacabanneOlya StoilovaColin C. SeatonOmar AlshawaColuccini CarmineOmar DagdagCongwei WangOmar FayeCongyuan ZengOmar KujanConstantin ChaliorisOmar Lopez-BotelloConstantin HochOmer CivalekConstantin OpranÖmer EkmekcioğluConstantin VahlasÖmer Işik EceConstantina KolliaOmid HosseinaeiConstantinos L. ZekiosOmid KhalajConstantinos SalmasOmid NikanConstantinos SimseridesOmid ZargarConsuelo CelestiOmkar Singh KushwahaCorina Anca SimionOmojola AwogbemiCorina J. BîrleanuOmrane BenjeddouCornel BaltaOmrane Mokhtar BenjeddouCornel SamoilaOndřej HumlCorneliu CojocaruOndřej KováříkCorneliu DorofteiOndrej KvitekCorneliu HamciucOndrej RokosCorneliu MunteanuOndrej VanekCorrie ImrieOnofrio AnnunziataCosmin CodreanOnur AlevCosmin VanceaOnur CavusogluCostantino Carlo MastinoOnur GulerCostanzo BelliniOnur GuvenCostas TsioptsiasOnur MerterCostica BejinariuOnur ÖzbekCourtney L. JenkinsOnur YilmazCourtney RoperOrhan OzdemirCraig BullOriana TrubianiCraig G. MerrettOriele PalumboCraig PrzybylaOriol PonsCrina AnastasescuOriol Rius-AyraCristhian MendozaOrion CiftjaCristian Andres Aguirre TellezOsama SaberCristian DeacOsamah A. Bin-DahmanCristian Ferreiro SantisoOsamu KamiyaCristian Gómez-RodríguezOscar Andrés Jaramillo-QuinteroCristian MateiOscar Aurelio Mendoza RealesCristian NeippÓscar Darío García GarcíaCristian Ramirez-GutierrezOscar De LucioCristian RavariuOscar Figueras-AlvarezCristian ScheauOscar Gil-CastellCristian SimionÓscar MartínCristiana Lara NunesÓscar Rodríguez-AlabandaCristiane Yumi Koga-ItoOscar Ruiz-SalgueroCristiano AlbonettiOscar ZambranoCristiano CalabrettaOseweuba Valentine OkoroCristiano J. De AndradeOskar J. BeraCristina AchimOskar SachenkovCristina Antonela BanciuOsvaldo Agustín LambriCristina ArgizOthmane AouaneCristina B. HebedaOttavia JedrkiewiczCristina BarthaOussama BaaloudjCristina BirisOvidijus ŠernasCristina CiomagaOvidiu CrisanCristina CirtoajeOvidiu Cristian OpreaCristina CortiOxana V. MagdysyukCristina DumitriuOyinbo SundayCristina Gutierrez-SanchezOzer SevimCristina Lavinia NistorOzgur PoyrazCristina MarietaOzkan GokcekayaCristina Martín DoñateOzlem Ipek Kalaoglu-AltanCristina Mihaela CampianÖzlem OyardıCristina Mihaela NicolescuOzler KarakasCristina MihaliP. Dinesh BabuCristina Moreno-DíazP. M. Durai Raj VincentCristina NicaP. S. Rama SreekanthCristina OretoP. S. Samuel Ratna KumarCristina PelinP. SevvelCristina QuílezP. Syam PrasadCristina RenziP. V. RamanaCristina SchreinerPablo Álvarez-AlonsoCristina Sofia Dos Santos NevesPablo CaraccioloCristina StancuPablo García-FogedaCristina Surdu-BobPablo Lopez-AlbarranCristina Tristão AndradePablo PadillaCruceru MihaiPablo Pérez ZubiaurCsilla KádárPablo Pujadas ÁlvarezCuesta AnaPablo Rodriguez-LopezCulala Rajashekharaudayar KesavuluPablo Siqueira MeirellesCunfu WangPachaivannan PartheebanCybelle FutalanPadinharu Madathil Cyriaque Rodrigue KazePai Chen LinCyril BrochonPaisarn NaphonCyril PopovPai-Yi HsiaoCyrille RichardPal JovariCzang Ho LeePal TerekCzesław BywalskiPalamandadige K. S. C. FernandoD.M. Reddy PrasadPalanisamy MurthiDa LiPalanivel RamaswamyDa Motta Sobrinho Maurício AlvesPalaniyandi KaruppaiyaDabin GuoPalaniyappan SabarinathanDae-Cheol KoPan GongDaeho LeePan GuoDaeIk JangPan WangDae-Shik SeoPan XiaDaeyoung KimPanagiotis AngelopoulosDag NoreusPanagiotis MallisDagmar DietrichPanagiotis PoulopoulosDagmar DraganovskaPanagiotis SpyridisDagmar GregušováPanagiotis ToliasDagmara BajerPanchmatia ParthDagmara StefańskaPandiyarasan VeluswamyDagnija LocaPanida ThanyasrisungDaihui ZhangPankaj ChauhanDaiva ZeleniakienePankaj DhatrakDai-Viet N. VoPankaj Kumar (Chitkara University)Daixiu WeiPankaj Kumar (Gurukula Kangri Dakrong PissuwanPankaj Kumar (Indian Institute Dale FergusonPankaj Kumar (USA)Dali BondarPankaj Kumar ParhiDali HuangPankaj SahlotDalia KaisarlyPankaj TiwariDalia SmailienėPankajkumar N. GajjarDalibor CiprianPanneerselvam Murugapandiyan.Dalibor KocábPantelis KourosDalibor ŠatínskýPanuwat JoykladDalibor VojtěchPao Ter TeoDalius JuciusPao-Hsin LiuDallin BartonPaola AmmendolaDamayanti BagchiPaola Di MascioDamian BebenPaola GandiniDamian GizinskiPaola GinestraDamian NakoniecznyPaola SemeraroDamian NieckarzPaolo BoffanoDamian OnwudiwePaolo CapparèDamian PietrusiakPaolo CastaldoDamien LeducPaolo Di ReDamir GodecPaolo ForaboschiDamir JelaskaPaolo Francesco ManiconeDamir ValievPaolo MarianiDamir VarevacPaolo Nevone BlasiDan GanPaolo OliveroDan Jae LinPaolo S. ValvoDan LuoPaolo Sebastiano ValvoDan MarghituPaolo TrucilloDan Mihai ConstantinescuPapović SnežanaDana Adriana Iluţiu-VarvaraParamasivam ThangaduraiDana AkilbekovaParamsamy Kannan VimalathithanDana CairnsParande GururajDana Luca MotocParanjayee MandalDandan XiaParaskevas PapanikosDanial Jahed ArmaghaniParaskevi AskouniDanica Bajuk-BogdanovićParaskevi LampropoulouDaniel A. DrakeParasuraman SwaminathanDaniel AdamsParbeen Singh (USA)Daniel AntónPardeep Singh (India)Daniel AraujoParesh Chandra PradhanDaniel BarbaPartha KumbhakarDaniel BarragánPartha MaityDaniel Cenk RosenfeldParthasarathi BeraDaniel ChuchałaParthena Maria KosmidouDaniel DiazParthiban KathirvelDaniel ErrandoneaParthiban PazhamalaiDaniel Fernández-GonzálezParthiban RamasamyDaniel FerrándezParvaneh PakravanDaniel FieldParveen SihagDaniel GaertnerParviz GhoddousiDaniel GhercaPascal PuechDaniel GrosseggerPascal PuphalDaniel Hernández-CruzPasquale MarrazzoDaniel Herrera-AvellanosaPászti ZoltánDaniel Iwao SuyamaPat ClancyDaniel J. BlackwoodPatel RajkishoreDaniel J. FörsterPathapalli Venkateshwar ReddyDaniel J. MagagnoscPathik SahooDaniel KnezPatrice MélinonDaniel KytyrPatricia AraujoDaniel L.Z. CaetanoPatricia ComeauDaniel LarouchePatricia DiogoDaniel LepadatuPatricia FerrazDaniel Martinez-MarquezPatrícia Freitas RodriguesDaniel MedyńskiPatricia HuestisDaniel OhPatricia Jovicevic-KlugDaniel PapánPatricia Kara De MaeijerDaniël PeetersPatricia KrawczakDaniel PieniakPatricia Losada-PerezDaniel PietrasPatrícia M. ReisDaniel SiegmundPatrícia Schmid CalvãoDaniel SławińskiPatricia TaladrizDaniel SolaPatrick GehreDaniel ThomaziniPatrick IlgDaniel Ting Wee LooiPatrick KouotouDaniel TobołaPatrick SkrodzkiDaniel W. ElliottPatrick SturmDaniela BalanPatrik FlegnerDaniela ChicetPatrizia BernardiDaniela Cristina CulitaPatrizia BocchettaDaniela DragomanPatrycja Bałdowska-WitosDaniela DzhonovaPatrycja BogutaDaniela IannazzoPatryk RozyloDaniela KovachevaPatryk ZiółkowskiDaniela Laura BuruianăPaul C. OkonkwoDaniela LopesPaúl Carrión-MeroDaniela Lucia ChicetPaul DaviesDaniela M. CorreiaPaul DeckDaniela NovembrePaul G. RoyallDaniela NunesPaul HenshallDaniela PintoPaul J. SimmondsDaniela Šojić MerkulovPaul MotzkiDaniela SovaPaul NyangaresiDaniela SuteuPaul O. AdebamboDaniele AlmontiPaul R. EdwardsDaniele BaraldiPaul SundaramDaniele BarettinPaul Sunday NnamchiDaniele BattegazzorePaul Zavala-RiveraDaniele CangialosiPaula BarbosaDaniele ContiniPaula Corte-LeonDaniele GiansantiPaula FolinoDaniele MantionePaula Karine JorgeDaniele MurraPaula Villanueva-LlauradóDaniele PasseriPaulina Arellanes-LozadaDaniele RigonPaulina ChilimoniukDaniele RinaldiPaulina FariaDaniele ScirèPaulina Latko-DurałekDaniele SilvestriPaulina Lisiecka-GracaDaniele TammaroPaulius GriškevičiusDaniele TorselloPaulius SakalasDaniele VeclaniPaulo CachimDaniele ZampieriPaulo Cesar De MoraisDaniil A. KozlovPaulo De MatosDaniil N. BratashovPaulo De Mattos NetoDanijela ŠuputPaulo FernandesDanil PimenovPaulo J. PalmaDanila B. VasilchenkoPaulo LodiDanimir JevremovićPaulo M. S. T. De CastroDanka Labus ZlatanovicPaulo Matías La RocaDanko ĆorićPaulo ReisDanny GuzmánPaulo SáDanny ReuterPavan KumarDanny Van HemelrijckPavan Kumar PenumakalaDanqi LiPavan PujarDanrui NiPavel A. LoginovDante TolentinoPavel AbramovDanuta MatykiewiczPavel Alekseevich NikitinDanuta MiedzińskaPavel Anatolyevich NikolaychukDanying ZuoPavel AtanasoaeDaodao HuPavel ChapalaDapeng WangPavel DemakovDaria DerusovaPavel DikoDaria PoshinaPavel GinzburgDario CroccoloPavel GrigorievDario De DomenicoPavel GrudinskyDario Di NardoPavel H. Lugo-FabresDario NarducciPavel I. GrudinskyDario PasiniPavel I. KalininDario SantonocitoPavel Ivanoff ReyesDarius BacinskasPavel JandaDarius MilčiusPavel KepezhinskasDariusz BartkowskiPavel KhramtsovDariusz BartochaPavel KrivenkoDariusz BochenekPavel LukacDariusz ChocykPavel MaňasDariusz FydrychPavel NikitinDariusz GarbiecPavel NovakDariusz GóralPavel PadevětDariusz GrzybekPavel PadnyaDariusz JarzabekPavel PolachDariusz M. BielińskiPavel ProsrDariusz MikielewiczPavel ReitermanDariusz OleszakPavel RipkaDariusz PykaPavel SalvetrDariusz RozumekPavel ShurkinDariusz RydzPavel ŠkarvadaDariusz UlbrichPavel StarovoitovDariusz ZientaraPavel UrbanekDarja FeizpourPavel V. KomarovDarjan KarabasevicPavel V. PaninDarko BajićPavel V. RatnikovDarko BožićPavel Victorovich KuznetsovDarko DamjanovićPavel ZelenovskiyDarko IvančevićPavel ZháňalDarn Horng HsiaoPavle M. SpasojevicDarshit S. UpadhyayPavle SpasojevicDarya RadziukPavlina MateckovaDarya TishkevichPavlo MarkovskyDaryn BorgekovPavlo MaruschakDaryoosh DaryaeePavlo MikheenkoDasaroju GangacharyuluPavol HvizdošDasmawati MohamadPavol VadaszDastjerdi ShahriarPawan Kumar (Central UniversityDat Duy VoPawan Kumar (Indian Institute Dattatraya SubhedarPawan MishraDaulat Kumar SharmaPawandeep KaurDave WinklerPaweena PrapainainarDavid ArencónPaweł BaranowskiDavid AyrePaweł BoguszDavid BahrPaweł DunajDavid BekePaweł FalkowskiDavid BombacPaweł GrabowskiDavid BotequimPawel JezowskiDavid BrookPaweł KaczyńskiDavid CabaleiroPawel KafarskiDavid ChelazziPaweł KamińskiDavid De SmetPaweł KłosowskiDavid E. JaramilloPaweł KochmańskiDavid González-AlonsoPaweł KozakiewiczDavid J. PenneyPaweł KrzaczekDavid J. WaltersPaweł KwaśniewskiDavid Jaramillo-VigueraPaweł Leszek ŻakDavid KorenPaweł ŁukowskiDavid KovacsPaweł NowakDavid Lérida SalesPaweł OstachowskiDavid LindellPaweł PęczkowskiDavid Maria TobaldiPawel PietrusiewiczDavid MedinaPaweł SokołowskiDavid MesguichPaweł StefaniakDavid NicholsonPaweł StochDavid Nieto SimavillaPaweł StrzałkowskiDavid Revuelta CrespoPaweł SułkowiczDavid RuizPaweł SutowskiDavid Sanchez MonteroPaweł ŚwisłowskiDavid Sánchez-MolinaPaweł SzymańskiDavid SedmidubskýPaweł TurekDavid ŠilhaPaweł TwardowskiDavid SinghPaweł WieczorekDavid StratosPawel WrobelDavid VokounPawel WysmulskiDavid W. LawPaweł ZmarzłyDavid W. WheelerPaweł ŻochowskiDavide BarrecaPayam ShafighDavide CastagnettiPayam ZarrintajDavide ClematisPedapati Srinivasa RaoDavide ForcelliniPedram AzariDavide G. SangiovanniPedro Aguilar-ZarateDavide MancinoPedro Contreras LallanaDavide MasatoPedro E. Sanchez-JimenezDavide PorrelliPedro Luis De Hoyos MartínezDavide RoccoPedro M.S.M. RodriguesDavide VurroPedro MarquesDavood FereidooniPedro MartinsDavood KharaghaniPedro Raposeiro Da SilvaDavood RahmatabadiPedro Renato Tavares AvilaDavor GracinPedro ReyesDavor SkejicPedro RoblesDawei WangPedro RomeroDawen ZengPedro Sánchez-SotoDawid JanasPedro SimõesDawid ŁysikPedro SumanDawid ZychPedro Vargas-ChableDaxin LiPedro Y. S. NakasuDaxin LiangPeem NuaklongDay Shin HsuPeihu GaoDayana Cristina Silva GarciaPeijiang LiuDaynier Rolando Delgado SobrinoPei-Li SunDayun YanPeiman AzarsaDebarish MishraPeisheng LiuDebarup DasPeiyang ShiDebashree ChakrabortyPeiyu CaiDebasish Kumar DeyPejman RezaeiDebasish MishraPejo KonjatićDebdulal DasPenchal Reddy MatliDébora FerreiraPeng Chen (China)Débora Lopes Salles ScheffelPeng Chen (Japan)Deep KalitaPeng DuDeepa KodaliPeng GaoDeepak KulkarniPeng HaoDeepak KumarPeng HeDeepak Kumar KhajuriaPeng JiangDeepak Kumar PatelPeng Li (Australia)Deepankar Kumar AshishPeng Li (China)Deepesh UpadrashtaPeng LinDehui XuPeng ShiDejan JeremicPeng TaoDejan MilenkovicPeng WangDekang XuPeng XueDelia PatroiPeng YangDeling YuanPeng YuDelong HePeng Zhang (China)Demei LeePeng Zhang (Harbin Institute Demetrio Jackson Dos SantosPeng Zhang (Jinan University)Demetrios Demetrios GhanotakisPeng Zhang (USA)Demetrios M. CotsovosPeng Zhang (Zhengzhou University)Denilson AguiarPeng ZhaoDenis A. OsinkinPeng ZhengDenis BenasciuttiPengbo WangDenis GokhfeldPengchao KangDenis KarimovPengfei LiuDenis MiroshnichenkoPengfei WangDenis NazarovPengpeng ShiDenis O. AlikinPengyuan XiuDenis OpraPensak JantrawutDenis PankratovPentti NieminenDenis PustovoytovPentti SaarenrinneDenis RodriguePer WennhageDenis RogozhnikovPere BrunaDenis Sergeevich VoroshilovPeriyasami GnanaprakasamDenis VinnikPetar AntovDenisa OlekšákováPetar KassalDenise Antunes Da SilvaPetar T. TodorovDenise BellisarioPete WalkerDenise Crocce Romano EspinosaPeter A. AjibadeDenise-Penelope N. KontoniPeter AntalDeniz AdiguzelPeter BaranDeniz Tanıl YücesoyPeter BukovecDennis DouroumisPeter ĎurčanskýDennis FlanaganPeter E. MurrayDenny CoffettiPeter FinkelDenzel BridgesPeter FutášDeok-kee KimPeter GänglerDeok-Yong ChoPeter HorňákDepei ZhangPeter J. SzabóDer Yuh LinPeter JeglicDerun ZhangPeter JurčiDerya DispinarPeter K. LiawDeshang HanPeter KaššayDesire Molina AlcaidePeter KoledaDesislava StanevaPeter KotešDetlef KlimmPeter LukácsDevaraj VivekPeter MajeričDevarajan BalajiPeter MarotiDevarajan Dinesh KumarPeter MeisenheimerDevasish ChowdhuryPéter NémethDevesh PuneraPeter NiemzDewen HouPeter R. LaityDewi Suriyani Che HalinPeter Rowley-ConwyDeyanira Ángeles-BeltránPeter RusinovDezhen WuPeter ScharffDezhong KongPeter TaylorDezso BekePeter ThissenDezső GergelyPeter TimminsDhanapal PravarthanaPeter TzvetkovDhanasekaran VikramanPéter VancsóDhanasingh Sivalinga VijayanPeter ZarrasDhanya SunilPeter ZollikerDharamdeep JainPethurajan VigneshDharmapura MurthyPetko PetkovDharneedar RavichandranPetr FilipDheeraj K. SinghPetr FormánekDheeraj KumarPetr HaušildDhiraj BhatiaPetr KadlecDhritiman BhattacharyyaPetr KnotekDi ShiPetr KonvalinaDi WanPetr LichýDi WangPetr OpělaDi Wu (Chinese Academy of Sciences)Petr PivkinDi Wu (Guangzhou University)Petr RyapolovDi YunPetr ŠestákDi ZhangPetr ShvetsDiana Balogh-WeiserPetr ŠittnerDiana Camelia NuţǎPetr SmolkaDiana CiolacuPetr SokolovDiana HorkavcováPetr StraumalDiana ManukovskayaPetr SyselDiana Movilla QuesadaPetr V. PrikhodchenkoDiana Petronela Burduhos NergisPetri P. KärenlampiDiane SchorrPetrica VizureanuDianta GintingPetros E. TsakiridisDianyun ZhangPetros KoutsovitisDíaz Real Jesús AdriánPetros MourouzisDibakor BoruahPetros PetrouniasDibya PandaPetru MihaiDibyendu AdakPetru PascutaDichuan ZhangPeyman GholamiDidier R. LongPeyman MehrabiDidier TalamonaPhan Nguyen HuuDiego BarcellosPhilip YuyaDiego GinoPhilipp Del HougneDiego Martinez-MartinezPhilipp KrooßDiego PaschoalPhilipp NiederhoferDiego Said SchicchiPhilipp RöseDiego SebastianiPhilipp SeilerDiego VergaraPhilipp V. Kiryukhantsev-KorneevDieter VollathPhilippe ChristolDigendranath SwainPhilippe LambinDijendra Nath RoyPhilippe LecomteDilip BagalPhilippe TailhadesDilip MishraPhuc Phung-VanDilyana PanevaPichan KaruppasamyDima CheskisPier Carlo RicciDimitar ArnaudovPierantonio De LucaDimitra VernardouPiercarlo RomagnoniDimitrios A. ExarchosPiergiorgio GentileDimitrios A. KoutsourasPiergiorgio TataranniDimitrios BikiarisPierluigi StipaDimitrios GiliopoulosPierre ArnaudDimitrios KalderisPierre MontmitonnetDimitrios KioupisPierre-Yves Collart-DutilleulDimitrios LampakisPierre-Yves TessierDimitrios PapoulisPietro AusielloDimitrios RaptisPietro BartocciDimitrios SklirosPietro FotiDimitris KioupisPietro GaliziaDimitris TsamatsoulisPietro OlivaDimitry A. ChuprakovPietro RussoDimos TriantisPietro TagliatestaDimple DuttaPilar Valles GonzálezDina AbdellatifPilgun OhDina DeynekoPimduen RungsiyakullDina DudinaPing JiangDina FarrakhovaPing LiDina IbrahimPing WangDina V. DudinaPiotr BeerDinara SobolaPiotr BorowskiDinesh C. GuptaPiotr BorysiukDinesh K. AgrawalPiotr DudekDinesh KumarPiotr GarbaczDinesh PanditPiotr GaudenDinesh PathakPiotr GębaraDinesh RokayaPiotr IzakDineshkumar SengottuveluPiotr JeleńDing WangPiotr KamedulskiDing ZhouPiotr KaminskiDingding XiangPiotr KnyziakDinithi PeirisPiotr KoniorczykDino AquilanoPiotr KuryloDiogo Alves GalicoPiotr KuśtrowskiDiogo Duarte Dos ReisPiotr LulińskiDiogo E. V. AndradePiotr MackiewiczDiogo MontalvãoPiotr MalaraDipak RanaPiotr MarcinowskiDipen Kumar RajakPiotr MatczakDipti Ranjan SahuPiotr MazalskiDiptonil BanerjeePiotr MorasiewiczDirk BettgePiotr OsuchDirk H. OrtgiesPiotr OwczarzDirk J. E. M. RoekaertsPiotr Paweł WoyciechowskiDirk PonsPiotr PiszczekDirk RueterPiotr SkubiszDirk SpaltmannPiotr SobotkaDirk UhrlandtPiotr WolszczakDishit ParekhPiotr WróblewskiDitta UngorPiotr WychowańskiDivij BhatiaPiotr ZabierowskiDiwakar KaruppiahPiotr ZachariaszDjalal TrachePiotr ZielinskiDjordje MedarevićPiotr ZimaDjordje VukelicPitchipoo PandianDmitrii Andreevich SinevPiti SukontasukkulDmitrii IrzhakPiyaphong PanpisutDmitrii O. GlushkovPiyush Chandra VermaDmitriy A. BukreevPiyush ChaunsaliDmitriy Aleksandrovich MartyushevPiyush PunethaDmitriy KarpenkovPlamen KatsarovDmitriy MakarovPlamen KirilovDmitriy NesmelovPo-Hsun ChenDmitriy TitovPongsakorn JantaratanaDmitriy V. GunderovPoonam SundriyalDmitriy V. MakarovPoopalasingam SivakumarDmitriy Yu. OzherelkovPooya LahijaniDmitry A. Pshenay-SeverinPosa SudhakaraDmitry B. RadischevPostek EligiuszDmitry BuslovichPothiappan VairaprakashDmitry ChinakhovPourya NorouzzadehDmitry FilippovPoushali DasDmitry G. KvashninPouya BarnoonDmitry I. SharapaPouyan GhabeziDmitry KharitonovPouyan Roodgar SaffariDmitry KultinPouyan Talebizadeh SardariDmitry L. WainsteinPo-Wen ChenDmitry MogilevtsevPrabakaran MayakrishnanDmitry NikolayevPrabhakar SharmaDmitry Olegovich PanovPrabhakaran SubramaniyanDmitry SpasskyPrabhakarn ArunachalamDmitry TagantsevPrabhanjan GiramDmitry TsymbarenkoPrabhat Ranjan PremDmitry V. RoshchupkinPrabir HaldarDmitry WainsteinPradeep BadigerDmitry ZimnyakovPradeep Kumar MishraDmitry ZinoveevPradeep Kumar PandaDo Van ThomPradeep LamichhaneDobrochna Ginter-KramarczykPradeep SambyalDobrota DanPradhumna Mahat ChhetriDoddahalli Hanumantharayudu NagarajuPrakash BhuyarDolendra KarkiPrakash Chandra MishraDolores PereiraPrakash ParthasarathyDolores R. SerranoPramod K. KalambateDomenico CannatàPramod KumarDomenico DalessandriPranesh RoyDomenico LicursiPranesh SenguptaDomenico MontemurroPranitha SankarDomenico RussoPranjal SaikiaDomenico ScaramozzinoPranjali NaikDomenico VizzariPranut PotiyarajDominic ZerullaPrasana Sahoo (Jadavpur University,)Dominika MadejPrasanta Sahoo (Indian Institute of Technology Kharagpur)Dominika SiwiecPrasenjit MaityDominique De CaroPrashant ShrivastavaDominique ThierryPrashanth Konda GokuldossDonald RadfordPrasit ThongbaiDonata Konopacka-ŁyskawaPrathan BuranasiriDonatella DuraccioPratibha RaniDong GuoPratik JoshiDong Joo KimPratiksha DongareDong Joo LeePravin Dinkar NemadeDong MengPravin IngoleDong ShaoPrayoonsak PluengphonDong WangPredrag DašićDong Won JungPredrag PetrovićDong WuPreeti KumariDong Xu WenPrem Ballabh BishtDong Yih LinPřemysl BeranDongdong GePřemysl PařenicaDongdong HanPremysl StastnyDongfeng HePriputen PavolDonghui YangPriya MurugasenDonghyun KimPriyadarshini JayashreeDong-Jun KwonPriyaranjan SamalDonglai ZhongPriyatham TumurugotiDongsheng HuangProbal BasuDongshi ZhangProkhorov Vyacheslav M.DongWon JungPromod KumarDong-Wook HanPromoppatum PatcharapitDongyue XiePruet KowitwarangkulDongzhu LuPrvan Kumar KatiyarDoojin LeePrzemyslaw BartczakDora A. Cortés-HernándezPrzemysław BrzyskiDora FotiPrzemysław BuczyńskiDoreswamy DeepakPrzemysław GłowackiDoretta CapsoniPrzemysław GolewskiDorian HanaorPrzemyslaw LedwonDorin LucaPrzemysław PączkowskiDorin RaduPrzemysław PodulkaDorina Nicolina IsopescuPrzemysław PostawaDorota Czarnecka-KomorowskaPrzemysław StrzeleckiDorota DukarskaPshtiwan ShakorDorota KołodyńskaPt Dmytrakh IgorDorota NeugebauerPu WangDorota PawlusPui Vun ChaiDorota Rutkowska-ZbikPulak Chandra DebnathDorota SiutaPunam MurkuteDorota WęgłowskaPundikala VeereshaDorothea WisserPuneet BatraDoru StanescuPura Alfonso AbellaDouglas E. SmithPurcar VioletaDouglas Vieira ThomazPurushothaman KuppanDovan ThomPushpendra Kumar Singh RathoreDragan D. MilašinovićPuskaraj D. SonawwanayDragan GovedaricaPuthanveettil Madathil AbhilashDragan RodicQammer ZaibDragana D. Vasić AnićijevićQasim Shaukat KhanDragana GabrićQi CaoDragana Vasic AnicijevicQi LiuDragana VasiljevićQi ShaoDragos AchiţeiQi WangDragos-Victor AnghelQi YuDražan JozićQi ZhuDror MalkaQian WangDu FengshanQiang GaoDu Nguyen VanQiang GuoDuangjai TungmunnithumQiang HuangDuarte MarquesQiang Li (HuazhongDubravka MilovanovicQiang Li (Xi’an Jiaotong University)Dubravka Negovetić-VranićQiang ShiDubravko RogaleQiang WeiDuc Duy HoQiang XuDuc The NgoQiang YangDujian ZouQiang YueDulal SahaQiang ZhuDulce BeloQianwen LiuDumith JayathilakaQicheng FengDumitru Doru Burduhos NergisQihan LiuDumitru MitricaQilin GuoDung Nguyen TrongQin ZhangDunja Šajn GorjancQing HaoDunlu SunQing LuDuo LiQing LuoDuong NguyenQing MiaoDuraisamy SathyaQingbin ZhengDurbar RayQingfeng GuoDušan ArsićQingfeng WangDusan RackoQinghua WangDušan VopálkaQinglin LiDuško DudićQingqing KeDyi Cheng ChenQingshan DongDylan AnstineQingshan YangE. R. MéndezQingsheng WuEberhard BurkelQingshun DongEbrahim A. MahdyQingsong YuEbrahim SeidiQinguo FanEbrahim Sharifi TeshniziQingzhi YanEbrahim TabanQinqin HanEbrahimi MehdiQiongyao PengEcaterina Stela DraganQiubao OuyangEdappadikkunnummal ShijuQiucheng LiEder Carlos Ferreira De SouzaQiuliang HuangEdgar FelizardoQiwen QiuEdgar Franco UrquizaQixiang FengEdgar MosqueraQiyang TanEdgars ElstsQiyao HuangEdilso RegueraQiyuan HeEdilson León Moreno CárdenasQizhou CaiEdina RusenQuan ShanEdison Puig MaldonadoQuang PhamEdit XhajankaQuang TranEdmar Oliveira-FilhoQuang-Viet VuEdmilson CorrêaQuanshun LuoEdna PossanQuirino EstradaEdoardo BocciQuoc-Hung PhanEdouard Rivière-LorphèvreR. C. MorónEdson CostaR. PramodEdson Estrada-ArriagaR. Prasath BabuEdson Jansen Pedrosa de Miranda Jr.R. SureshEdson SantanaRabbani SyedEdson Silva FilhoRabia AyubEduard AgeevRabia IkramEduard MuslimovRabia Sannam KhanEduard SosninRabiul IslamEduard-Marius CraciunRachid HakkouEduard-Marius LungulescuRachid HsissouEduardo A. Lopez-MaldonadoRachid TouzaniEduardo BorieRachmat Adhi WIbowoEduardo E. MiróRadanović Mirjana M.Eduardo Garcia BreijoRade VignjevicEduardo JuncaRadhwan AlzeebareeEduardo MolinaRadi JishiEduardo Moreira Da SilvaRadian PopescuEduardo Natividade-JesusRadim HalamaEduardo P. NóbregaRadmila Jančić HeinemannEduardo Valencia MoralesRadmila TomovskaEdward LesterRadosław MirskiEdward RenRadosław PatykEdward SachetRadostina A. AngelovaEdwin-Joffrey CourtialRadouane ZitouneEdyta KardasRadovan BurešEdyta KucharskaRadovan CernyEdyta Osuch-SłomkaRadu C. RacovitaEdyta SpychałRadu FecheteEffendi Tri BahtiarRadu MunteanEfstathios E. TheotokoglouRadu Robert PiticescuEfstratios I. KamitsosRadu-Eugen BreazEfthimios BalomenosRadu-Ioachim ComaneciEftihia BarnesRaees SwatiEgemen AvcuRafael ComesañaEgor VerbitskiyRafael Coutinho Mello MachadoEhab AbdelhieeRafael CuestaEhab AlShamailehRafael Das ChagasEhsan FarabiRafael FontEhsan GhassemaliRafael KakitaniEhsan KhademiRafael M. SantosEhsan NikbakhtRafael Rodolfo De MeloEhsan Noroozinejad FarsangiRafael ShehuEhsan ZeimaranRafael Torres-MendietaEhtasham MustafaRafael Vargas-BernalEiji NishiboriRafael ZadorosnyEijiro MaedaRafał BielasEilon FaranRafał BoguckiEjaz HussainRafal ChodunEkarat MeechoowasRafał GołębskiEkaterina A. SelivanovaRafał K. WyczółkowskiEkaterina D. PolitovaRafal KowerdziejEkaterina KomarovaRafal KozdrachEkaterina KopetsRafał KudelskiEkaterina L. TrukhanovaRafał MechEkaterina MarchenkoRafał MichalczykEkaterina N. StepanovaRafal RakoczyEkaterina PotapovaRafał SzukiewiczEkaterina S. LoktevaRaffaele CioffiEkaterina S. OkhotnikovaRaffaele SepeEkaterina V. KunitsynaRaffaele ZinnoEkkarut ViyanitRaffaella SignoriniEkrem KalkanRaffaella StrianiEl Sayed M. SherifRaffaello MazzaroElad PrielRaffaello PapadakisElaheh BeyabanakiRafiza Abdul RazakElaheh Dalir AbdolahiniaRagab El SehiemyElaheh JooybarRagai MattaElango NatarajanRaghu EchempatiElayappan VijayakumarRaghvendra Pratap SinghElayaraja AruchunanRahim Abdur (Pakistan)Elbaz I. AbouelmagdRahim Abdur (Republic of Korea)Eldon RajRahul BiswasEleana KontonasakiRahul Kumar (Estonia)Eleazar Salinas‐RodríguezRahul Kumar (India)Eleftherios AnastasiouRahul ManeElena Alfredovna YatsenkoRahul MitraElena AnashkinaRahul RamachandranElena Cerro-PradaRahul SharmaElena De CosRahul ShevateElena DilonardoRaimonds MeijaElena EfremenkoRainer HipplerElena FerrettiRaj AryaElena FilonovaRaj KarthikElena FuenteRaj Kumar AryaElena G. VolkovaRaj Kumar SahuElena GarleaRajaboopathi ManiElena KleninaRajalekshmi C. PillaiElena KorznikovaRajamani DevarajElena López-MayaRajamani NagarajanElena M. Peña-VázquezRajan ChoudharyElena ManailaRajan KrishnaprasadElena MarrocchinoRajan Kumar SinghElena Martinez-SanzRajani VijayaraghavanElena MicheliniRajarathinam VadivelElena N. KorostelevaRajavaram RamaraghavuluElena O. NasakinaRajeev KumarElena OrlenkoRajeev VermaElena PolisadovaRajender BoddulaElena PopovaRajendran SelvakesavanElena RomeoRajesh HaldharElena SalernitanoRajesh Jesudoss Hynes N.Elena ShekaRajesh KaluriElena StoccoRajesh Kumar MishraElena StraumalRajesh MishraElena Yu KramarenkoRajesh PaulrajElena ZhitovaRajesh V. NairEleonora ConterositoRajesh YadavEleonora PargolettiRajeshkumar SelvarajEleonora SantecchiaRajib ChowdhuryEl-Eswed BassamRajib KalsarElhassan AmaterzRajib Kumar MajhiEli GrigorovaRajib PaulElia Mercedes Alonso GuzmánRajib SahuElias Ferreiro VilaRajini NagarajanElias SakellisRajiv Ranjan SrivastavaElif TopalogluRajkumar P. ThummerElif YildizRajkumar VeluEligiusz PostekRajni GargElisa CantergianiRaju Kumar GuptaElisa FracchiaRaju M.V.A. BahubalendruniElisabete R. TeixeiraRajveer JhaElisabetta FinocchioRajwali KhanElisabetta PolizziRajyalakshmi GajjelaElisan Dos Santos MagalhãesRakesh BhaskarElizabeth A. KoulaouzidouRakesh KumarElizaveta P. SimonenkoRakesh RajaboinaEllen FunkhouserRakhi TiwariElliot WainwrightRalf HofmannElmar TräbertRalf PudeElmira SaljnikovRalph BäßlerElmira VelayiRalph PuchtaElnaz EsmizadehRaluca Florentina NegreaEloisa SardellaRaluca IsopescuEloy CondeRam KarthikeyanElsa FonsecaRama Krishna AllaElsayed ZakiRama S. KomaragiriElsisi AlaaeldinRama Sreekanth Pattela SrinivasaElson LongoRamachandra NaikElton Ray BrownRamachandran Chidambaram SeshadriElvio CarlinoRamadan SalimElvira OñorbeRamadhansyah Putra JayaEly ValbuenaRamaiah PrakashElyas Asadi ShamsabadiRamalingam GopalElza Maria Morais FonsecaRamamoorthi RangasamyElzbieta Grza̧dkaRamamoorthy KumarElżbieta HorszczarukRaman Kumar (Chandigarh University)Elżbieta JartychRaman Kumar (Guru NanakElżbieta Radziszewska-ZielinaRamanahalli Jayadevamurthy PunithgowdaElżbieta SzymszykRamanathan ThirumalaiElz̀bieta TomaszewiczRamanuj KumarElżbieta TrafnyRamanujam SarathiEmad A. AbraikRamanujan GovindarajEmad Abdul-Hussain YousifRamaraghavulu RajavaramEmad ScharifiRamaraj KannanEman MwafyRamasamy BhoopathiEmanuel CarlosRambabu YalavarthiEmanuela SperanziniRamdayal YadavEmanuele CavaliereRamesh Gupta BurelaEmanuele GalvanettoRamesh KanthasamyEmanuele VIncenzo ArcieriRamesh KumarEmerson CoyRamesh Kumar NayakEmerson F. FelixRamesh MandaEmil BabícRamesh RudrapatiEmil Engelund ThybringRamesh SinghEmil EvinRamez A. Al-MansobEmil PetrescuRamez HajjEmil SasimowskiRami K. SuleimanEmília HroncováRami MhannaEmilia IrzmańskaRamin EbrahimiEmilia V. SillettaRamin HashemiEmilian MosnegutuRamin RahmaniEmiliano ZampettiRamin Rahmani AhranjaniEmilio BassiniRamin RaiszadehEmilio Garcia TaenguaRamin YousefiEmilio LuengoRamkumar ThulasiramEmiliy ZholkovskiyRamoel SerafiniEmin BayraktarRamón Alfonso Iñiguez PalomaresEmir BuzaRamón Corral HigueraEmishaw IffaRamón González RubioEmmanouil Lazaros PapazoglouRamon PamiesEmmanuel Fortunato GulinoRamón Raudel Peña-GarciaEmmanuel KeitaRamón Torres-CavanillasEmmanuel MarinRamona CimpoeşuEmmanuel P. GeorgiouRamona KuhnEmmanuel Segura-CárdenasRamune ZurauskieneEmmanuel TopoglidisRamya ShenoyEmrah MadenciRan SuckeverieneEmre CinkilicRan TianEmre OcakRana Faisal TufailEnache StanicaRana Muhammad Arif KhalilEneli HaerkRanajoy BhattacharyaEner SemihRanda Abdel SalamEngin BurgazRandeep SinghEngin TırasRangasayee KannanEnikö GyörgyRanhua XiongEniko T. EnikovRania A.H. IshakEnkhnaran UyangaRanjan MishraEnobong Reginald EssienRanjit DeEnrico AndreoliRanjith Kumar Deivasigamani Enrico BernardoRapeepan PitakasoEnrico BerrettiRaphael DeltombeEnrico DestefanisRaphael JanotEnrico GherloneRaphael PfattnerEnrico LemmaRaquel De Melo BarbosaEnrico M. CamporesiRaquel Fernandez-GarciaEnrico SalvatiRasa MladenovicEnrico TroianiRasha AwniEnrique CasarejosRashid G. BikbaevEnrique Domínguez-ÁlvarezRashid K. Abu Al-RubEnrique Fernandez LedesmaRashid KhanEnrique Rocha-RangelRashid NazirEnriqueta AnticóRashidah ArsatEnshirah Da’naRashmi NayakEphesus Olusoji Ephesus Olusoji FatunmbiRasim AlosmanovErcan IsikRasmus TalvisteErdinç ÖzRastko VasilićEremina Galina M.Ratan DasEren ŞahinerRatchagaraja DhairiyasamyErfan MalekiRatchatee TechapiesancharoenkijErfan RezvaniRathapon AsasutjaritErgo RikmannRathinavelu SokkalingamEri Miura FujiwaraRatna EdiatiEric A PattersonRatnakar DasÉric BousquetRatnakumar V. BuggaEric FujiwaraRattana JariyaboonEric J. StantonRauf ForoutanEric Kwok Wai TamRaul ArrabalEric QuarezRaul Carrillo-PedrozaEric W. NeumanRaúl Cazorla-LunaErich RodriguezRaul D.S.G. CampilhoErick Juarez-ArellanoRaul Rosales-IbáñezErik BirksRaúl Tauste MartínezErik PiattiRavi KantÉrika C. AlvarengaRavi Kumar CheedaralaErika García-LópezRavi PandiselvamErjin ZhengRavi TutikaErkan IlikRavinder KumarErkan ÖzkanRavindra D. JilteErkan SensesRavindra K. SaxenaErkut YalcinRavindran SujithErlin ZhangRayner GeorginaErnesto AusilioRayya A. Al-BalushiErnesto Beltrán-PartidaRaz MuhammadErnesto Chigo-AnotaRazieh RazaviErnesto PlacidiRazi-ul HaqueErnesto ReverchonRaziyeh AkbariErnests PlatacisRazvan Ioan PacurarErol YildirimRazvan UdroiuErsan HarputluRebeca Martínez-GarcíaErshuai ZhangRebeka RudolfErsilia GiordanoRecep GüneşErsin KayahanReda El-ShishtawyErvin RáczReda SalamaErwin WojtczakReddy NarendraEryk WolarzReddy Ramireddy ThrinathEsam Abdel HadyReem AbualsaudEsam Bashir YahyaReem HannaEsam YahyaRegina Fuchs-GodecEsequiel MesquitaRegina PutsEsfandiar PakdelRegis BarilleEsmaeel SharifiRégis Henrique Gonçalves E SilvaEsmaeil Doustkhah HeraghRegita BendikieneEsmaeil HeydariRehan UmerEsmat HamzawyReijo KouhiaEsperanza DiazReinaldo Rodríguez RamosEssam AhmedRéka BarabásEssam B. MoustafaRemya P. NarayananEssam MoustafaRenan Dal-FabbroEssam R.I. MahmoudRenan FalcioniEssam WahabRenan TrevisoliEssia HannachiRenata BorisEstaner RomãoRenata DwornickaEster Gimenez-CarboRenata ŁyszczekEsther J. OcolaRenata OdžakEsther RebollarRenata Salerno-KochanEszter BódisRenata WłodarczykEugen AxinteRenato GonçalvesEugen GheorghiuRenato P. De LimaEugene A. KotominRenaud RinaldiEugenia Eftimie TotuRendi KurniawanEugenia Fagadar-CosmaRendy ThamrinEugenia TotuRené PyszkoEugenija StrazdienėRengaraj SelvarajEugenio Velasco OrtegaRen-Jang WuEugeniusz KodaRen-Jei ChungEui Soo KimRenjie LiuEun Bin BaeRenjith ThomasEun Ik YangRen-Kae ShiueEunho LeeRenzo GuarnieriEunice CarrilhoReshma SonkusareEunsongyi LeeRethinam SenthilEurig Wyn JonesRevannath Dnyandeo NikamEva BartonickovaReyes Montero ArmandoEva CuninkováReynold E. SilberEva Epelde BejeranoReza AlizadehEva MartinsReza ArjmandiEva Oktavia NingrumReza BagheriEva OllerReza BeygiEvaggelos KaselourisReza Faghih MirzaeeEvaldo José CoratReza GhiaasiaanEvangelia SarantopoulouReza ImaninasabEvangelia TaraniReza KolahchiEvangelia VouvoudiReza MolaeiEvangelos AlmpanisReza PeymanfarEvangelos PetrakisReza SalehiyanEvelina P. DomashevskayaReza TaghiabadiEvelyn TaboadaReza TaherdangkooEverardo Granda-GutiérrezReza VaghefiEverson Thiago Santos Gerôncio SilvaRezgar HasanzadehEvgenia Korzhikova-VlakhRezvan BabagoliEvgenii I. MareevRi ZhangEvgenii KuzinRiadh NeffatiEvgenii M. Shcherban’Riaz MuhammadEvgenii RoginskiiRicardo Andrés García-LeónEvgenii VasilevRicardo Borda Lopes VieiraEvgeniia GilshteinRicardo BrancoEvgeniia VikulovaRicardo CarbasEvgeniy ChistyakovRicardo CastedoEvgeniy S. TkachevRicardo Castro AlvesEvgeniy Vladimirovich KislovRicardo Domínguez-ReyesEvgeniya PankratovaRicardo Faria AlmeidaEvgeny A. TrofimovRicardo J. Alves De SousaEvgeny FilatovRicardo J. FernandesEvgeny KarpushkinRicardo M. F. FernandesEvgeny KravtsovRicardo MendesEvgeny M. ApfelbaumRicardo OrozcoEvgeny MikhailovRicardo SchneiderEvgeny ParfenovRicardo VardascaEvgeny PrusovRiccardo BeltramiEvgeny ShermanRiccardo BiondiEvgeny Viktorovich ShilkoRiccardo CerulliEwa DryzekRiccardo Di CoratoEwa GlowinskaRiccardo Di GianfilippoEwa GorodkiewiczRiccardo MonterubbianesiEwa GrzankaRiccardo NifosìEwa JondaRiccardo PaoliniEwa Juszyńska-GałązkaRiccardo RossiEwa OledzkaRiccardo SalvioEwa StodolakRiccardo VescoviniEwelina CichońRichard CannonEyad HamadRichard ClergereauxEyo Umo EyoRichard DrevetEzequiel Caires Pereira PessoaRichard J SheridanF. Taheri BehroozRichard J. CurryFabian DuvigneauRichard KlemmFabian MederRichard PastirčákFabian StarsichRichard PinčákFabiana Pereira Da CostaRichard SheardyFabiano S. RodembuschRichard Thomas LermenFabien BenedicRichard Torrealba-MelendezFabio Antonio XavierRichard WierichsFabio CarniatoRickard OlssonFábio Filipe Fereira GarrudoRico Victor J.Fabio FratiniRida EssajaiFabio GiudiceRidvan YamanogluFabio MianiRihab HadjiFabio PalumboRiham M. FliefelFabio SattinRijuta GaneshFabio SimõesRimantas KačianauskasFabio ZobiRimas JaneliukstisFabiola Monroy GuzmanRinaldo Dos Santos AraújoFabiola PinedaRintaro UejiFabrice OehlerRisdiana RisdianaFabrice SthalRita Bacelar FigueiraFabricio Maestá BezerraRita EspositoFabrizio CardoneRita NogueiraFabrizio FioriRita Patrizia AquinoFabrizio GabrielliRitamay BhuniaFabrizio MarraRitesh Kant GuptaFacundo Almeraya-CalderónRitesh Ray ChaudhuriFacundo RuizRitesh VermaFadis F. MurzakhanovRitu RavalFadzli Mohamed NazriRityuj Singh PariharFaezeh ShanehsazzadehRıza Görkem OskayFahad A. AlhumaydhiRizwan Rahman RashidFahad AlamRizwan WahabFahad K. AlqahtaniRobert BidulskýFahad MateenRobert BirchFahad UsmanRobert CackoFaham TahmasebiniaRobert CarleerFahanwi Asabuwa NgwabebhohRobert Catalin CiocoiuFahe CaoRóbert FindorákFaheemuddin PatelRobert GeorgiiFahim AhmedRobert JurczakFahimeh TabatabaeiRóbert KočiškoFahmi F. MuhammadsharifRobert KonowrockiFahmi ZairiRobert KosturekFaisal QayyumRobert KralFaissal AzizRobert M. HousleyFaiz ArithRobert MroczyńskiFaiz MuhaffelRobert OlivaFakhroddin NazariRobert OssendorffFalk WittelRobert PealeFamulari AntoninoRobert PelkaFan YangRobert PeterFang ChengRobert PrzekopFang HaixingRobert Saraiva MatosFang LiuRobert StarostaFangping ZhuoRobert TournierFani StergioudiRobert UlewiczFania PalanoRoberta BertaniFan-Jiang MengRoberta GasparroFanny Monteil-RiveraRoberta LicheriFanqiang MengRoberta Maia SabinoFarah Asa’adRoberta MassabòFaraj H. TobiaRoberta OkamotoFaraz SoltaniRoberto AguadoFaraz Ui HaqRoberto AvolioFarhad HuseynovRoberto CesareoFarhad ImaniRoberto ChristFarhad OstovanRoberto DÁmatoFarhad Rezai-AriaRoberto De SantisFariba MollarasouliRoberto DovesiFariborz FarajiRoberto EggenhöffnerFarid El-AskaryRoberto FiorenzaFarid OrudzhevRoberto GarcíaFaridah KhairuddinRoberto Li VotiFaris TarlochanRoberto Lo GiudiceFarnaz BatoolRoberto MeneghelloFarrukh HafeezRoberto MontanariFarrukh MukhamedovRoberto PalazzettiFarshid AramRoberto PetrucciFarshid PahlevaniRoberto RellaFarshid SefatRoberto Ruggiero BragaFarzad PourfattahRoberto RussoFarzin GhadamiRoberto SalcedoFateh BouchaalaRoberto SañudoFateh Mebarek OudinaRoberto ScaffaroFatema H. RajabRoberto TeghilFatemeh MokhtariRoberto TermineFatemeh S. HosseiniRoberto Vazquez-MunozFatemeh ShahriyariRoberto VerucchiFathoni UsmanRoberto ZivieriFatih AydinRoberts EglitisFatih KarpatRobin TurnbullFatih SelimefendigilRocco CancelliereFatih YilmazRocco Di GirolamoFátima De Cássia Oliveira GomesRoderick GuthrieFatma Nur ParınRoderik PlavecFaustino Aguilera-GranjaRodica ZavoianuFaustino MujikaRodica-Mariana IonFausto FiorilloRodolfo RedaFausto TucciRodolpho Fernando VazFawad KhanRodrigo De Almeida SilvaFawzy Hosny SamuelRodrigo FrançaFayaz HussainRodrigo Henrique GeraldoFayçal DjeffalRodrigo ResendeFazal Ur RehmanRodrigo SalvadorFederica Di SpiritoRodrigo Sávio PessoaFederico AnticoRodrigo V. SilvaFederico BellaRoer Eka PawinantoFederico BertonRoger M. RowellFederico GuarracinoRogerio ColacoFederico MazzucatoRogério Leone BuchaimFederico MontoncelloRogério RibeiroFederico PicolloRogerio Sousa JuniorFedor GrigorievRoghayeh MohammdzadehFedor ShakhovRoham RafieeFeezan AhmadRohan SomanFehim FindikRohana AhmadFei FanRohit YadavFei LiRohollah NasiriFei LiuRok FinkFei LyuRok GaspersicFei ShaRoland K. ChenFei Yi HungRoland SchochFei YuRolandas TomasiunasFeifei ZhangRolandas UrbonasFeifei ZhouRolando PediciniFeipeng YangRolands CepuritisFeiting ShiRolf SandströmFeldshtein EugeneRomain MouryFelice FemianoRoman A. IrgashevFelice RubinoRoman A. SurmenevFelicia GheorghiuRoman A. ZakoldaevFelicia Ramona BirauRoman ChertovskihFeliks StachowiczRoman GrillFelipe Dos Santos LoureiroRoman HeubergerFelipe FantuzziRoman KolenakFelipe Garcia-SanchezRoman KulaginFelipe L. Penaranda-FoixRoman MoravčíkFelix DonatRoman PrzyłuckiFelix PalumboRoman PuzniakFen XueRoman RéhFeng ChenRoman SavraiFeng HeRoman ShendrikFeng JiangRoman StarostaFeng ZhaoRoman SurmenevFenghe WuRoman SvetogorovFengmiao LiRoman SzewczykFengqi GuoRoman TurczynFengshan ZhouRomana MikšováFengxia DengRomeo CiobanuFengyuan LiuRomeu ChelariuFeras AlasaliRomina ConteFerdi CihangirRomisuhani AhmadFerdinando CostantinoRomuald RządkowskiFerenc GillemotRomualdas TrusovasFermín BañónRonald FallerFernanda Campos SousaRonald M. BarrettFernanda Da Cruz Landim- AlvarengaRonaldas JakubovskisFernanda Guerra Lima Medeiros BorsagliRonaldo CozzaFernando C. G. MartinhoRongchuang ChenFernando CalzadaRongfu Rongfu WenFernando CastroRong-Ho LeeFernando ElyRongrong CheacharoenFernando G. BrancoRongxin GuoFernando GomesRonn GoeiFernando J. PereiraRoobanvenkatesh ThirumalaiFernando JulianRoong Jien WongFernando LloretRoope HusgafvelFernando Lopez GayarreRoozbeh Abedini NassabFernando Moura DuarteRory WatermanFernando RochaRosa Di MaggioFernando SimõesRosa Garcia SalvadorFernando SuárezRosa Lo FranoFernando Veiga-SuárezRosa RodriguezFernão D. MagalhãesRosa VeropalumboFeruza A. AmirkulovaRosalía PoyatoFethi AbbassiRosalío Fernando Rodríguez ZepedaFigen BaloRosalynn NankyaFilip GucmannRosanna MabiliaFilipe Hobi Bordón SosaRosaria Anna PiccaFilippo GiannazzoRosario Garcia GimenezFilippo MarchelliRosario MontuoriFilippo ParisiRosario NuñezFilippo RenòRosarita D’OrsiFilippo RomanatoRosen MitrevFiona SammlerRosenda Valdés ArencibiaFioretta AsaroRoshan Thotagamuge KumaraFirman Mangasa SimanjuntakRosita DianaFiroz Babu KadumudiRositza DimitrovaFiroz KhanRosolino VaianaFitria RahmawatiRossana IzzettiFlavia IaculliRossella ArrigoFlavien ValensiRossella SvigeljFlávio Alberto Da Silva FigueiraRossen TodorovFlavio BeneduceRostislav ApraksinFlávio ColmatiRoto RotoFlávio M. VichiRouzbeh GhabchiFlavio StochinoRoxana MunteanFlora FaleschiniRoxana RacoviceanuFlorentina GolgoviciRoxana RuseckaiteFlorian BittnerRoxana VasiliuFlorian MartoïaRoxanne RadpourFlorian PabstRoy Alvin MalenabFlorian PapeRoy Byung Kyu ChungFlorian PuchRoy SanjayFlorian SpieckermannRu JiFlorian WelzelRuan L. S. FerreiraFlorica Imre-LucaciRuangdaj TongsriFlorin MiculescuRubén Abraham Domínguez-PérezFlorin OanceaRubén Del OlmoFlorin PopaRuben DoradoFlorina BranzoiRubén Gonzalez NunezFlorina TeodorescuRuben HühneFolker WittmannRúben SantosFong-Yu ChengRubens Nisie TangoFoo Wah LowRuchi BhartiFouzia KhadraouiRuchir PriyadarshiFowzia AkhterRudolf HolzeFranc ZupaničRudolf KieferFrancesca AccioniRuey-Ching TwuFrancesca CegliaRuham Pablo ReisFrancesca FerrariRui BordaloFrancesca LeonardiRui C. MartinsFrancesca MaltintiRui ChenFrancesca NerilliRui DaiFrancesca ZottiRui F. M. LoboFrancesco ArmettaRui Feng KanFrancesco BarrecaRui HuangFrancesco BennardoRui M. B. CarrilhoFrancesco BrandaRui MarquesFrancesco BuonocoreRui MinFrancesco CaddeoRui P. P. L. RibeiroFrancesco CapitelliRui PereiraFrancesco CarboneRui SilvaFrancesco CaridiRui VilarFrancesco CiardelliRui XiaoFrancesco ColangeloRuihua DongFrancesco CorvaroRuijie XuFrancesco D’AmicoRuijing LiangFrancesco FabbrocinoRuiliang LiuFrancesco FusoRuitao LiFrancesco GhezziRuixiang BaiFrancesco LiguoriRuizhe SiFrancesco Marotti De SciarraRumana HossainFrancesco ModicaRuming PanFrancesco MontalentiRun-Sheng LinFrancesco MoscatelliRuoyu HanFrancesco NobiliRupanjali PrasadFrancesco PotenzaRupert TscheliessingFrancesco PuosiRusănescu Carmen OtiliaFrancesco RossellaRushdan A. IlyasFrancesco Saverio LudovichettiRuslan BarkovFrancesco TornabeneRuslan IbragimovFrancesco VenialiRuslan K. VagapovFrancesco ZirilliRuslan MelentievFrancis BretonRuslan V. FedorovFrancis ChipemRuslan ValievFrancis J. OsongaRut BenaventeFrancisca Arán AisRutger MatthesFrancisco AgrelaRuzica NikolicFrancisco AlguacilRužica R. NikolićFrancisco C. Franco Jr.Ryan LeeFrancisco CastellanosRyan MorrowFrancisco CuevasRyosuke KomodaFrancisco De AzambujaRyouichi SatouFrancisco Eroni Paz Dos SantosRyoya IshigamiFrancisco GilabertRyszard PawlakFrancisco J.A. LoureiroRyszard ProrokFrancisco Javier AyasoRyuta IchikiFrancisco Javier BelzunceS. Hashem Mousavi AnijdanFrancisco Javier BolivarS. KandasamyFrancisco Javier López-TenlladoS. M. Hossein SeyedkashiFrancisco Javier Muñoz SaezS. MadhuFrancisco Javier NavarroS. MarichamyFrancisco Javier Rodríguez LozanoS. P. JaniFrancisco Lopez-LinaresS. R. MahmoudFrancisco Montero-ChacónS. RamalingamFrancisco MoreaS. Renold ElsenFrancisco PasadasS. S. Rahimian KoloorFrancisco RescalvoS. S. VermaFrancisco Rey GarcíaS. V. Prabhakar VattikutiFrancisco SilvaS. VigneshwaranFrancisco Solis PomarS.R.R. Teja PrathipatiFrancisco Tovar LopezSaba GharehdashFrancisco Werley Cipriano FariasSaba MosivandFrancisley Ávila SouzaSabapathy ThennarasanFranco LombardiSabata MartinoFranco ZanottoSabbah AtayaFrançois DelattreSabina AbbrentFrancois Le NormandSabina KramaFrancois RaultSabina NilufarFrançois RopitalSabine ValangeFrançois VidalSabrina A. CamachoFrank BalzerSabrina RoguaiFrank BelloSabyasachi ChakraborttyFrank DaschnerSabyasachi GhoshFrank E. GoodwinSabyasachi KarFrank KernSabyasachi MukhopadhyayFrank P. MissellSachchida Nand RaiFrantisek ChmelikSachin SalunkheFrantišek KrčmaSachin SirohiFranz DemmelSadaf Bashir KhanFranziska HoffmannSadegh KarimpouliFranziska SchmidtSadhasivam ThangarasuFrazer BrownlieSadia IlyasFrédéric DumurSadia Zafar BajwaFrederic HeimSadık Alper YıldızelFrederico PenhaSadik Ozgur DegertekinFrederico RodriguesSadok MehrezFrederieke LangerSaeed Ahmad QaisraniFrederik ZangerSaeed Ahmed KhanFrédérique DucroquetSaeed AmirFredrick MwemaSaeed AshtianiFredrik SikströmSaeed Ghaffarpour JahromiFriedrich WaagSaeed HasaniFuchuan DuanSaeed Khani MoghanakiFulai ZhaoSaeed MohammadzadehFulga TanasăSaeed MozaffariFulgencio AlatorreSaeed OlyaeeFulin JiangSaeed Saber-SamandariFulong ZhuSaeed YousefinejadFumin RenSaeid KargozarFumio OgawaSaeko Tada-OikawaFumitada IguchiSafia AkramFumitaka HayashiSagar ManeFupin LiuSagar NikamFuping YuanSagrario Martínez RamirezFuqiang TianSahand SarbisheiFuren XiaoSahar MollazadehFurong CaoSaheed A. TijaniFurqan AhmedSahu Bibhu PrasadFuyao YanSai Aditya PradeepG. H. M. J. Subashi De SilvaSai Krishna PadamataG. LogeshSai Ramudu MekaG. Mohan GaneshSai Sathish RamamurthyG.R. Mohtashsami BorzadanSaïd BouabdallahGabin GbabodeSaid LaasriGabor CameliaSaid RideneGábor GyarmatiSaif AlzabeebeeGábor KozmaSaiful IslamGábor VértesySaikat MaitiGabriel AbabeiSaikat Ranjan MaityGabriel Alejandro LopezSait Barış GünerGabriel AullonSajad SohrabiGabriel FedorkoSajid Ali AnsariGabriel K. P. BarriosSajid SaleemGabriel Lucian RaduSajjad HussainGabriel MansourSajjad MalikGabriel OprisanSajjan DahiyaGabriel ProdanSakdirat KaewunruenGabriel SaliernoSakin H. JabarovGabriel VasilieviciSakineh ChabiGabriel WittenbergerSakineh FotouhiGabriela BuemaSakon RahongGabriela ChiritoiuSakshum KhannaGabriela DudekSakthivel GnansekaranGabriela MǎrgineanSalah Aldin FaroughiGabriela Romero-UribeSalah BoulaarasGabriela RosaSalah JellaliGabriela RutkowskaSalah Mohamed El-BahyGabriela ToaderSalah Ud-Din KhanGabriela VinczeSalas Vicente FidelGabriele CervinoSalawu SulymanGabriele GiancaneSalber JochenGabriele MilaniSalem Al-AmeriGabriele MulasSalh AlhammadiGabriella CsíkSalih DurduGabriella SzabóSalim AlbukhatyGaddam RajeshkhannaSalim BarbhuiyaGaëtan BuvatSalim HizirogluGaetano PalumboSallal R. AbidGaetano PaoloneSally ElasheryGagan KumarSalmabanu LuharGagik GurzadyanSalman Ebrahimi-NejadGaili XueSalman Khalid (Pakistan)Gajendra InwatiSalman Khalid (Republic of Korea)Galal FaresSalman ShooshtarianGalder CortaberriaSalmon LandiGalina A. DavidovaSalvador BuenoGalina AbrosimovaSalvatore AlfonzettiGalina KurlyandskayaSalvatore BocchieriGalina MelnikovaSalvatore GalloGaliveeti Hemakumar ReddySalvatore PatanèGanapathy S. SivakumarSalvatore PernaGanchai TanapornraweekitSalvatore ScaccoGanesh IngavleSalvatore VerreGang GeSam LoflandGang Seob JungSamad M. E. SepasgozarGang SongSaman GhahriGang XuSaman IlankoonGanna StovpchenkoSamaneh Salkhi KhasraghiGao LiSamantha M.S.B. DissanayakaGaochao YuSamarendra MajiGaofeng ZhengSambandam AnandanGaohui WuSambhunath BeraGaojun MaoSameer AwadGaoming MoSameh AttiaGargi JoshiSami Ullah KhanGarikoitz ArtolaSamik DuttaGuptaGasim HayderSamineni PeddakrishnaGatheeshgar PerampalamSamir KhatirGaurab DuttaSamir Kumar SarkarGaurav SharmaSamir QourzalGaurav VarshneySamira NaghdiGaurav YogeshSampath GunukulaGautam SinghSamson AisidaGbadebo OwolabiSamuel D. ClarkeGbenga AsalaSamuel F. RodriguesGebremariam BirhanuSamuel LimGeetha BollaSamuel TulashieGelmires De Araujo NevesSanaz AlamdariGelu Ovidiu TirianSanchita KunduGencay SariisikSanda Liana BaltesGenevieve PalardySandeep AryaGenki YoshikawaSandeep KaushalGennady KolesnikovSandeep S. KoreGennaro Salvatore PonticelliSandeep SonyGeoffrey MitchellSandip SarkarGeoffrey WillSándor ValkaiGeon Joon LeeSandra CruzGeorge Alexandru MafteiSandra I. VieiraGeorge AmuleleSandra JuradinGeorge Chr. PsarrasSandra Kalil BussadoriGeorge D. VerrosSandra KlingeGeorge E. MustoeSandra MaginaGeorge EliadesSandra PereiraGeorge J. BesserisSandra PinaGeorge KalogeropoulosSandrine Bittencourt BergerGeorge KenanakisSandro BitencourtGeorge KyzasSandro TurchettaGeorge Octavian BuicaSang Don MunGeorge PapazafeiropoulosSang Hoon KimGeorge S. KlirosSang Kyoo LimGeorge StavropoulosSang Mo KimGeorge StiubianuSangaraju SambasivamGeorge SyrrokostasSangeeta SantraGeorge TrakakisSangeeta SinghGeorge VekinisSang-Eun BaeGeorge VerrosSanghee KimGeorge XiroudakisSangin ParkGeorgios ArnaoutakisSangmo KimGeorgios BartzasSangmo KooGeorgios BokiasSangmoon ParkGeorgios DimitrakisSang-Soo KimGeorgios E. ArnaoutakisSangwoo RyuGeorgios GiannopoulosSang-Youn KimGeorgios KatsikisSanichiro YoshidaGeorgios MartinopoulosSanja Ercegović RažićGeorgios PampalakisSanja J. ArmakovicGeorgios PipintakosSanja Mahovic PoljačekGeorgios VekinisSanjay A. BhakharGeorgiy KagramanovSanjay Kumar (India)Georgiy ShakhgildyanSanjay Kumar (Singapore)Georgy GrancharovSanjay SinghGerald DrägerSanjeev KumarGerald FranzSanjeev Shashi KumarGerald L. KnappSanjib KarGerardo ArayaSanjoy GhoraiGerardo RosasSanjoy PaulGergely GrétaSankar SekarGerhard MullerSanosh Kunjalukkal PadmanabhanGerhard SwiegersSansit PatnaikGermain BoyerSantanu Kumar DashGermain VallverduSantanu MukherjeeGermán EbenspergerSanthana Krishna Kumar AlagarsamyGermán Sanz LobónSantiago Ferrandiz BouGero BurghardtSantiago Garcia-GrandaGerrit Ralf SurupSantidan BiswasGesmi MilcovichSanto CatapanoGetu HailuSantosh KumarGéza István MárkSantosh SampathGhada Hussein NaguibSaqib AnwarGhader HosseinzadehSaqib HassanGhais KharmandaSara BernardiGhasan Fahim HuseienSara BocchiGhasem SargaziSara ContiGhasemi Seyed HoomanSara García BallesterosGheorge IliaSara KhamsehGheorghe BorodiSara LiparotiGheorghe GurauSara Ojeda-BenitezGheorghe IliaSara PescetelliGheorghe MatacheSara TargońskaGheorghe NagitSarabjeet Singh SidhuGheorghe NechiforSarah A. BentilGheorghe OanceaSarah GassmanGheorghe PaltaneaSarah PixleyGheorghe PascutSaravanakumar RamasamyGhivela Girish ChandraSaravanan Ramiah ShanmugamGholam Hossein RoshaniSarbjeet KaushalGholam Reza KhayatiSarfarazi VahabGholamali ShafabakhshSarhang HamaGholamhosein MoloudianSarila VenukumarGholamhossein SodeifianSarinova SimandjuntakGholamreza KhalajSarkis GavrasGhulam DastgeerSarmiza Elena StancaGiacomo DerchiSartaj Ahmad BhatGiacomo MagnaniSaša ĐurovićGiacomo OteriSascha HeitkamGiada GaspariniSatadru Das AdhikaryGian Andrea PelliccioniSathish Kumar PalaniappanGian Andrea RizziSathish SundarGian Domenico SorarùSathish Sundar Dhilip KumarGian Piero LignolaSathyashankara SharmaGian Pietro De GaudenziSatish B. AlapatiGianfranco CarotenutoSatish VishwanathaiahGianfranco Dell’AgliSatoru KobayashiGiangiacomo MinakSatoru NakashimaGianluca GambariniSatoshi KomasaGianluca GhigoSatoshi YamaguchiGianluca MalavasiSatoshi YokoseGianluca MazzuccoSaturnino Marco LupiGianluca TondiSatyadev RosuneeGianluca ViscusiSatyapaul A. SinghGianluigi AlbanoSaulius DrukteinisGianmaria D’AddazioSaullo G. P. CastroGianpaolo SerinoSaumik Saumik PanjaGianrico SpagnuoloSaurabh ChaturvediGiansergio MenduniSaurabh PathakGianvito ScaringiSaurabh ShuklaGiasemi AngeliSaurav DixitGideon LyngdohSaverio MaiettaGideon RotichSavvas VassiliadisGiedrius JanusasSawanta S. MaliGigliola LusvardiSawsan AlmohammedGilbert BellangerSawyer D. CampbellGilbert De MeySayak DasguptaGilbert HingeSayan GangulyGilberto Gonzalez-AvalosSayan SarkarGilberto J. ArrudaSayandeep GhoshGilda FerrottiSayantan MukherjeeGildardo Solorio-DíazSayed Nasimul AlamGildas DiguetSayed SalehGinka K. ExnerSayyad Zahid QamarGintautas BagdziunasSazmal ArshadGiorgio LucianoScott LeavengoodGiorgio MargaritondoScott MonaghanGiovanna ConcuScott R. RajskiGiovanna GautierSebastian A. KubeGiovanna MurmuraSebastian BalickiGiovanna SotgiuSebastian BalosGiovanna XottaSebastian BonarddGiovanni AngiulliSebastian BürkleinGiovanni BorsoiSebastian DahleGiovanni CagnettaSebastian George MaxineasaGiovanni CapursoSebastian HenkelGiovanni CeccioSebastian KruppertGiovanni E. GiganteSebastian LechGiovanni FalsoneSebastian Marian ZahariaGiovanni FerrarisSebastian MrózGiovanni GiacomelloSebastian PawlusGiovanni MaizzaSebastian SchwamingerGiovanni MatareseSebastian SławskiGiovanni MuciacciaSebastian ZlotnikGiovanni NastasiSebastiano CampisiGiovanni SignoreSebastiano CandamanoGiovanni SpinelliSebastiano GarroniGiribaskar SivaswamySebastiano MariazziGirish KumarSebastiano TomassettiGirolamo CostanzaSebastiano VasiĢirts BūmanisSébastien AlixGirum UrgessaSébastien GrondelGiulia FolpiniSébastien LeveneurGiulia OrilisiSebastien RondotGiulia SerranoSeda ArazGiuliana BancheSedky HassanGiulio MarcheseSee Jo KimGiuseppe Alessandro ScardinaSee Mun LeeGiuseppe ArrabitoSeelam Rajasekhar ReddyGiuseppe BarbieriSegun OgundareGiuseppe CastaldiSeifollah GholampourGiuseppe Domenico ArrabitoSeigo OhbaGiuseppe Emanuele LioSeiji KurodaGiuseppe GoriniSelahattin KocamanGiuseppe LazzaraSelim GurgenGiuseppe Lo GiudiceSelvaraj BarathiGiuseppe MinerviniSelvaraj Rajesh KumarGiuseppe NastiSelvaraj Senthil KumaranGiuseppe OlivetoSelvin P. ThomasGiuseppe PaladiniSemra KuramaGiuseppe PandareseSenay UstunelGiuseppe RicciardiSeng Hua LeeGiuseppe SancataldoSeniz UcarGiuseppe SantarsieroSenka GudićGiuseppe ScarpelliSenka MeštrovićGiuseppe SchiavoneŞenol BayraktarGiuseppe TronciSenthil Kumaran SelvarajGiuseppe Trusso SfrazzettoSeog-Young YoonGiuseppina BalassoneSeok Jae ChuGiuseppina IervolinoSeokbin LimGiuseppina NoccaSeok-Ho RhiGlaucia BressanSeokhoon AhnGlaydson Simões Dos ReisSeokHwan AhnGleb KozlovSeok-Hwan JeongGleb TselikovSeong HuhGlen KelpSeong Min KangGlenn DaehnSeong-Cheol LeeGloria Belén Ramírez RodríguezSeongin MoonGloria I. Dávila-PulidoSeong-Min KimGloria PérezSepanta HosseinpourGnanasambandam AnbuchezhiyanSepehr AlizadehsalehiGo IgarashiSepehr TalebianGo OkadaSepideh AliasghariGobi Saravanan KaliarajSerafino CarusoGobinath RavindranSerena De SantisGohar KhachatryanSerge E. SavotchenkoGokcekaya OzkanSerge Nyallang NyamsiGokhan AydinSergei KomogortsevGokhan GeceSergei KulinichGökhan KaplanSergei NevskiiGökhan KucukturkSergei Pavlovich OsipovGolden KumarSergei PopovGolub VladimirSergei RyzhkovGomaa Abdelgawad Mohammed AliSergei T. MileikoGonçalo TiagoSergeiy KusmanovGong PanSergej GookGong YongzhiSergej HlochGongcheng YaoSergejs GaidukovsGonzalo Garcia FuentesSergey A. KarpushenkovGonzalo M. Dominguez AlmarazSergey A. Stel’makhGonzalo Martínez BarreraSergey A. ZelepuginGonzalo Sanz Diez De Ulzurrun CasalsSergey Alexandrovich UporovGoopyo HongSergey AnanyevGopala Krishna PodagatlapalliSergey Anatolievich GhyngazovGopalakrishnan GopuSergey BelimGopinathan JanarthananSergey BelyakovGoran GazićSergey DubinskiyGoran M. MladenovićSergey Fedorov (Moscow StateGoran VukelicSergey Fedotov (Mendeleev Gordana Topličić ĆurčićSergey FortunaGordana Topličić-ĆurčićSergey G. AnikeevGordon James ThorogoodSergey GorelickGordon NameniSergey GudkovGordon T. YeeSergey I. MoseenkovGoreti PereiraSergey I. NikitenkoGorica StanojevicSergey IlyinGorke Gurel PekozerSergey KidalovGorkem MemisogluSergey KobtsevGoshtasp CheraghianSergey KomarovGour Mohan DasSergey KonovalovGouri S. JasSergey KudryashovGoutam Singh NingombamSergey MoseenkoGovhindhan GnanamoorthySergey PaninGovind ChilkoorSergey PasechnikGovindan Suresh KumarSergey SarkisovGowrishankar M. C.Sergey ShevtsovGowthaman SivakumarSergey TimofeevGraham DavisSergey UspenskiiGratiela PircalabioruSergey UvarovGrazia Giuseppina PolitanoSergey V. DmitrievGrazia Maria Lucia MessinaSergey V. KolotilovGraziano UbertalliSergey V. PrikhodkoGrazyna AdamusSergey VereshchaginGrazyna Simha MartynkovaSergey VinokurovGrega KlančnikSergey Yu. SavinGregorio F. OrtizSergey Zakharovich SmirnovGregorio GarciaSergey ZharkovGregory ChassSergey А. KukushkinGregory P. CarmanSergio Alberto DassieGregory TricotSergio Alfonso Pérez-GarcíaGreice K. B. Da CostaSergio Antonio Marques LimaGrigory V. ZyryanovSergio CalixtoGrigory YakovlevSergio CiceroGrzegorz AdamekSergio D’AddatoGrzegorz BudzikSérgio Frascino Muller De AlmeidaGrzegorz ChladekSergio LorenziGrzegorz DombekSergio M.O. TavaresGrzegorz GumiennySergio Orazio BattiatoGrzegorz JanowskiSergio Rius-PérezGrzegorz KiesiewiczSérgio RodriguesGrzegorz KomarzyniecSergio Rojas-BuzoGrzegorz Ludwik GolewskiSergio SpinatoGrzegorz MakomaskiSergio ZamoraGrzegorz MatulaSergiy YepifanovGrzegorz MazurekSergo RekhviashviliGrzegorz MieczkowskiSerguei Alejandro-MartínGrzegorz MusielakSerhat ÇeliktenGrzegorz SamołykSerhat ŞapGrzegorz SkrabalakŜerife Pınar YalçınGrzegorz SochaSerkan DündarGrzegorz StradomskiSertac KarakasGrzegorz StruzikiewiczSeshadev SahooGrzegorz TęczaSethupathi VelmuruganGrzegorz TrybekSetyo Budi KurniawanGrzegorz TrzcinskiSeung Gu ShinGrzegorz WalowskiSeung Hwa YooGrzegorz ZielińskiSeung Koo ParkGuadalupe Alan Castillo RodríguezSeung Yun NamGuanghua CaiSeung-Bok ChoiGuangji XuSeunghan OhGuangjiang LiSeungil KimGuanglei WuSeungjun LeeGuangping HuangSeungyun LeeGuangping SunSeval GunduzGuangtao ZanSever MicanGuangwu FangSevgi PolatGuangyi LiSevki CesmeciGuangyi MaSeweryn LipińskiGuangyu LiuSeyed Abdollah Mansouri MehryanGuangyu ZhuSeyed Ali GhahariGuangze YangSeyed Ali MosayebiGuanqiu QiSeyed Mo MirvakiliGuanying ChenSeyed Mohammad Mirsoleimani AziziGuanzhou ZhuSeyed Reza OmranianGubbala V. RameshSeyed Sina MousaviGue Hyun KimSeyede Raheleh YousefiGuenter LorenzSeyedeh Mehri HamidiGueorgui GueorguievSeyedfakhreddin NabaviGuglielmo CampusSeymur HasanovGui Lin SheSeyyed Alireza HashemiGuicai LiuSeyyed Mehdi KhoshfetratGuido BelforteSeyyed Mojtaba MousaviGuido GrilliSezgin ErsoyGuido PanzarasaSha LiangGuido Van OostShaban Ali ZangenaGuijian XiaoShabbir MemonGuilherme AscensãoShabi Thankaraj SalammalGuilherme José Cunha GomesShabir Hussain KhahroGuilhermina Ferreira TeixeiraShabnam Sadeghi EsfahlaniGuilin WuShadi HoushyarGuillaume NatafShafaqat SiddiqueGuillaume PolidoriShafreeza SobriGuillermina BurilloShaghayegh KarimzadehGuillermo A. RiverosShah RukhGuillermo AhumadaShahed RasekhGuillermo C. Mondragón RodríguezShahid AdeelGuillermo Garcia ToralesShahid AliGuillermo GrazioliShahid UllahGuillermo Guerrero-VacaShahin KhoddamGuillermo HaukeShahir Mohd YusufGuillermo Riesco MunozShahir YusufGuipeng LiuShahood Uz ZamanGuiseppe PintaudeShahram AjoriGul RahmanShahram HadianjaziGulam Mohammed Sayeed AhmedShahram SolaymaniGulsu SimsekShahzad NaseemGunasekaran MuraliShahzada Qamar HussainGundula Gesine Schulze TanzilShaik Gouse PeeraGundula HelschShaik HussainGunel HuseynovaShailendra RajputGunnar SuchaneckShakeel Ahmad KhandayGuo ChenShakhawat HossainGuo Dong GohShalini MohantyGuo HongweiShamik BasakGuo JiangShams IssaGuo Liang GohShamshuddin M. D.Guodong CuiShan GaoGuodong RaoShangcong ChengGuofang ZhangShangli DongGuofeng WangShangwu DingGuojiang WanShangzhi ChenGuoli TuShanmugam PraveenkumarGuoliang ShangShanmugam SumathiGuoqiang RenShanmugan SengottainGuoqing WangShannon RihaGuosheng HuangShanshan HuGuoxi LuoShaochuan FengGuoyin ZuShao-Hsien ChenGuo-zheng QuanShaohua JiangGurinder Singh BrarShaomian YaoGurjot S. DhaliwalShaoping QianGurminder SinghShaoqing WangGurpreet SinghShaoting LinGururaj BolarShao-Yi HsiaGururaj Kudur JayaprakashSharad UpadhyayGururaj ParandeSharafaldin Al-MusawiGustavo Cabrera BarjasSharanabasava V. GanachariGustavo Filipe PintoSharareh ShirzadGustavo Henrique NalonSharat Chandra BarmanGustavo PintoSharifu UraGustavo Roberto CointryShariq NajeebGustavo SavarisSharnappa JoladarashiGustavo SuárezSharon Kao-WalterGutturu Rajasekhara ReddyShashi PrakashGuy LadamShatrughan SorenGuy PaicShawn David PollardGween Le-SaoutShawn PollardGyorgy DeakShawn SanctisGyorgy SzekelyShayfull Zamree Abd RahimGyörgy ThalmaierShazia HasanGyo-woo LeeShebin WangGyubaek AnSheelan Mahmoud HamaGyula GrófShehata E. Abdel Raheem RaheemGyula VargaShenbaga Velu PitchumaniGyuyong KimShengbo GeH. C. Ananda MurthyShengchuan WuH. M. EnginsoyShengen ZhangH. S. KumarShengfang ShiHa Bich TrinhShengfeng GuoHabibe DurmazShengfu YuHaci BaykaraSheng-Heng ChungHacı SaglamShenghua WuHadi AryanShengjie YaoHadi GholamiyanShengli JinHadi HadshemzadehShengnan LuHadi HasanzadehshooiiliSheng-Po ChangHadi RastinShengwen TangHadi RezazadehShenlong ZhaoHae Hyoung LeeSher Afghan KhanHafeez RahmanSher Singh MeenaHafiezal RadziSherif El BadawyHafiz Muhammad Salman AjmalSherwan NajmHafiz Suliman MunawarShi TangHafize Nagehan KoysurenShiaowei KuoHagay SlutzkyShib Shankar BanerjeeHaggeo DesirenaShibnath SamantaHai T. NguyenShichao LiuHai Yen Thi NguyenShichen SunHaibo GongShifa WangHaibo GuoShifeng DaiHaiBo YangShifeng LiuHaibo YuShifra LevartovskyHaichao AnShigeru YaoHai-Chou ChangShigetaka OkanoHaider AliShih-Hsin HoHaifeng DuShih-Jye SunHaifeng LuShijian WuHaihua XuShikha AwasthiHaile YanShikhgasan RamazanovHailu YangShilpa BhandiHaim AbramovichShimaa El-HadadHaim GrebelShin-Ichi InoueHaining LiuShinichi TashiroHaipeng JiShinichiro AdachiHaiping ZhuShinji TakemotoHairui YangShin-Liang ChenHairul HamzahShinobu FujiharaHaitao MaShintaro SukegawaHaixin GuoShiqiang GongHaixing FangShirin NasresfahaniHaiyan MaoShitanshu Shekhar ChakrabortyHaiyan PeiShiuh-Hwa ShyuHaiyan TangShiv Dutt PurohitHaiyou HuangShiv K. SharmaHajir KarimiShiva GorjianHakan DoganShiyao LinHakan F. ÖztopShiyu YueHakan GürünShiyuan LiuHakeem NiyasShmuel SamuhaHakim AbdelgaderShobhika ParmarHalil I. CiftciShobhit K. PatelHalina KrawiecShohei TashiroHalit S. TurkmenShoichi HirosawaHalldor Gudfinnur SvavarssonShoji TakenakaHaluk KaracaShokouh AttarilarHalyna KlymShotaro NishitsujiHamdani Syed Talha AliShouhu XuanHamdy AbdelkaderShoune XiaoHamdy F. M. MohamedShoyeb ShaikhHamed Aghajani DerazkolaShoyebmohamad F. ShaikhHamed Alipour BanaeiShozo NakamuraHamed DabiriShravan MuthukrishnanHamed KhezrzadehShrawan Kumar MishraHamed MirzadehShreyas KuddannayaHamed NosratiShri Kant TripathiHamed SabetShu WangHamed SafarpourShuai CaoHamed Salimi-KenariShuai LouHamid Bakhsheshi RadShuai QianHamid Farrokh GhatteShuai Wang (Central South University)Hamid KhoshdastShuai Wang (Huazhong UniversityHamid OsmanShuai ZhouHamid Reza AbediShuaihang PanHamid Reza LashgariShuaiming HeHamid Reza Rezaei AshtianiShuang HanHamidreza BagheriShuang ShiHamidreza KoohdarShuanglong FengHamit AdinShuangxi WangHammad KhalidShuangyang ZouHamouda M. MousaShubham SharmaHamza Youssef IsmailShubhangi KhadtareHamzeh HaghshenasShubhangi ShuklaHan FuShu-Fen ChuangHan HanShufeng LiHan XiongShuhua MaHana CharvatovaShuiquan DengHanan ElsisiShuitao GuHanfei MeiShuko SuzukiHang Nga MaiShuli FanHang QiShunbo LiHangfeng ZhangShunpeng ZhuHanieh KhaliliShun-Xing LiangHanif HaidariShunyi JianHanna A. DabkowskaShun-Yi JianHanna PazniakShuo SuiHannes RijckaertShuo YanHannu-Pekka KomsaShuo YangHans A Eyeghe BickongShusen WuHans Eckhardt SchaeferShuxin LiHans HartnagelShuye ZhangHans Martin SauerShuyou WangHans RoggendorfShuyu SunHans Rudolf WenkShweta DabetwarHanuma Reddy TiyyaguraShweta J. MalodeHanuman JatavShyam S. PandeyHany Abd El LateefShyh-Shin HwangHany AbdoSi ChenHany AmmarSi LiHany Ezzat KhalilSiamak EpackachiHao ChenSiamak GhorbaniHao LuoSiamak PedrammehrHao PengSibele B.C. PergherHao Shen (China)Sid Ahmed SlimaneHao Shen (USA)Sidhnath SinghHao Wang (Jinan University)Sidi ZhuHao Wang (University Sidra SaleemiHao Wang (USA)Sigitas KilikevičiusHao Wen ChengSigitas TamuleviciusHao WuSigong ZhangHao YangSih Ying KongHao Yu (Southwest Petroleum University)Sihan ChenHao Yu (University of Science Sihyun LeeHao Yu (USA)Sikandar KhanHaohao DingSilva JoãoHaoran QuSilvana AlfeiHaoran XueSilvana NisgoskiHappy Nkanta MondaySilvana PrekratHaralampos N. MirasSilverio CocoHarald G. DillSilvestre Bongiovanni AbelHaraprasad MondalSilvia Alice LocarnoHarekrushna SutarSilvia BarbiHaridharan KanthamaniSilvia Becerra-BayonaHarifidy RanaivomananaSilvia CarlottoHarikumar RajaguruSilvia CarvalhoHarish Kumar ChopraSilvia MartuHarish RajanSílvia Pérez-RafaelHarishchandra LanjewarSilvia RavelliHarm Van ZalingeSilvia SantiniHaroutioun AskanianSilvino Dias CapitãoHarshit MahandraSilvio SciortinoHartmut GliemannSilviu-Gabriel StroeHartmut HillmerSima Aminorroaya YaminiHartmut KutzkeŠimáček PavelHartmut R. FischerSimge Taşar FarukHaruyuki IshiiSimin SharifiHasan DemirSimon A.M. HespHasan Hamodi JoniSimon MerminodHasan J. KhanSimon MichauxHasan OzturkSimon OmanHasan SajjadiSimon SteinbergHasan Zuhudi AbdullahSimon WangHasanain Radhi RadeefSimona CaprarescuHashem Mousavi AnijdanSimona MiclausHashmi Muhammad AliSimona MunteanHasi Rani BaraiSimona PopescuHassan A. AlshahraniSimona VarvaraHassan AbdoosSimonas RamanavičiusHassan AlgadiSimone BeninatiHassan Amer AlgaifiSimone CiampiHassan GhanemSimone DinarelliHassan HassanabadiSimone DonadelloHassan OuachtakSimone FaillaHassan S. AshourSimone GalloHassan WaqasSimone PiacentiniHassane LgazSimone Quondam AntonioHatem FouadSimonida TomićHawreen Hasan AhmedSin Young KimHayder AbdullahSinan KutluayHaytham El-gazzarSindhu SubramanianHe LiSindhuja ManoharanHe LiangSinead O’HalloranHe Liu (Canada)Singanahalli Thippa Reddy ArunaHe Liu (Chinese Academy of Forestry)Singh KulvinderHe ZhengSipokazi MabuwaHeba Y. ZahranSirajuddin AhmedHéctor GarcíaSirasit SrinuanpanHéctor García-MartínezSirinya ChantarakHéctor Vega CarrilloSiro CasoloHee Chul LeeSitakanta SatapathyHee-Joon KimSiti BaidurahHeeyoung LeeSiti Hasnah KamarudinHeiddy P. QuirozSiti Maznah KabebHeinz Werner HöppelSiti Nooraya Mohd TawilHejie YangSiti Noorbaini SarminHélder David CraveiroSiti Zuliana SallehHélder PugaSiu Hong WongHélder S. SousaSiu Wun CheungHelena BragaSiva MuruganHelena CastánSiva Surya MulugundamHelena CorvachoSivakumar RamanathanHelena Cristina VasconcelosSivanandham AravindanHelena FelgueirasSivaramalingam AnandhiHelena Otmačić ĆurkovićSivasankar KoppalaHelena TeteryczSivasankaran SivanandamHélène DebédaSixun ZhengHélène GaraySk Faisal KabirHelinando Pequeno De OliveiraSkorjanc TinaHellen JinSkotnikova Margarita AlexandrovnaHelmut KlöckerSlavko RakicHema AchyuthanSlavko RupcicHemali RathnayakeSlavomir HrcekHemant JainSławomir AlabrudzińskiHena DasSławomir CięszczykHend Ali OmarSławomir CzarneckiHeng Li HuangSławomir Kula‪Hengxu SongSławomir KuleszaHenrich FrielinghausSławomir PoskrobkoHenrik SaxenSlawomir SujeckiHenrique Almeida SantanaSlawomir SwiradHenrique Duarte Da Fonseca FilhoSławomir ZimowskiHenrique Leonel GomesSlim NaifarHenryk BalaSlobodan MitrovicHenryk BednarskiSneha SamalHenryk KaniaSnehashis PalHerda Yati Binti KatmanSnehsheel SharmaHeri KusumaSnezana Ilic-StojanovicHeri SutantoSnezana NenadovicHeriberto Pérez AceboSnežana VučetićHerman PotgieterSnježana Firšt RogaleHermínio P. DiogoSnjezana Tomljenovic-HanicHernando S. Salapare IIISobhan RoshaniHerve DegeeSobhi DanielHervé ReychlerSobia NoreenHervé VezinSofia GambaroHerwig MayerSofia JavedHesam KamyabSofia MasiHesam PouraliakbarSofia MorozovaHesham M. H. ZakalySofia SoaresHesham TawfeekSofya YarusovaHessamoddin MoshayediSofyan A. TayaHetuo ChenSohail M. A. Khan MohammedHeung-Kyu KimSohail NadeemHicham AbououalidSohail R. ReddyHicham AguenySoham GhoshHicks PeterSohan SinghHideaki KatogiSoheil GhadamiHideaki SawadaSoheil GohariHidekazu TakahashiSoheil MohtaramHideki KitaSoheil SarabandiHidetomo UsuiSohini ChowdhuryHigashisaka KazumaSohrab RahimiHikaru KoboriSojobi Adebayo OlatunbosunHikmet KoyunbakanSolaiappan AnanthakumarHilal El-HassanSolehuddin ShuibHilal RedaSoleiman BourrousHilario VidalSolomn AdedokunHilke BahmannSomak BhattacharyyaHilmar DanielsenSomasundaram KarthiyainiHimani PuniaSomasundaram PrasadhHipólito SousaSomasundaram SaravananHiran LalSomnath ChattopadhyayaHiroaki HanafusaSompit WanwongHiroaki KikuchiSompol SkullongHiroaki MaedaSompote YouwaiHiroaki SatohSon Thanh NguyenHirokazu MasaiSoner TopHiroki IshibashiSong HuHiroki MatsuoSong-Jeng HuangHiromu SaitoSonglin DingHiroshi IshihataSonglin ZhangHiroshi MaiwaSongqi MaHiroshi YasudaSonia García RodríguezHiroshi YokotaSonia MarfiaHiroshi YoshiharaSónia SimõesHiroyoshi TanakaSonu GandhiHiroyuki ArafuneSoohwan JangHiroyuki MiyamotoSoo-Hyun JooHiroyuki MuramatsuSoorathep KheawhomHiroyuki SaitohSophia TsipasHiroyuki UsuiSophie CoxHiroyuki YamadaSoram OhHisaki WatariSoran BiroscaHisashi HayashiSorb YesudhasHitesh BorkarSören ÖstlundHitesh VasudevSorin Vasile SavuHizb SajidSorin VlaseHo Pham Huy AnhSoslan A. KhubezhovHoa Le QuynhSossio Fabio GrazianoHoang Anh NguyenSotiris DemisHocheon YooSotomi IshiharaHong Duc PhamSoubantika PalchoudhuryHong LeiSougata GhoshHong LiSougata RoyHong Seok KangSouhir ZghalHong Wu SongSoumen DasHong Yu YangSoumitra SulekarHong ZhaoSoumya J. RayHongbo MuSoumya SridarHongbo XuSourangsu BanerjiHongchang LiuSourav DeHongcun BaiSourav Kr. SahaHongfang ChenSouraya Goumri SaidHongfu ZhangSouvik DasHonghao HouSouvik PalHonghong LinSovann KhanHongliang HeŠpiro IvoševićHongliang ZhangSpyridon GrammatikopoulosHongliang ZhaoSrboljub SimićHongling SunSrđan D. KostićHongping HuSrećko ManasijevićHongpo WangSrecko StopicHongti ZhangSreco D. SkapinHongwang ZhangSree Rama Chandra TammanaHongwei BaiSreed Sharma KanakkillamHongwei YangSreedevi UpadhyayulaHongxiang HuSreejith NanukuttanHongyu LiSreejith SivaramapanickerHongyuan ZhouSreekanth GinnaramHongzhang DengSreekanth J. VarmaHongzhang SongSreeprasad T. SreenivasanHoracio Andrés PetitSreeraj PuravankaraHoraţiu VermeşanSreevatsan RaghavanHorea Florin ChicinasSri AtmajaHoria ConstantinescuSri Atmaja P. RosyidiHoria Eugen PorteanuSridhar ChandrasekaranHoria HaragusSriharsha HegdeHosam M. SalehSrikanth ItapuHosein EshghiSrikumar GhoruiHoseok NamSrinivas SistlaHossam El Din M. SallamSrinivasan Vinju VasudevanHossam M. YehiaSriram GanesanHossein BaniasadiSriram GopuHossein BaraniSriram VaidyanathanHossein Ghasemi TabasiSriwidodo SriwidodoHossein Maleki-GhalehStamatis BoyatzisHossein MinoueiStanislav A. ChupinHossein MokhtarianStanislav A. EvlashinHossein MolaviStanislav KurajicaHossein NassiraeiStanislav MrazHossein TaghinejadStanislav RuszHossein TaghipoorStanislav ZabotnovHossein Talebi-GhadikolaeeStanisław Andrzej RóżańskiHossein VafaeenezhadStanisław CudziłoHossein VatandoostStanisław KowalakHou Guang ChenStanisław KrompiecHou YanbeiStanisław LipińskiHouk JangStanisław PietrzykHoussem BoulebdStavros KoulouridisHow Wei Benjamin TeoStavros PissadakisHrčka RichardŞtefan AntoheHrishikesh DasStefan CsakiHristo P. PenchevStefan GhimisiHrudananda JenaStefan HeunHrvoje GebaviStefan Ioan VoicuHrvoje SmoljanovićŠtefan KohekHryhoriy NykyforchynStefan KrakowiakHsien-Kuang LiuStefan KranzHsiharng YangStefan MocanuHsing I HsiangStefan PalisHsiu Wei ChengȘtefan ȚăluHsusheng TsaiStefan ValkovHu LiStefan WagnerHua DingStefania BenedettiHua KeStefania ImperatoreHua ZhuStefania PragliolaHuacheng ZhangStefania Roberta CiccoHuagang ZhangStefania VitaleHuajie YangStefan-Lucian TomaHuan LiuStefano AneliHuan WangStefano BellucciHuan XiStefano CarliHuanda ZhengStefano CicchiHuanhuan FengStefano ColumbuHuaping WangStefano EramoHubert AnyszStefano GariglioHubert KrennerStefano MarchesiHubert RahierStefano PaganoHuei-Sen WangStefano SalgarelloHueliton Wilian KidoStefano VeronesiHugo A. L. FilipeStefanos ChaitoglouHugo Alexander Rondón QuintanaStefanos KikionisHugo FernandesStefanos MourdikoudisHugo José Nogueira Pedroza Dias MelloStefanos PapanikolaouHugo M. OliveiraSteffen BergHugo Rojas-ChávezSteffen WitzlebenHugo SobralStela GeorgievaHui Li (Northeastern University)Stelian Sergiu MaierHui Li (Shenzhen Technology University)Stella RaynovaHui Li (Tongji University)Stepan A. ShcherbininHui Li (Wuhan University)Stephan BodenHui LuoStephan KubowiczHui SunStéphane DanieleHui WangStephane DiringHui YaoStéphane DurualHui ZhangStephane HocquetHui-Bin WuStephane PanierHuihui LiStephanus P. Du PreezHuijun XieStephanus Petrus Du PreezHuiliang CaoStephen Siu-Yu LauHuilong WangStephen WalleyHuimin LiStephen WorrallHuimin XieStephen YoungHuiqian LuoSteve GreenbaumHuiqun WangSteven A. PolicastroHuixiang ChenSteven G. JanstoHulusi ÖzkulSteven Solethu NkosiHumaira SeemaSteven W. BucknerHumberto YoshimuraStjepan SpaljHumphrey DansoStoja ReškovićHung Lung ChouStrzemski MaciejHung Tran NguyenStyliani PapatzaniHusam HusseinŞuayip Burak DumanHussam AlghamdiSubaer JunaediHussein H. ZghairSubba Rao CheekatlaHussein Mahmood HamadaSubbarayan SivasankaranHussein Mohamed ElhusseinySubhadip MondalHussien HegabSubhajit RoychowdhuryHussin AhmedSubhan SalaehHwajoo JooSubhasish DasHye Jin KimSubhra MajhiHyeong Ki KimSubir Kumar JuinHyosim KimSubrahmanyam ChallapalliHyoung Ho KimSubramaniam ArulkumaranHyun Do YunSubramaniam ShankarHyun Ho LeeSubramanian MuthaiahHyun Hwan KimSubramanyan VasudevanHyun Woo KimSubrata Kumar PandaHyun Wook JungSuchada KongkiatkamonHyunbok LeeSucharat LimsitthichaikoonHyungdae BaeSuchart LimkatanyuHyungjun SongSuchart SiengchinHyungSeok SeoSuchinder K. SharmaHyunik YangSuchithra Ashoka SahadevanHyunseok KoSuchithra Poilil SurendranHyun-Sik KimSudha JosephI. Chung ChengSudhakar NarraI. Jénnifer GómezSudhansu Ranjan DasI. Ta LeeSudheendran MavilaIakov LyashenkoSudhir KumarIan HartleySudhir Mohan KumarIan IngramSudhir Singh BhadauriaIan LianSudhy S. PanickerIan SmithSudip BanerjeeIan TaylorSudip MaityIan TeasdaleSufyan GaroushIanasi CatalinSugato HajraIaroslav GnilitskyiSugrib ShahaIbham VezaSuhail KhanIbrahim AbdulhalimSuhana Binti KotingIbrahim AbotalebSuhas SuhasIbrahim AgwaSui MaiIbrahim AlpSujatha RamaniIbrahim AlshaikhSujay NagannaIbrahim ÇobanoğluSujeet KumarIbrahim DemirciSujit Kumar PradhanIbrahim M. Abdel-MotalebSujit PardeshiIbrahim M. El-KattanSuk Hoon KangIbrahim M. SayedSukanta BhowmickIbrahim M.A. MohamedSukanta Kumer ShillIbrahim MahariqSukeharu NomotoIbrahim Mohamed (Benha University)Sukhoon PyoIbrahim Mohamed (Sohag University)Sukhpal SinghIbrahim ShaabanSuleyman AkpinarIbrahim Y. HakeemSüleyman GökçeIdalina J. DomingosSuman ChatterjeeIeva MisiunaiteSumant DwivediIfeyinwa I. ObianyoSumanta Kumar SahooIftikhar Ahmed ChannaSumanta SahooIgnasi CasanovaSumedha LiyanageIgnazio BlancoSumera NazIgnazio RoppoloSumesh ArrangotIgor AvetissovSumi JoIgor BuravlevSumit GhoshIgor E. VyaliySumita GoswamiIgor ElmanovichSumon RezaIgor GolubSumra Wajid AbbasiIgor I. PonomarevSun MengtaoIgor IatsunskyiSundar KunwarIgor KarlovitsSundararajan NatarajanIgor LitvinchevSuneetha VuppuIgor PaštiSung Bo LeeIgor S. YasnikovSung Gu KangIgor SchreiberSung Hwan HongIgor ShchetininSung Jae JeonIgor SmolyaninovSung Oh ChoIgor SolodovSung Woon ChoIgor V KhudyakovSungchan NamIgor V. RozhanskySungchul YangIgor V. ShevchukSunghan KimIhor KindratSungho SongIhor S. VirtSungjun KimIkki TateishiSung-Kyo KimIkmal Hakem A. AzizSungmook JungIl KimSung-Nam KwonIlaksh AdlakhaSungwook KangIlare BordeaşuSunil K. SharmaIlaria CacciariSunil Kumar RamamoorthyIlaria PapaSunil Kumar SharmaIlchat SabirovSunil Kumar SinghIldikó HarsányiSunil RaykarIldiko PeterSuniti SuparpIleana IeloSun-Jin HanIleana LickSun-jung YoonIleana Nicoleta PopescuSun-Kyu LeeIleana RãdulescuSuode ZhangIleana RauSupandeep Singh HallanIlenia FarinaSupandi Supandiİlhami DemirSupansa YodmuangIl-Hwan ChoSupratim DasIliana Medina-RamírezSupriya GhoshIliya PetrievSupriyo BandyopadhyayIlker Bekir TopcuSurachai KarnjanakomIlker TorunSuraj JoshiIlkeun LeeSuraj ThiagarajanIlknur AltinSurajit BhattacharjyaIllariia RazumkovaSurekha YadavIllyas Bin Md IsaSurender SinghIlman Nuran ZainiSurendra Krushna ShindeIlona JastrzębskaSuresh K. VermaIlsiya M. DavletbaevaSuresh KumarIlsun YoonSuresh Kumar JakkaIlya G. ShenderovichSuresh Kumar KailasaIlya I. TumkinSuresh SagadevanIlya KorolkovSuresh ThanguduIlya MishakovSuresha BheemappaIlya V. KorolkovSuriati GhazaliIlya V. OkulovSurya Veerendra Prabhakar VattikutiIlya V. RoslyakovSuryadi IsmadjiIlya ZhukovSusai RajendranImad HussainSusana FernándezImad Shakir AbboodSusana RibeiroIman El-MahallawiSusana SérioIman GhamarianSusanne HemesIman ParisaySusanne K. WocheIman RoqanSusham BiswasIman SantosoSushobhan PradhanImants KaldreSutasn ThipprakmasImelda Olivas ArmendárizSuvash Chandra PaulImhade Princess OkokpujieSuwat NananImran AliSuzan A.A. MustafaImran QureshiSuzan S. IbrahimImran ShahSuzana FilipovicImre KissSven VogelImre Norbert OrbulovSven WinterImtiaz Ahmad (Pakistan)Svetislav SavovicImtiaz Ahmed (UK)Svetlana BatashevaIn Seok YoonSvetlana NalimovaIñaki GarmendiaSvetlana Petroetrovna BuyakovaInayatur RehmanSvetlana PolivtsevaInderbir SinghSvetlana PushkarÍndia Olinta De Azevedo QueirozSvetlana SaikovaIndrani ChoudhuriSvetlana SushkovaIndrek MustSvetlana UshakIndu Siva Ranjani GandhiSvetlana V. SamchenkoInes DespotovicSvetlana Von GratowskiInes FasolinoSvetoslav KolevInés IvañezSvitlana AlyokhinaInes KovačićSvitlana KopylInês Morais CaldasSwadipta RoyInes PrimožičSwagato DasInes RiechSwaleha NaseemInese FilipovaSwarniv ChandraInesh KenzhinaSwarom KanitkarIngars ReinholdsSwarup RoyIngeborga AndersoneSwati ParmarIngrid DelyováSwee Leong SingIngrid TonniSyarida Hasnur SafiiIngrid ZnamenáčkováSyazwani IdrusInna LisnevskayaSydney Ferreira SantosIoan ArdeleanSyed Farid Uddin FarhadIoan AschileanSyed Fida HassanIoan PeteanSyed Minhaj Saleem KazmiIoana Cătălina GîfuSyed Modassir HussainIoana CsakiSyed NabilIoana DemetrescuSyed Rameez NaqviIoana Dorina VlaicuSyed Sajid Ali GillaniIoana GhiutaSyed Salman ShafqatIoana Maria CorteaSy-Hann ChenIoana StanculescuSylvain DantoIoannis A. BarboutisSylvain GirardIoannis DassiosSylvain QueyreauIoannis FourmousisSylwester KłyszIoannis KaratasiosSylwia GolbaIoannis N. TsimpanogiannisSylwia RóżańskaIoannis TheologidisSzabó Ferenc JánosIoannis TsivintzelisSzabolcs FischerIolanda De MarcoSzabolcs FogarasiIon CalinaSzabolcs MuráthIon Dragos UţuSzilvia KlebertIon PenceaSzymon DawczynskiIon SavaSzymon ŁośIon Vasile VanceaSzymon MalinowskiIonel ChicinasSzymon SkibickiIonela Andreea NeacsuT.P. MeikandaanIonela Ruxandra SfeatcuTachita Vlad-BubulacIonut Cristian RaduTadahiko IshikawaIonut LuchianTadas DambrauskasIonut Ovidiu TomaTadashi KawaiIonuţ Ovidiu TomaTadesse Gemeda WakjiraIosif Vasile NemoianuTadeusz HryniewiczIra ValenzuelaTadeusz ŁagodaIraklii I. EbralidzeTadeusz Mikolaj MuziolIran Rocha SegundoTadeusz MikolajczykIrena KratochvilovaTadeusz SalacinskiIrena M. HlaváčováTadeusz SiwiecIrene Buj-CorralTadeusz SzumiataIrene FassiTadeusz SzymczakIrene LingTae Whan HongIrene Pina-VazTae-Hoon KimIrene Samy FahimTaek Yong HwangIrene VillaTaekeun OhIrene VoukkaliTaeseon LeeIreneusz MarzecTae-Sik OhIreneusz SzachogluchowiczTaewan KimIrfan BahiuddinTaffese WoubishetIrfanul Haque SiddiquiTaghreed Khaleefa Mohammed AliIrina CherunovaTaha A. El-SayedIrina I. BuchinskayaTaha BakhshpooriIrina IelciuTaha Koray ŞahınIrina MakarovaTaha RashidIrina NegutTahar TayebiIrina PushkarevaTaher TawfikIrina SavinaTahiana RamananantoandroIrina SmolinaTahir FarooqIrina V. KasyanovaTahir IqbalIrina V. KozerozhetsTahir MuhmoodIrina V. SemenovaTahir NooraniIrina Ya MittovaTahmid RupamIrina YushinaTaia Abd El-MageedIrina ZgurăTai-Cheng ChenIris WeberTai-Feng HungIrmgard FrankTaishi YokoiIryna GolovinaTaisiya KozlovaIryna YaremchukTaj Muhammad KhanIs FatimahTakahiko BanIsa AnshoriTakahiko Morotomiİsa SıdırTakahiro KannoIsaac B. BersukerTakahisa ArimaIsaac Montoya De Los SantosTakanori KuboIsaac RampediTakao YamamotoIsaac Torres DiazTakashi IkunoIsabel Lado-TouriñoTakashi OkadaIsabel Pestana Paixão CansadoTakashi OkijiIsabella BiancoTakashi SagawaIsabella Natali SoraTakashi YanaseIsabelle FavierTakayuki IkedaIsam AliTakayuki TsukegiIshak ErtugrulTakayuki YanagidaIshikawa NoritoTakehiko YoshimitsuIshwor KhatriTakeshi IwamotoIskra Z. KolevaTakeshi MitaniIslam A. AbdelhafeezTakeshi ShimomuraIslam IbrahimTakuichi SatoIslam MinisyTakuo SakonIsmael AcostaTakuro MasumuraIsmael Flores VivianTakuya HayashiIsmail AbdulazeezTakuya IsonoIsmail ElkhrachyTakuya MiuraIsmail FidanTalaat El-Desoukeyİsmail İnceTalal S. AmhadiIsmail IsmailTalat BaranIsmail LuharTales De Vargas Lisbôaİsmail ÖçsoyTalha YaqubIsmat H. AliTalib M. AlbyatiIsmeli Alfonso LópezTam Ka MingIsrael AlshanskiTamara TomašegovićIsrael FelnerTamás KolonitsIsrar UddinTamás SoványIsrat AliTamás TörökIstván BudaiTamer El-MaaddawyIstván FábiánTamerlan T. MagkoevIstvan JanossyTan Jin SooIstvan RajtaTan N. NguyenItthipon JeerapanTanay KunduItzhak OrionTancu Ana Maria CristinaItziar Serrano MunozTandra GhoshalIuga MadalinaTania FrancisIulian AntoniacTanja PušićIulian PanaTanja ZidaričIuliana MotrescuTanmay KulkarniIuliana SpiridonTanmoy MukhopadhyayIuliia NovoselovaTanmoy PaulIurii KogutTanveer Ahmad TabishIva ViehmannováTanvi GovilIvaldo ItabaianaTanvir QureshiIvan A. BoldyrevTanya BuddiIvan A. ParinovTanya CamachoIvan A. ShishovTao FuIvan D. GrishinTao HuaIvan FortelnýTao HuangIvan GabrijelTao YangIvan GajdošTao ZhangIvan GitsovTao-Hsing ChenIván Herrero-DuráTapabrata MaityIvan IovkovTapan Kumar DasIvan JandrlićTapan Kumar PalIvan JirkaTapan MukerjiIvan KhmelnitskiyTapas GhatakIvan KrausTapas K. MandalIvan LomakinTar IldikóIvan LukačevićTaras DemkivIvan MarićTarek AllamIvan MikheevTarek BachaghaIvan NemecTarek HidouriIvan Nikolaevich ErdakovTarek MohamedIvan ParinovTarek Saleh AmerIvan PelevinTareq ManzoorIvan PetryshynetsTareq SaeedIvan ProchazkaTariq Al-MansooriIvan RužiakTariq AzizIvan S. StefanovićTarun AgarwalIván SciscenkoTatacipta DirgantaraIvan ShatskyiTatheer ZahraIvan SkvortsovTatiana A. KalashnikovaIvan V. SmirnovTatiana AkopovaIván Valdivia-GandurTatiana KalashnikovaIvana BarisicTatiana N. MajsoedovaIvana CarevicTatiana N. MyasoedovaIvana GobinTatiana S. PerovaIvana IvanićTatiana SafronovaIvana L. LehrTatjana GricIvana LukićTatjana JantzenIvana PanžićTatjana ŠoštarićIvana PernaTatjana Volkov-HusovicIvana Škugor RončevićTatyana N. AlexandrovaIvana SutejTatyana Sergeevna GlaznevaIvar ZekkerTatyana V. VolkovaIvarne L. S. TersariolTaufiq AhmadIvaylo DimitrovTaufiq HidayatIveta CabalovaTawakol A. EnabIvica AvianiTawfik KhattabIvica BokoTawheed HashemIvica GarašićTayfun UygunogluIvica PelivanTayyab NaqashIvo StachivTaza GulIvo ŠulákTe Chuan LeeIvona ČerničkováTeddy Weifa YangIvor LoncaricTedeusz SałacińskiIwan DarmadiTeewara SuwanIwan PrasetiyoTehmina AyubIwona BryndalTei-Chen ChenIwona KarbownikTeik-Cheng LimIwona LazarTej SinghIwona WilińskaTejas BarotIwona ZarzykaTelegdi JuditIyan SopyanTeng Wan DungIzabela Barszczewska-RybarekTengfei QiuIzabela JasińskaTeodolito Guillén-GirónIzabela JendrzejewskaTeoh Seong LinIzabela KruszelnicaTerence A. ImberyIzabela Kuryliszyn-KudelskaTerence LangdonIzabela MajorTeresa AdityaIzabela MatułaTeresa BajorIzabela MiturskaTeresa BravoIzabela ŚliwaTeresa Fina MastropietroIzabell CraciunescuTeresa PalomarIztok PerušTeresa PiJ. KumaraswamyTeresa Pineda RodríguezJ. Marcinko JosephTeresa RucinskaJ. Ramon Gaxiola-CamachoTeresa RussoJ. Ricardo Mejía-SalazarTeresa ZychJabar Jamiu MosebolatanTero FrondeliusJabbar Ali ZakeriTero TuovinenJacek AbramczykTetsuo SogaJacek Adam SorokaTetsuo YamaguchiJacek AndrzejewskiTetsuya KaiJacek CabanTetsuya OzekiJacek FilipeckiTeuku Budi AuliaJacek GołaszewskiTevfik KucukomerogluJacek GóraThabo FalayiJacek GórkaThais AlvesJacek HoffmanThais Suzane Milessi EstevesJacek HulimkaThanasis D. PapathanasiouJacek KasperskiThanate RatanawilaiJacek KatzerThanayut KaewmarayaJacek KrawczykThang Phan NguyenJacek LeśnikowskiThangaraj MuthuramalingamJacek ŁubińskiThangavelu KokulnathanJacek MatysThanh Binh Nguyen ThiJacek MichalakThanh Luan PhanJacek MuchaThanh-Canh HuynhJacek NowaczykThanh-Phong DaoJacek PezdaThanongsak ImjaiJacek RóżańskiThar M. AlbarodyJacek SawickiTharaka Rama Krishna C. DoddapaneniJacek SiódmiakTharmalingam SivarupanJacek SłaniaThemis MatsoukasJacek SzerThenmozhi RajagopalJacek SzmaglinskiTheodor DollJacek TrzaskaTheodoros A. ChrysanidisJacinto CanivellTheodoros KarakasidisJacob HorwitzTheodoros ManourasJacopo FiocchiTheodoros N. KapetanakisJacopo Pedrini Thi Thu LeJacques CurélyThi Tuong Vy PhanJacques Romain NjimouThiago A. CostaJadranka MilikićThiago De M. LimaJadwiga SołoduchoThibaut De RességuierJae Ho KimThierry MaffeisJae Hyuk LimThirumal RajJae Kon KimThirumala Rao GurugubelliJae Nam KimThomas AntretterJae Wung BaeThomas BräunigerJae Yong KimThomas Cai Wei TanJaeha LeeThomas ChristopoulosJaeheum YeonThomas DippongJaehong ShinThomas EchterhofJaemin JeongThomas GerekeJae-Woong YuThomas Hérisson De BeauvoirJaeyoung KimThomas HoferJafar Jafari-AslThomas J. KolibabaJaffar Syed Mohamed AliThomas KlinkeJagadeeswara Reddy VennapusaThomas R. JackJaganathan JayaprakashThomas SchnabelJagannath GangareddyThomas SchweizerJagar A. AliThomas ThiebaultJagoda LitowczenkoThomas VolkmannJahangir MirzaThomas WaltherJahidul Islam ShohagThuat TrinhJai Won ByeonThye Foo ChooJaideep KaturiTiago A. FernandesJaime A. Fernández-GómezTiago Almeida SilvaJaime Andrés Pérez TabordaTiago CruzJaime EspinoTiago Jonatan GirelliJaime Santoyo-SalazarTiago José ArrudaJaime Taha-TijerinaTiago L. P. GalvãoJaime ViegasTiago Vieira Da CunhaJainagesh SekharTian LiuJairo AndradeTian WangJaka BurjaTianfeng YuanJake BairTianhao WangJaksada ThumrongvutTianhao YuJakub BarbaszTianlong ZhangJakub CieslakTianshi WangJakub FitasTianshou MaJakub GrajewskiTianshu LiJakub HlostaTiantang YuJakub KawalkoTianyi LiJakub Krzysztof GrabskiTianyu YanJakub MatusiakTianyuan ZhangJakub VeverkaTiberiu RomanJalal DelaramTibor BedoJalal IsaadTibor KrenickyJalal TorabiTibor KvačkajJalumedi BabuTibor Toth-KatonaJamal KazmiTie YangJamal KhatibTiecheng LuJamal M. RzaijTiejun ChunJamal MabroukiTielong ShenJamasp JhabvalaTihomir DokšanovićJames Bernard MetsonTihomir DovramadjievJames ChowTill LeissnerJames E. KrzanowskiTilo ZienertJames G. LongstaffeTimothy D. VadenJames Grant-JacobTimur V. TropinJames M. GardnerTing HanJames McGregorTing TsuiJames RenTing ZhangJames Robert ManuelTing-Hsun LanJamie A. StullTio WendariJamieson BrechtlTitus SanduJamleh AhmadTiwen LuJan BaeyensToan DinhJan BrodaTobias EisenmannJan ČermákTobias FeyJan Christoph ZargesTobias TauböckJan CoenenTodor G. PenyashkiJan CzyzewskiTohid Ghanbari GhazijahaniJan EdelmannTohid Mirzababaie MostofiJan FořtTo-Hung TsuiJan GóreckiTom WilsonJán IždinskýTomáš BakalárJan KoplíkTomas CeganJan KotekTomáš DlouhýJan KrmelaTomas FilaJan KubicaTomáš KrejčíJan KudlačekTomas KubartJan LatalTomás Madera-SantanaJan MacutkevicTomáš MánikJan MajerníkTomáš ProšekJán MoravecTomas TamulevičiusJan PaczesnyTomas TorrobaJan PapugaTomáš VlachJan PawlikTomáš ŽižlavskýJán PiteľTomaso VairoJán SlotaTomasz BartkowiakJan ŠubrtTomasz BieniekJan SuchorzewskiTomasz BłachowiczJan ŚwierczekTomasz BrylewskiJan SýkoraTomasz BulzakJan TrejbalTomasz CetnerJan TurantTomasz ChmielewskiJan ValentinTomasz CyganJán ViňášTomasz CzeppeJan VrestalTomasz CzujkoJan VychytilTomasz DrzymałaJan ZawalaTomasz DylJan ZbojovskyTomasz GancarzJan ZidekTomasz GawendaJana BidulskáTomasz GorzelańczykJana BoháčováTomasz JankowiakJana HorakovaTomasz JaśniokJana HubálkováTomasz KalakJana Kalbacova VejpravováTomasz KaniaJana ŠmilauerováTomasz Karol PietrzakJana ŠtěpanovskáTomasz KlekielJana TimmTomasz KloskowskiJanardhan KoduruTomasz KrystofiakJandeep SinghTomasz LipińskiJang Myoun KoTomasz ŁodygowskiJang-Hyun LeeTomasz MajewskiJanina AdamusTomasz MazurJanis VirbulisTomasz Norbert KołtunowiczJanna ShainskyTomasz PrzybylińskiJános KissTomasz RozwadowskiJanos LukacsTomasz RybickiJanuar Parlaungan SiregarTomasz ŚlebodaJanusz AdamiecTomasz SochaJanusz JacakTomasz SterzyńskiJanusz JaneczekTomasz SzymborskiJanusz KluczynskiTomasz TomaszewskiJanusz KonkolTomasz TraczJanusz KozakTomasz TrzepiecinskiJanusz KozakiewiczTomasz WasilewskiJanusz KrawczykTomasz WejrzanowskiJanusz Ryszard KrentowskiTomasz WróbelJanusz SikoraTomasz ŻelazińskiJanusz SmulkoTomaž BrajlihJanusz TomczakTomaz PepelnjakJanusz ZmywaczykTomi T. LiJanuszewicz KatarzynaTomica HrenarJanvit TerzanTomislav BituhJarmila TrpčevskaTomislav SedlarJarnail SinghTomoaki KashiwaoJaromir DrapalaTomohiro HigashiJaromír KopečekTomohisa TakayaJaroslav BucharTomoo NakaiJaroslav ČechTomoya NagiraJaroslav HornakTomoya NishiwakiJaroslav JerzTong DengJaroslav KováčTong FeiJaroslav KovacikTong LiuJaroslav OdrobiňákTong ShenJaroslav PokornýTongde ShenJaroslav SmutnýTongsai JamnongkanJarosław BartnickiTongtong XuanJarosław ChojnackiTongwei ZhangJaroslaw GalkiewiczTongyan PanJaroslaw Jan JasinskiTonica BončinaJaroslaw KoniorTonino TrainiJaroslaw KrzywanskiTonni KurniawanJarosław MichałekToomas RangJarosław RomańskiToru NagaokaJarosław RybakToshihiro KanekoJaroslaw SerafinToshihiro KushibikiJarosław SzlugajToshihiro ShimadaJarosław TokarczykToshimasa YoshiieJarosław ŻmudzkiToshio OgawaJarosław ZubrzyckiToshiro YamanakaJarugu Narasimha MoorthyToshiyuki ItohJasim SalmanToshiyuki KanakuboJasmina Grbovic NovakovicTóth LászlóJasmina ObhođašTousif AhmedJasmine SinhaTowhidur RazzakJasna Simonović RadosavljevićToyanath JoshiJason HoffmanTraian MaziluJason LiTraian ZaharescuJason OngpengTraianos V. YioultsisJassim HmoodTran VinhJatinder SinghTré Raymond WelchJavad PayandehpeymanTrevor C. BrownJavad TavakoliTri Ho Minh LeJavaid ButtTri Indah WinarniJaved AhmadTrif MonicaJaved Ahmed NaqashTrinidad Méndez-MoralesJaved IqbalTriratna MuneshwarJavier HidalgoTroy AnsellJavier IñañezTroy R. MunroJavier MartínTroy Y. AnsellJavier Pereiro BarcelóTrushit UpadhyayaJavier PinillaTs. RadevaJavier Ruiz MartínezTsanka D. DikovaJavier S. Blázquez GámezTsanka DikovaJavier TroyanoTse-Lun ChenJavier W. SignorelliTshimangadzo MunondeJaviera ToledoTsung-Rong KuoJawad MirzaTsutomu ItoJay AiraoTsz Him ChowJay J. VoraTuan Anh NguyenJay Prakash VermaTuan Anh PhamJayachandran JayakumarTuan Hiep TranJayakrishna KandasamyTuan Sang TranJayaraman Sethuraman SudarsanTuan ZaharinieJayaraman TheerthagiriTuck Wah NgJayasmita JanaTudor Claudiu SpînuJayavelu Udaya PrakashTuerdimaimaiti AbudulaJea Seung RohTuhin MukherjeeJean Aime MbeyTullio MonettaJean Benoît Le CamTünde Anna KovácsJean Bernard VogtTünde KovácsJean Charles MajestéTung Lik LeeJean Christophe PainTurgay ÇoşgunJean EbothéTurki BakhshJean Marc TullianiTushar Kanti DasJean Marie DrezetTze Kiat LaiJean Marie RuysschaertTze Ning HiewJean Michel RomanoTz-Feng LinJean Paul ChopartTzonka MinevaJean Paul MosnierTzu-Ho WuJean Paulo Dos Santos CarvalhoTzu-Sen YangJean Philippe MonchouxTzu-Yen HuangJean Pierre BellotU. Mohammed IqbalJean Yves DubozUbaidillah UbaidillahJean-Denis ParisseUdaya Bhat K.Jebamaniraj JeyanthiUddhab ChaulagainJȩdrzej WalkowiakUģis CābulisJee Hyun KangUgo CarusoJéferson Aparecido MoretoUgo CovaniJeff MaUgo LafontJefferson AraujoUgur KokluJefferson Jefferson AndrewUlas YamanJeffrey B. AllenUlderico SantamariaJeffrey BullardUlf BetkeJelena B. BajatUli LohbauerJelena D. RusmirovicUlises Páramo-GarcíaJelena GulicovskiUlphard Thoden Van VelzenJelena T. PetrovićUlrike KuhlmannJelena TamulieneUmapathi ReddicherlaJelena ZagoracUmberto De MaioJelka GerśakUmberto TarantinoJen Chang YangUmer Masood ChaudryJen Ray ChangUnbong BaekJennifer GottfriedUndrakh MishigdorzhiynJennifer PattersonUn-Hyuck KimJens NajorkaUnnar ArnaldsJeong Soo SohnUpendra KumarJeong Wan JoUpendra RajakJeong Won ChoiUriel ZapataJeongguk KimUros JosicJeonghoon LeeUroš NovakJeonghwan KimUrszula MizerskaJeongsoo NamUsama HumayounJeongwon YoonUsama UmerJeotikanta MohapatraÜsame Ali UscaJeremie D. OliverUsman AliJerico Bello BelloUsman ZubairJernej KlemencUthayakumar MarimuthuJérôme Polesel-MarisUttam BhandariJérôme Tchoufang TchuindjangUzair SajjadJersson PlacidoV. BharathJerzy BobińskiV. G. EfremenkoJerzy BochenV.L. MangeshJerzy BochniaVáclav PíštěkJerzy CzmochowskiVaclav PrajzlerJerzy GorausVáclav RancJerzy MalachowskiVadapalli SrinivasJerzy NiagajVadim A. ParfenovJerzy SanetraVadim V. PotapovJerzy SmardzewskiVagelis PlevrisJerzy TrzcinskiVahab SarfaraziJerzy WinczekVahed GhiasiJesper T.N. KnijnenburgVahid Najafi Moghaddam GilaniJesse RumboVaibhav KathavateJesse T. AultVaidas LukoševičiusJesús Alejandro Prieto-AmparánVaidotas KazukauskasJesús AtenciaVairavel ParimelazhaganJesus Baldenebro-LopezVaishak ViswanathanJesus Del HoyoVaishakh NairJesús De-Prado-GilVakayil PraveenJesus E. Velazquez-PerezValentin OleksikJesús Fernández RuizValentin RomanovskiJesús Fernando López-PeralesValentina BeghettoJesus Gonzalez-TrejoValentina BertanaJesús Hernández SazValentina CollaJesús Madrigal-MelchorValentina DincaJesús Mena-ÁlvarezValentina DomeniciJesus PernasValentina GucciniJesús Puente-CórdovaValentina LoganinaJesús S. GonzálezValentina MarascuJesús Sergio Artal-SevilValentina MazzantiJesús-María García-MartínezValentina MussiJeyaseelan ChandradassValentina Pavlovna ZverevaJeyashelly AndasValentina SantoroJhe Yu LinValentina SessiniJhon J. IpusValentina UtochnikovaJhonny Villarroel RochaValentina V. ChebodaevaJi HeValentina VassalloJi Won ParkValentina ZambranoJia Geng BoonValentina ZhurikhinaJia Yue YangValentino SangiorgioJiacan SuValeri MourzenkoJiafei ZhaoValeria DemontisJiahao HuangValeria GabrielliJiahao YanValeria PalachevaJiahui ChenValeria RizzoJiahui QiValerii A. BarbashJiali YuValerio Flavio GiliJialong WenValerio GrazianiJiamin WuValeriy E. ArkhincheevJian ChengValeriy V. MaslennikovJian hua ZhaoValeriy VerchenkoJian LuoValery A SvetlichnyiJian QiValery E. TarabankoJian ShaoValery P KrivobokovJian WuValery SinditskiiJian XuValery Stanislavovich LesovikJian YangValeryia KasnerykJian ZhangValiollah PalangiJian Zhang ChenValter Mariani PrimianiJian ZhouVamsi BorraJianbang GeVamsi Nagaraju ThotakuraJianchao PangVan Phuc LeJiancheng LaiVan Thanh NguyenJiancheng LuoVan Thao LeJiandong YaoVan Toan NguyenJianfei WangVan Trinh PhamJianfeng WenVan-An DuongJiang LiVan-Canh TongJiang XiVan-Chuc NguyenJianguang LiuVandilson Pinheiro RodriguesJianguo HuVandna SharmaJianhua ZhangVan-Duong DaoJianjun ShaVanesa BañoJianlei WangVanessa AntunesJianlin SunVanessa MaybeckJianqi QiVanessa ProustJianqiang LiVan-Huy NguyenJianqiang LiuVanina RecoulesJiantang JiangVarsha SrivastavaJianwen LiangVarun S RidharJianwen PanVarun Shenoy GangoliJianwen YuVarvara SygouniJianxin LuVasa RadonicJianxing LiuVasanth Chakravarthy ShunmugasamyJianxing MaoVasil SimeonovJianxiu WangVasile CojocaruJianxun SongVasile Dănuț LeordeanJianxun ZhangVasile George CioatǎJianyu ZhangVasile SimulescuJianyun ShenVasile-Adrian SurduJianzhuang XiaoVasilica PopescuJiapeng SunVasilica ȚucureanuJiaqi ZhuVasiliki KamperidouJiaqian QinVasilina LapitskayaJiaqiao ZhangVasilis DimitriouJiaqing WangVasily A. MilyutinJiawei XiangVasily EfremenkoJiawen GuoVassil JivkovJiawen RenVassilios Ioannou-SougleridisJiawen YongVassilis PontikisJiayu SunVasudev BallalJichao SunVasyl LozynskyiJidong KangVasyl M. HaramusJie GaoVasyl TeslyukJie HuVed Prakash SinghJie LiVeera KrasnenkoJie LinVeera Manohara Reddy YenuguJie RenVeerakumar ArumugamJie XiaoVeerakumar ChinnasamyJie XuVeerendrakumar C. KhedJie YangVeerpal Pal Singh AwanaJie ZhangVeeturi SrinivasJiehua LiVelaphi C. ThipeJieli DuanVelmurugan RamanJierui YuVenerando PistaràJifeng GaoVenkata ChevaliJiheng LiVenkata Krishna Karthik TangiralaJiheon JunVenkata MentaJijia XieVenkatachalam GopalanJijin XuVenkataraman SekkarJijo LukoseVenkatesh VijayaraghavanJilai WangVera AmicarelliJimenez Martinez MoisesVera GuduricJimmie MillerVerica PavlicJin Cherng HsuVeronica MercuțJin HeVeronica PaltaneaJin LiVeronika PavliňákováJin NiuVesna RadojevićJin WangVesna S. CvetkovićJin XuViacheslav RudkoJin YanViatcheslav M. SilkinJin YangVibhor KumarJin Yoo SuhVicente Climente-AlarconJin ZhouVicente Faus MatosesJinbao ZhangVicente Flores-AlésJincan ZhangVicente S. Fuertes-MiquelJinchen FanVicente VentrellaJincy Parayangattil JyothibasuVicky KumarJinfeng HuangVictor A. RodriguezJing Chie LinVictor AtuchinJing GuoVictor Baltazar-HernandezJing HanVictor CardenesJing Li (China)Victor Dmitrievich LakhnoJing Li (USA)Victor EremeyevJing Liu (Canada)Víctor Guillermo Jiménez QueroJing Liu (China)Víctor Javier Cruz-DelgadoJing MaoVictor KarpovJing QuVictor KomarovJing Zhou (University of Science Victor KornevJing Zhou (Xi’an University Victor KuzmichevJingan LiVictor LecaJingcai ChengVictor LisitsynJingen GaoVictor M. Lopez-HirataJingfang LiVíctor Manuel Pérez PuyanaJingjiang QiuVictor NazarychevJingjie ZhangVíctor OestreicherJingkun YuVictor ShapovalovJingquan HanVictor V. TcherdyntsevJingtao BiVictoria CorregidorJingxi LiuVictor-Vlad CostanJingyi WangVidya Nand SinghJingyu WangVieira Estéfano AparecidoJingyuan ZhouViera KučerováJin-Ho LeeViera ZatkalíkováJinjun GuoViet Nguyen HoangJinjun XuViet-Anh HAJinlong LiuViet-Duc LeJinping SuoVigneshwaran ShanmugamJinshan GuoVijay Bhooshan KumarJinshan YangVijay Joseph SanthiyaguJinsheng LuVijay MeenaJinsung AnVijay Raj SinghJinu Jacob GeorgeVijay ShridharJinxin GuoVijay TambrallimathJinxu LiVijay VelisojuJinxuan BaiVijay VyasJinyang XuVijaya Gopalan SreeJinze XuVijayalakshmi ShankarJiong ZhouVijayan DharanendranJiping ChengVijayan KrishnarajJiqin ZhuVijayanandh RajaJiratchaya AyawannaVi-Khanh TruongJiří A. MaresVikram SarpeJiří BrožovskýVikram V. DabhadeJiří CapekVikram VishalJiři ErhartViktor GribniakJiří FriesViktor KharinJiri HouskaViktor LapshinJiří JandaViktor P. AstakhovJiri MajznerViktor SavovJiří MatejíčekViktor V. KlimovJiří PazderkaViktoria NelyubovaJiri SobotkaViktoria RajtukovaJiri SvobodaViktoriya KonovalovaJiří ZýkaViktors HaritonovsJiro KitagawaVilma KaškonienėJitang FanVilma PetkovaJitendra Kumar KatiyarVilma RatautaiteJitendra S RathoreVimala P.Jitesh BarmanVinay AgrawalJithin VishnuVinay Bhushan ChauhanJizhe CaiVinay ChauhanJoachim BaumeisterVinay Kumar JadounJoachim HausmannVinay Singh ChauhanJoan LlorensVinaya Kumar K. B.Joan Manuel Rodríguez-DíazVinayaka RamJoan SunyolVincent BuhagiarJoana AntunesVincent ChanJoana Faria MarquesVincent JiJoana MaiaVincent P.Joana PintoVincentius Surya Kurnia AdiJoana Vaz PintoVincenzo BellantoneJoan-Josep SuñolVincenzo CaligiuriJoanna BorowiecVincenzo FioreJoanna BrzeskaVincenzo MoramarcoJoanna BzówkaVincenzo NaddeoJoanna DobrzańskaVincenzo Romano MarrazzoJoanna Kacprzyńska-GołackaVincenzo TebaldoJoanna KazimierowiczVineet KumarJoanna KolmasVineet Kumar RaiJoanna KończykVineet SrivastavaJoanna Le Thanh-BlicharzVineeth Chandran SujaJoanna LiszkowskaVineeth M. VijayanJoanna Mastalska-PopławskaVinh Tung LeJoanna MichalowskaVinit Kumar GunjanJoanna MystkowskaVinod AyyappanJoanna PagaczVinod KadamJoanna RaczkowskaVinod KumarJoanna RydzVinod KushvahaJoanna RyszkowskaVinod SureshJoanna ZdunekVinod VellorathekkaepadilJoanna ZemłaVioleta KolevaJoao Adriano RossignoloVioleta MelinteJoao BotelhoVioleta NiculescuJoão C.B. MoraesVioleta RamosJoão CaramêsVioleta Valentina MerieJoão CruchoVioletta KytinouJoao Francisco SilvaViorel CircuJoão M. P. Q. DelgadoViorel PaleuJoão M. SantosViorel PaunoiuJoão Manuel Mendes CaramesViorica ParvulescuJoão Nuno PachecoVirendra KumarJoão P. MagrinhoVirendra YadavJoão P.M. PraganaVirendrakumar DeonikarJoão Paulo Costa Do NascimentoVirgil FlorescuJoão Paulo Mendes TribstVirgil OptasanuJoão Paulo TribstVirgilijus ValeikaJoão Paulo WiniarskiVirgilio GenovaJoão Pedro MendonçaVirgiliu S. FireţeanuJoão Pedro OliveiraVirginia Moreno-GarcíaJoão Pinto CoelhoVirginija JankauskaiteJoão RibeiroVirginijus BukauskasJoão SilvaVisan Anita IoanaJoao SilveiraVishal WankhedeJoaquim C. G. Esteves Da SilvaVishnu SaseendranJoaquín Hernández-FernándezViswanath ChinthapentaJoaquin SanchizViswanath DasJobin VargheseViswanathan SajiJochen VogtVit ProchazkaJoel LiebmanVit SmilauerJoerg FroemelVita RudovicaJoerg HildebrandVitali PodgurskiJoey GriffithsVitalii IvanovJoffrey ViguierVitalii Vladimirovich SavinkinJoginder SinghVitaliy B. MykhaylykJohanan Christian PrasanaVitaliy BilovolJohann MartonVitaliy G. ShevchenkoJohann MogeritschVitaly A. MorozovJohann PetitVitaly S. SokolovskyJohanna Karina Solano MezaVitaly TseluikinJohannes LützenkirchenVito GiganteJohannes SchenkVitor AnesJohannes TrappVítor AntunesJohn Anthuvan RajeshVitor CarneiroJohn BellVitor Santos DuarteJohn BolanderVittoria PerrottiJohn BrentonVittorio ChecchiJohn CampbellVittorio Di CoccoJohn Christopher PlummerVivek BajpaiJohn CoronadoVivek NarisettyJohn D. ClaytonVivek PatelJohn D. KechagiasVivek SaraswatJohn G. FisherVivek TrivediJohn George HardyVjaceslavs LapkovskisJohn HalloranVjacheslav ZuevJohn J. S. DilipVladimir A. AseevJohn KinuthiaVladimir A. KomornikovJohn ManderVladimir A. SkripnyakJohn StraubeVladimir ChernyshevJohn VakrosVladimir ChirvonyJohn W. NicholsonVladimír ChmelkoJohnson V. JohnVladimir DodevskiJoji OkazakiVladimir FalkoJolanta BiegańskaVladimir I. ZverevJolanta DzwierzynskaVladimir K. IvanovJolita KruopienėVladimir KolesovJon Ander Onrubia CalvoVladimir KurbatovJon GutierrezVladimir M. MirskyJon Mikel SanchezVladimir MilovanovicJon Tomas GudmundssonVladimir MityushevJonas AlexandreVladimir N. PopokJonas DeuermeierVladimir NeplokhJonas ŠaparauskasVladimir OsipovJonathan BrittonVladimir P ZhdanovJonathan CastilloVladimir PankratovJonathan FilippiVladimir PopovJong Hyeon LeeVladimir ReshetovJong Jeong SoonVladimir ReukovJong Min KimVladimir ShnitovJong Seok ParkVladimir SkripnyakJoon Ho SeoVladimir TsepelevJoon Sung ChoiVladimir UrbanovichJoonghan ShinVladimir VolodinJoonmo ChoungVladimir YussupovJordan MaximovVladimir ZverevJorddy Neves CruzVladimir-Lucian EneJordi BellaVladislav KuchanskyyJordi SuñéVladislav LvovJörg FritzVladislav SemakJörg LäugerVladislav V. GurzhiyJörg MeisterVlasoula BekiariJörg PezoldtVlassios LikodimosJorge AncheytaVlasta Mohaček-GroševJorge B. SousaVlastimil BílekJorge BalmasedaVlastimil BorůvkaJorge BonhommeVlastimil MatejecJorge EscorihuelaVlastimil MatějkaJorge GosalbezVlastimil VodarekJorge López CuevasVojtech VaclavikJorge Luis Costafreda MustelierVolf LeshchynskyJorge Luís Victória BarbosaVolkan AltunalJorge N.R. MartinsVolker AltstaedtJorge PadrãoVolker CimallaJorge Suárez-MacíasVolker KörstgensJosé A. MartinsVolker SchoeppnerJosé A. Siqueira DiasVolkmar StenzelJosé Agustín Tapia HernándezVolodymyr HutsaylyukJosé AlvarezVolodymyr KorolovychJosé Antonio Luceño SánchezVolodymyr KulykJosé António SimõesVolovik GrigoryJose Antonio TenorioVsevolod MymrinJosé BuenoVu Đuc ChinhJosé Campos MartínezVu Khac Hoang BuiJosé Carlos PereiraVukicevic NatasaJose CornejoVyacheslav A. ArlyapovJose De Jesus Agustin Flores CuautleVyacheslav S. ProtsenkoJosé Duarte MarafonaVyacheslav SilkinJose FernandezVytautas JūrėnasJose Florentino Álvarez-AntolínW. Dieter EngelJosé GarciaWacław BrachaczekJosé Gilberto Dalfré FilhoWacław MakowskiJose Gouveia HenriquesWael A. AltabeyJosé Guadalupe ChacónWagner TenfenJosé Guadalupe Rutiaga QuiñonesWahid FerdousJose GuevaraWajid RehmanJose I. PradoWaldemar Andrzej TrzcińskiJosé Jaime Baldoví JachánWaldemar PichórJosé Jaime Taha-TijerinaWaldemar PydaJosé Javier Relinque MadroñalWaldemar StamporJose L. ConchaWaldemar ŚwiderskiJosé Luis Costa-Krämer Waldemar WodziakJosé Luis García CalvoWaldemar WołczyńskiJosé Luis Huertas TalónWaleed AbdelhamedJosé Luis Rivera-ArmentaWalid Al-zordkJosé Luis Rodríguez GutiérrezWalid DebouchaJosé Luis Sánchez LlamazaresWalid MaherziJosé M. ArandesWalid OueslatiJosé Manuel Cardoso XavierWalter CanonJose María FernandezWalter CaseriJosé María Gómez Walter Martin HolwegerJosé María Martínez GonzálezWan Hazman DanialJosé Marie Lopez CuestaWan Inn GohJosé Martínez LilloWan Jefrey BasirunJose MathewWan Maryam Wan Ahmad KamilJosé NevesWan Mastura Wan IbrahimJosé Pedro RinoWan Nor Roslam Wan IsahakJosé R. Reyes-AyonaWangmin MinJosé Ramón Gaxiola-CamachoWanhao CaiJosé Reyes GasgaWanheng LuJosé Rojas SolaWanlop HarnnarongchaiJose Sanchez RamosWanping ChenJose Sebastián Velázquez BlázquezWanxi PengJose TahaWaqas AhmadJosé ValdezWaqas BangyalJose Vicente Ríos-SantosWaqas MuhammadJosé XavierWaqas RafiqJosé Ygnacio PastorWardana SaputraJosef JůzaWaris KhanJosef ProstWaseem AbbassJosef StráskýWaseem AminJosep Maria GuilemanyWaseem Sharaf SaeedJoseph AssaadWasiq UllahJoseph D. HoffmanWataru NakaoJoseph Mwiti MaranguWatit PakdeeJoseph P. HornakWebbe Wei-Po LaiJoseph RizzutoWei CaoJoseph ThrasherWei Dan DingJosés Serafín MoyaWei FeiJoshua F. EinsleWei GuoJoshua IslandWei He (China)Josiel Martins CostaWei He (USA)Josip BrnicWei JiangJosip KranjčićWei LiJosy Anteveli OsajimaWei Liu (Harbin Institute of Technology)Josy OsajimaWei Liu (University of Science Jovan CiganovicWei Long NgJovan NedeljkovicWei TangJovana FrancuzWei Wang (Australia)Jovana JuloskiWei Wang (Italy)Jovana NikolovWei Wang (National University Jovica SokolovićWei Wang (Peng Cheng Laboratory)Joyanta ChoudhuryWei WenJoydip GhoshWei WuJoyjit KunduWei XuJoyraj ChakrabortyWei YangJózef HaponiukWei ZhangJózef HorabikWei Zhao (Changshu Institute Jozef IwaszkoWei Zhao (Xi’an Jiaotong University)Jozef JurkoWei ZhouJozef KuczmaszewskiWeichao YanJozef KúdelčíkWei-Chun LinJozef KulkaWei-Chung YeihJozef MajerikWeidi LiuJozef MitterpachWeidong ZhuangJozef PeterkaWeifeng LinJózef T. HaponiukWei-Feng SunJuan Adrián Ramos GuivarWeigang ZhangJuan BoschWei-Hao LeeJuan C. Olivares-GalvanWeihao YuanJuan Carlos Cabanelas ValcárcelWeihong GaoJuan Carlos Durán-ÁlvarezWei-Liang QianJuan Carlos González De SandeWeilue HeJuan Carlos MatosWeimin Chen (Chongqing University)Juan Carlos Pereira FalconWeimin Chen (Jinan University)Juan Carlos Rendón-AngelesWeimin HuangJuan Carlos Suarez BermejoWeimin LiJuan Felipe BeltranWeina HanJuan Ignacio Ahuir-TorresWei-Ping HuJuan J. GaiteroWeiqi HuaJuan José Benvenuta-TapiaWei-qiang PangJuan Jose Galán-DíazWei-Qiang SunJuan José López-CelaWeiqun LiuJuan José Santana RodríguezWei-Ting LinJuan Lizarazo-MarriagaWeiwang WangJuan Manuel Rodríguez PrietoWeiwei LiuJuan María Terrones-SaetaWeixiong LiangJuan P. Palomares-BáezWeiyao ZhaoJuan Pablo Loyola-RodríguezWeizhao ZhangJuan Pedro FernándezWeizhong HanJuan PouWellington MazerJuan Rubio AlonsoWen Lin ChaiJuan TorninWen Luo (China)Juan Vielma PerezWen Luo (USA)Juarez Benigno PaesWen ZhangJudit TelegdiWenbin FeiJue LiWenbin ZhouJui Pin HungWenbo HanJui Yung ChangWenbo LiuJulfikar HaiderWen-Chen HuangJulia Contreras GarciaWenchen MaJulia D. Toscano GaribayWen-Cheng ChenJulia Guerrero GironésWenfeng HaoJulian ChojnowskiWeng Hoe LamJulian FriebelWengang HuJuliana AnggonoWengang ZhangJuliana FernandesWenhao LiJulien LangWen-How LanJulien O. FadonougboWen-Jauh ChenJulija VolmajerWen-Jen LeeJulio C. Rosas CaroWenjian ZhengJulio C. Velázquez-AltamiranoWenjie ZhouJulio Cesar VelázquezWenjuan SongJulio PinheiroWenjun ZhuJulio Ramirez CastellanosWenliang ZhuJulio Romero NogueraWenlong GaoJuliusz LeszczynskiWenming JiangJuliusz OrlikowskiWensheng LiJun Beom ParkWensheng WangJun ChenWentao HouJun Cong GeWentao LiuJun GaoWen-Ten KuoJun Ichi KadokawaWenwu XuJun Ichi KanekoWenzhou YuJun Jiang (Nanjing Forestry University)Weon Ho ShinJun Jiang (Southwest University Werner TirlerJun Ki AhnWessam H. Abd-elsalamJun LiWibawa Hendra SaputeraJun LiangWidiastuti SetyaningsihJun Liu (UK)Widya FatriasariJun Liu (USA)Widya LestariJun LuoWiesław A. GrabonJun Rui LiangWiesław KacaJun SawaiWiesław UrbaniakJun ShimizuWilfried SigleJun WangWilfried WunderlichJun Young HwangWill GatesJun Young ParkWilley Liew Yun HsienJun ZhaWilliam AperadorJunaid AhmedWilliam ChiappimJunaidi KhotibWilliam DunnJunbo SunWilliam Streit CunninghamJuncheng JinWilliam WaltersJunfeng YangWilliam Y. RaynorJung Jin LeeWillie P. AbasoloJung ParkWilson Merchan-MerchanJung Woo LeemWioletta GrzmilJung Yong KimWioletta RaczkiewiczJung Youl ChoiWirasak SmitthipongJunghong MinWirginia PilarczykJunghwan KimWirginia TomczakJungwon HuhWisena PercekaJungwon KangWislei R. OsórioJunhui XiaoWissam FarhatJunhwa LeeWissem GallalaJunjie HaoWitold KrajewskiJunjie ShiWojciech A. PisarskiJunjie WangWojciech BochnowskiJunkil ParkWojciech BurianJunlei QiWojciech CzekalaJunping DuWojciech DawidowskiJunping ZhangWojciech FabianowskiJunqiang Justin KohWojciech GawlikJunrui LiWojciech GłuszewskiJunsik YoonWojciech KicińskiJunyan WangWojciech KieratJunyan YiWojciech MacekJunye LiWojciech NowakJunyi SunWojciech PawlakJunyu WangWojciech SikorskiJunzhe LouWojciech SimkaJuraj BelanWojciech SochackiJuraj GerliciWojciech SzelągJuraj KralikWojciech TarasiukJuraj ŠrámekWojciech WolanśkiJure ŽigonWojciech Zając (AGH UniversityJurgen FrickWojciech Zając (Henryk Niewodniczanski Jürgen IhlemannWojciech Żórawski (Politechnika Jurgen LangeWojciech Żurowski (Kazimierz Pulaski Jüri LavrentjevWolf-Dieter MuellerJuris BurlakovsWolfgang FrickeJustin W. HendrixWolfgang Gindl-AltmutterJustus AdamsonWolfgang HeringJustyna KujawskaWolfram VolkJustyna ZygmuntowiczWon Chun OhJuveriya ParmarWon Min ParkJuyoung KimWon-Bae ParkJuzer ShabbirWonho LeeJyothsna ManikkathWonjin NaJyoti JaiswalWon-Suck OhJyotirmayee NandaWonsuk ChaJyotirmoy NandyWook Jin LeeJyotishkumar ParameswaranpillaiWook KimK. Hari PrasadWoon Kiow LeeK. I. Syed Ahmed KabeerWorawat WattanathanaK. K. NagarajaWork Galal Abdelrehim RashwanK. Kamala BharathiWorradorn PhairuangK. Lakshmi RojaWu AnhuaK. M. JadhavWu GuangningK. M. Tanvir AhmmedWu WangK. P. JagadeshWu ZengK. PrasannaWufei TangK. S. SenkevichWuyi MingK. VipindasWynand JvdM SteynKaarlo NieminenXabier GómezKadir BilisikXavier BoujuKadir OzaltinXavier CastelKadri Can AtlıXavier ColinKah Meng YamXavier ColomKai Chun LiXi JiangKai DongXia GaoKai HuangXiadong WangKai RenXialu WeiKai SellschoppXian WuKai TreutlerXianchen XuKai WangXiang ChenKai XuXiang LeiKai YaoXiang LiKai ZhangXiang ZhangKai ZhuXiangang LuoKaige WuXiangdan MengKaikai SongXiangdong WuKaiqiang QinXianghe PengKaitak WanXiang-Jun ZouKaixiong GaoXianglei ZhengKaiyang YinXianglong MengKajal GhosalXiangru WeiKajetan DabrowaXiangwei WuKalidass SureshXiangyang DongKalipada BankuraXiangyong NiKalle KirsimäeXiangyu GaoKalyan RameshXiangzhen ZhuKamal I. AlyXiangzhong ChenKamal KumarXiangzhou LaoKamalan Kirubaharan Amirtharaj MosasXianhu LiuKamalesh PasadXianjie LiuKambiz TahvildariXiankui WeiKamel AgrouiXiao ChenKamil DychtońXiao Dong ZhouKamil JurczyszynXiao KuangKamil KaminskiXiao LiuKamil KarimullinXiao ZhaoKamil KaszycaXiao ZhouKamil Krzysztof RomanXiaobin HuKamil KrzywinskiXiaochao TangKamil MajchrowiczXiaocheng LiKamila KociXiaodi WangKamila MaleckaXiaodong PengKamila MizeraXiaodong ShiKamlesh MauryaXiaodong WangKamonpan PengpatXiaodong YangKamonwat NakasonXiaodong ZhangKamran AminiXiaofeng LiuKamran AsemiXiaofeng WuKamran BagheriXiaofeng ZhaoKamrul IslamXiaoguang GuoKamyar Shirvani MoghaddamXiaohong ZhangKana PuspitaXiaohong ZhouKanagaraj PalsamyXiaohong ZhuKanak KalitaXiaojie XuKanchi Balaji RaoXiaojun LiKandasamy SaravanakumarXiaolan WangKandasamy SelvamXiaoliang QiKandpal Bhaskar ChandraXiaolong HaoKang Chiang LiewXiaolong MaKang Seok LeeXiaolong WangKang ZhaoXiaolu PangKangning WuXiaomei HeKannan ChidambaramXiaomei YangKannan SridharanXiaomeng Zhang Kanokwan NgaosuwanXiaomin XuKapil DebnathXiaomin ZhuKapil GangwarXiaoming LiuKapil GuptaXiaoning ZhangKarel CarvaXiaoqiang AnKarel SakslXiaoqin ShenKarem A. MahmoudXiaoqing JiangKaren L. PoseyXiaoqing SiKari MantyjarviXiaoqun ZhuKarim AliakbariXiaosong JiangKarim TanjiXiaosong WangKarl Fredrik NilssonXiaotian WangKarl O. ChristeXiaoting HeKarla J. MorenoXiaotu MaKarla MossiXiaowei ChengKarna WijayaXiaowei GuKaro SedighianiXiaowei YuKarol BulaXiaowei ZengKarol FrydrychXiaoxin XuKarol I. WysokinskiXiaoyan WangKarol JaśkiewiczXiaoyang YiKarol KyziolXiaoyi ZhouKarol LemańskiXiao-Yong WangKarol MiądlickiXiaoyu ChenKarol StrzałkowskiXiao-Yu WuKarol SynoradzkiXiaoyu ZhangKarol SzałowskiXiaoyu ZhaoKarol SzubertXiaoyuan ZhouKarol TuckiXiaozhou JiKarolina ChilickaXijia WuKarolina Goździewska-HarłajczukXilin WangKarolina Kołczyk SiedleckaXin ChenKarolina LabusXin F. TanKarolina Zofia MilowskaXin GuiKaroline FigueiredoXin GuoKarthick RajanXin HeKarthick SubbiahXin Ping WuKarthik Reddy PeddireddyXin Wang (South China Normal University)Karthik V. ShankarXin Wang (University of Karthikeyan SekarXin WuKarthikeyan SubramanianXin YiKartick Kumar SamantaXin ZhaoKaruppasamy KaruppasamyXin ZhuangKaruppasamy Pandian MarimuthuXinbo WangKaruppusamy LoganathanXinchen WuKaruvanthodi MuraleedharanXinfang Zhang (China)Kashif IshfaqXinfang Zhang (USA)Kashif Mairaj DeenXing LuKasin RansikarbumXingang LiuKaspar M.B. JansenXingdong ZhaoKasper EerselsXinglai ZhangKassim S. Al-RubaieXinglin YuKatakam Siva PrasadXinglu WangKatalin KopecskóXingxing JiangKatarina MihajlovskiXingyu WangKatarina MonkovaXingyu ZhangKatarzyna BerentXingyun LiKatarzyna BuczkowskaXinhong WangKatarzyna Cesarz-AndraczkeXinhua YangKatarzyna CieślakXinlin YanKatarzyna FalkowiczXinmin ShenKatarzyna GabryśXinwei LiKatarzyna GasXinyou LiuKatarzyna Gaweł-BębenXinyu LiKatarzyna GurzawskaXinyu LiuKatarzyna KonopkaXinyue WangKatarzyna KrukiewiczXiongfa YangKatarzyna Leszczyńska-SejdaXiubing HuangKatarzyna LewandowskaXiujuan WangKatarzyna NowinskaXiuli HeKatarzyna PentośXiuyan LiKatarzyna RećkoXiuyang FangKatarzyna RutkowskaXu ZhangKatarzyna SkórczewskaXuan GaoKatarzyna StaszakXuan Hung NguyenKatarzyna SzepietowskaXuan LuoKatarzyna WittXuan MeiKaterina E. AifantisXuan Phu DoKaterina KotzampassiXuan ZhuKaterina KreislovaXuanyi XueKaterina MouralovaXubo LuoKateřina OpatováXudong ZuKatharina RichterXue Liu (China)Katharine Moore TibbettsXue Liu (USA)Kathiravan SrinivasanXuedan WuKatica ŠimunovićXuedao ShuKatja VasićXuefeng TangKatja WätzigXuehui AnKatsuhiko InabaXuejiao LiKatsuhiko SakaiXuejuan CaoKatta VenkateshwarluXuenan GuKaupo KukliXueqiang LiKaushik BiswasXuesong LiKaushik PalXuetong ZhaoKaustuv MannaXuewei FangKaveh EdalatiXuewu LiKavimani V.Xuexia HeKavita KumariXueze JinKawachi TetsuyaXuezhi MaKay Peter HoyerXuezhi QinKay SmarslyXun ZhangKayaroganam PalanikumarXusheng DuKazem Reza KashyzadehXusheng YangKazi Abu SohelYa ShenKazimierz FabisiakYachao ZhangKazimierz LatkaYadigar Gülseven SıdırKazuhiko KitamuraYading XuKazuki ImasatoYadollah YaghoubinezhadKazumasa KawasakiYaewon ParkKazunori UmeoYagnesh ShadangiKazutake KomoriYan BeygelzimerKazuya SaitoYan HuangKazuyuki HokamotoYan Jin Lionel LeeKe CaoYan LiKe HanYan SuffrenKe ZhangYan V. ZubavichusKee Hag LeeYan XieKee Sung LeeYan ZhaoKee Young KooYan ZhouKee-Yeon KumYan ZubavichusKefayat UllahYana SuchikovaKegong FangYanbo PanKehuan WangYanchao ZhuKei NishidaYandienka Martinez-RubiKeiichi EdagawaYandong WangKeiji KomatsuYanfang RenKeiji MurayamaYanfei CaoKeisuke MiyazawaYanfei LiuKeivan DavamiYang BaiKeivan KianiYang HuKeiyu KawaaiYang LiKeke SunYang Liu (China)Kelly LangertYang Liu (USA)Kelvin Ian AfrashtehfarYang LuKen IshikawaYang SongKen SuzukiYang Zhang (Denmark)Kengo OkaYang Zhang (Macao)Kenichi MasudaYang ZhaoKening LangYang ZhouKenji HirataYang-Hee KwonKenji ItakaYanghui LiuKenji KanekoYang-Kao WangKenji TairaYanglong LuKenjiro UesugiYang-Tse ChengKennedy Chibuzor OnyeloweYanguang ZhouKenneth ScheplerYangxin YuKensuke OgawaYangzhen LiuKenta AoyagiYanhu ZhangKenta MoritaYanhua ZhaoKerlos Atia AbdalmalakYaniv GelbsteinKerroum DerbalYanjun HanKerstin ElertYan-Jye ShyongKerstin MüllerYanliang HuangKeshavamurthy K.Yanling LiuKęstutis BaltakysYanling SchneiderKeum Hwan ParkYann LanoiseléeKeun Soo KimYannan ZhangKevin PaineYanni BourasKevin PotterYannick BleuKeyang WuYannick ValléeKhader HamdiaYannick VerbelenKhadiga IsmailYanqi WuKhairul Anwar Bin IshakYanshan LouKhairul Fikri Bin TamrinYanshu ShiKhairullo Faizullaevich MakhmudovYan-Ting SunKhairulmazmi AhmadYanwei LiKhaled AlthubeitiYanxiang WangKhaled E. El-KelanyYanxiao LiKhaled GiasinYanxiao NingKhaled O. SebakhyYan-Zhen ZhengKhaleed Abdelsabour ElsayedYanzhong TianKhaleel H. YounisYao FuKhalid AlmasYao LiangKhalid ElwakeelYao MaoKhalid HashimYao ShenKhalid HattarYao SunKhalid MehmoodYao Wang (China)Khalid UmarYao Wang (USA)Khalifa Al KhalifaYao ZhaoKhalil TamersitYaodong LiuKhameel B. MustaphaYaohua WeiKhan NekoueeYaovi GagouKhashayar GhandiYaoyao ChenKhawla S. KhashanYap Soon PohKhem B. ThapaYarub Al-DouriKhian-Hooi ChewYaser HadadianKhoa Tan NguyenYaser KianiKhong Wui GanYashwant Kumar ModiKhrystyna HarhayYashwanth Kumar BandariKhubab ShakerYasin Onuralp ÖzkılıçKhuram MaqsoodYasin OzgurlukKhurram Karim QureshiYasin ÖzkılıçKi Buem KimYasir HaleemKi Hyun NamYasir NawabKi Sub ChoYasser AbdelrhmanKiadtisak SaenboonruangYasser Saleh Mustafa AlajeramiKien TongYassine SlimaniKien Voon KongYasuhiro MatsudaKiho ChoYasuhiro NamuraKimihiro SakagamiYasuko MoriyamaKimihiro YamashitaYasushi NishiiKin LiaoYasushi SasajimaKinga BociongYasushi ShimadaKinga K. TurzóYasutaka AndoKinga KorniejenkoYasutaka KitahamaKinga NalepkaYawar AbbasKiran BholeYean Ling PangKiran Kumar NamalaYeau-Ren JengKiran Raj MelayiYebin XuKiril KrezhovYe-Chan LeeKirill Borisovich LarionovYee Seng TanKirill KhorkovYee SoongKirill M. SkupovYee-wen YenKirill NikitinYen-Fang SuKirill P. BirinYen-Ju ChenKirill V YusenkoYeong Huei LeeKirsi SvedströmYeong-Her WangKirsten J. SchimmelYeong-Joon ParkKirubajiny PasupathyYessica S. Tapia-GuerreroKisan ChhetriYevgen KarpichevKishor Kumar GajraniYevgeniy NaumovichKishore Babu NagumothuYevgeny VapnikKishore BingiYew Hoong WongKishore DebnathYexiang TongKishore Kumar DevarepallyYexiao ChenKishore Kumar S.Yeyoung HaKit Ling ChinYezid A. AlvaradoKit Lun YickYi ChenKittipoom RodsinYi HanKiyofumi KurumisawaYi HuKiyohiro ItoYi HuangKiyokazu YasudaYi SunKiyoshi FujisawaYi WangKlaus BöningYi XuKlaus SzielaskoYi YangKlaus ThieleYi ZhangKlaus VoitYi ZhaoKlemen BohincYichao ZhaoKlodian XhanariYichun LinKnut MarthinsenYifan ZhangKochupurackal B. JineshYifan ZhuKohei NagaiYifei JinKohei ToyodaYifei MaKohei UenoYifeng CaoKoichi ShigenoYifeng WuKoichi ShinkaiYigeng XuKoji MurataYihang FangKok Heng SoonYihu DaiKoki KitagawaYihui DongKokot GrzegorzYihui PanKolan Madhav ReddyYi-Hung LiaoKollarigowda RavichandranYi-Kuo ChangKonda Gokuldoss PrashanthYilin WangKonda Srinivasa RaoYilun XuKongjian ShenYilun ZhuKonidakis IoannisYimin YangKonrad GruszkaYinfei DuKonrad LaberYing Dan LiuKonrad MalickiYing PanKonrad MollenhauerYing SunKonrad SzaciłowskiYing ZhaoKonstantin A. ProsolovYingang GuiKonstantin B. YushkovYingbin WangKonstantin BorodianskiyYing-Guo ZhouKonstantin KudryavtsevYingjie CaiKonstantin NaumenkoYing-Liang ChenKonstantin P. AndryushinYingtao LiuKonstantin P. GaikovichYingxia LiuKonstantin Petrovich SavkinYingya LiuKonstantin S. RodyginYingyi ZhangKonstantin TretiakovYingying WuKonstantina GeorgouliYingzi YangKonstantina TsigkouYining FengKonstantinos BrintakisYintang WenKonstantinos ChrissafisYiqi YehKonstantinos GkyrtisYiran WangKonstantinos KatakalosYiran ZhangKonstantinos MorfidisYiren SunKonstantinos SotiriadisYishuai XuKonstantinos TatsisYitao ChenKonstantinos TsongasYi-Tsung ChangKonstantins JefimovsYiu-lun TangKorakod NusitYiwei WangKornel PietrzakYiwen LiKosar Hama AzizYiwen WangKoshiro NishimuraYixi ZhuangKosmas L. TsakmakidisYixing Wang (China)Kostas KasiotisYixing Wang (USA)Kostas ModisYixuan FengKostiantyn TorokhtiiYlenia MieleKosuke BeppuYoanh Espinosa-AlmeydaKosuke NozakiYogendra MishraKotrechko Sergiy O.Yohei HayashiKouider MadaniYoichi NakajimaKoumei BabaYoichi TanabeKourosh NasrollahzadehYoji HoriiKoutavarapu RavindranadhYoko Yamabe-MitaraiKovářík OndřejYokub ErgashovKozubal JanuszYolun YangKrakowiak StefanYong GengKranthi ManiamYong Gyu ChoiKrisada ChaiyasarnYong Nam AhnKrishanu RoyYong PanKrishna Kiran TalamadupulaYong SangKrishna Murari PandeyYong Soo YoonKrishna Nama ManjunathaYong SunKrishna SharmaYong WangKrishnamurthy PrabakarYong X. GanKrishnan RanganYong XieKrista R. LimmerYong Xu (Germany)Kristin TrommerYong Xu (USA)Kristina RamanauskienėYong Zhang (University of Science Krunoslav JuraicYong Zhang (Yunnan Normal University)Krystian MistewiczYongbo ShaoKrystyna JabłońskaYongfei WangKrzesimir CiuraYonggang MengKrzysztof AniołekYong-Hak LeeKrzysztof Artur BogdanowiczYonghua YanKrzysztof BienkowskiYongjiang HuangKrzysztof BiernackiYongjun WeiKrzysztof BiernatYongjune KimKrzysztof ChwastekYonglan LiuKrzysztof DudzikYong-Moon LeeKrzysztof GorzkiewiczYongqiang JiKrzysztof GrzelakYongqiang WangKrzysztof J. KurzydłowskiYongquan QingKrzysztof JamroziakYongsen YuKrzysztof KruczalaYong-Seok LeeKrzysztof KubiakYongsheng YuKrzysztof KurzydłowskiYong-Sik AhnKrzysztof KusmierekYongxi LiangKrzysztof LukaszkowiczYongxian HuangKrzysztof MazurekYoo Sei ParkKrzysztof MiecznikowskiYordan KyosevKrzysztof MudrykYoshiharu KimuraKrzysztof NowackiYoshiharu MutohKrzysztof PielichowskiYoshihisa MiyataKrzysztof RadwańskiYoshiki TakagiwaKrzysztof Robert CzechYoshiko MiuraKrzysztof RokoszYoshio HondaKrzysztof SchabowiczYoshitaka OkitsuKrzysztof SiczekYoshito IkadaKrzysztof SiemekYosra A. HelmyKrzysztof SkrzypkowskiYosuke GotoKrzysztof TadyszakYotsanan WeerapolKrzysztof WieczorekYoucai ZhaoKrzysztof WilczynskiYoufu ZhouKrzysztof ZabaYoujian YangKrzysztof ŻakYouliang ChengKrzysztof ZboinskiYoun-Bae KangKsenia BelovaYounes AminiKsenija DurgoYounes HanifehpourKseniya A. RomanovaYounes MoussaouiKuan Jen ChenYouness ArjouneKuang Yao LoYoung Sik KimKuang ZhangYoung-Hak LeeKubendran RajkumarYoung-Ho ChoKuldeep MishraYoung-Min KimKulmani MeharYoungmin LeeKulwinder SinghYoung-Ran YooKumar Chandan SrivastavaYoung-Su LeeKumar SadayappanYoussef W. NaguibKumar VikrantYu ChenKumaran KadirgamaYu Chen (China)Kumaran VediappanYu Chen (The Netherlands)Kumi Barimah EricYu CherepennikovKummara Madhusudana RaoYu G BarabanshchikovKun LiYu GongKun LuoYu Jie WeiKun TianYu Kyoung RyuKun YangYu LiangKun ZhengYu QiaoKun ZhouYu SekiguchiKundalwal ShaileshYu-Xin ZhangKundan SivashanmuganYu ZhangKunio HayakawaYu. A. KhonKunlun HongYu. F. ZuevKuntal MajiYu. I. BaumanKuntala BhattacharjeeYu. M. BelousovKuo LiuYu. V. IoniKuo Yung HungYu. V. PisarevskyKurapova OlgaYuan ChenKursat AlyamacYuan QinKürşat ErYuanbiao TanKurt DietlikerYuan-Chang LiangKuruvilla JosephYuanchao PeiKush MehtaYuandong PengKwang S. YoonYuan-Jen ChangKwang Yong ChoiYuanpeng WuKwangeun KimYuansheng ZhouKwok Kan SoYuanwei ZhuKyoung Seok MoonYuanxun WangKyriakos KourousisYuanzhu MiKyuichi YasuiYuanzuo WangKyungil KongYucan PengKyung-Mee ParkYuchao BaiL. Di G. SigalottiYu-Che LinL. KrishnarajYu-Chung ChangL. Pentti KarjalainenYue JiangL. PoovazhaganYue XiaoL’uboslav StrakaYuefeng SongL’udmila DulebováYueh-Lien Robbie LeeLadawan SongtipyaYuesheng LiLadislav CvrčekYueyu TongLadislav HavelaYuezhong WangLadislav ReinprechtYufei ZuLadislav VrsalovićYufeng QinLahcen KhouchafYufeng WangLahiri Barid BaranYugal Kishore MohantaLai ZouYuhang DengLaibao ZhangYuhao Li (China)Laila ZikoYuhao Li (USA)Laisuo SuYuhong ZhaoLaith A. SabriYuhua SuLajos Károly VargaYu-ichi KomizoLajos NagyYuji HamamotoLakshmi Narayan RamasubramanianYuji SasakiLakshmi Priya DattaYujiao LiLakshminarasimhan RajeshkumarYujie SongLakshmiprasad GurralaYuki NagamatsuLaleh YerushalmiYuki NoguchiLalit KumarYukihiro MatsumotoLaly PothanYukinori TaniLamprini MalletzidouYukio TakagiLanh Si HoYukui CaiLapyote PrasittisopinYulei DuLara CostantiniYulia I. DeryabinaLara RebaioliYulia KuznetsovaLara RighiYulia N. BespalkoLarisa A. TsarkovaYulia SpivakLarisa DyachkovaYulia TertyshnayaLarisa TsarkovaYu-Liang ZhangLarissa BernardiYulin HaoLarry PershinYuling YangLars HelbigYuliya NashchekinaLarysa NeduzhaYuliya Yu. TitovaLászló AlmásyYulong HuangLászló JanovákYun ChenLászló NagyYun Jung YangLászló PohlYun ShiLaszlo TothYun ZhengLatefa SailYunchao TangLatifa BouissaneYun-Che WangLaura AcquasalienteYuncong LiLaura BulgariuYunfei ChangLaura Caneda MartínezYun-Fei FuLaura FabbianoYun-Feng XiaoLaura FalchiYung Hsiang ChenLaura GaluppiYung-Jin WengLaura Maria ManceriuYung-Kang ShenLaura MartínYung-Tse HungLaura MorettiYunhyuk ChoiLaura Navarrete AlgabaYuni K. KrisnandiLaura PilozziYunia Dwi RakhmatiaLaura SilvestroYunqiang ZhaoLaura VittadelloYunxiang MaLaurence J. WalshYunyun LiLaurence LeherteYunyun SunLaurent LibessartYupeng LüLaurent RaymondYupiter H.P. ManurungLaurentiu FaraYuqi JinLaurentiu RusenYuqing WuLaurentiu SlatineanuYuqing XingLauro BucioYurdakul AygörmezLavinia ArdeleanYuri A. MeyerLavinia Cosmina ArdeleanYuri A. VorotnikovLaxman Raju ThoutamYuri HovanskiLazar KovačevićYuri KalvachevLe Van SangYuri KonstantinovLe YangYuri RibakovLe YuYuri SharkeevLea ŽibretYuri VoznyakLeandro Alfredo RamajoYurii OrlovskiiLeandro SaccoYurii SharkeevLebon NicolasYuriy G. DenisenkoLech B. DobrzańskiYuriy LittiLech LichołaiYury BelousovLechosław TuzYury KapelyushinLee YounkiYury KistenevLeela Raghava Jaidev ChakkaYury PismakLei BaoYury RyabchikovLei ChangYury V. IlyushinLei HuangYusaku MagariLei Li (China)Yuset Guerra DávilaLei Li (USA)Yu-Sheng SuLei NieYushi LiuLei Wang (Beihang University)Yusuf A. MehtaLei Wang (Xi’an University Yusuf PolatLei ZhaiYusuke HiejimaLei Zhao (China University Yuta MatsushimaLei Zhao (Dalian University of Technology)Yuta TsujiLeidy M. Martínez TejadaYutao PengLeif BulowYutao YanLeily Macedo FiroozmandYuushou NakayamaLeire VirtoYuvaraj NatarajanLeiting ShenYuwei FanLejeunes StéphaneYu-Wei SuLele LuanYuxiao JiangLena Sundqvist ÖqvistYuxin TongLénia M. CaladoYuxin ZuoLenka KvetkováYuyan JiangLenka ScheinherrováYuyan ZhangLeo Alvarado-PereaYuyang DongLeonardo Bertolucci CoelhoYuye TanLeonardo IannucciYuyue QinLeonardo RanaldiYuzhou ZhaoLeonardo Rosa Ribeiro Da SilvaYuzo NakamuraLeonardo Sastoque PinillaYves RoussignéLeonel Paredes-MadridZabiholah ZabihiLeonid A. NikiforovZacharias ViskadourakisLeonid AgureevZafar IqbalLeonid DubrovinskyZafar SaidLeonid FershtatZafer ÇıplakLeonid MarochnikZafer MutluLeonid Moiseevich GurevichZahed AbuLeonid V. KulikZahid HossainLeonidas N. GergidisZahid KhanLeopoldo FornerZahra LotfollahiLesley BergmeierZahra MazroueiLesley CornishZaid Hazim Al-SaffarLeszek BlachaZaida OrtegaLeszek BryjaZaiji ZhanLeszek CzechowskiZainab MahdiLeszek KubiszZaka UllahLeszek ŁatkaZan NieLeszek SzalewskiZan TongLeszek WanatŻaneta Anna MierzejewskaLeszek ZaraskaZaneta SwiatkowskaLev B. ZuevZara BagdasarianLev F. SpevakZarel Valdez-NavaLev RapoportZarina Binti ItamLevan ChkhartishviliŽarko MitićLevent PelitZbigniew BrytanLevent UrtekinZbigniew HnatejkoLeyre CatalanZbigniew KrzemianowskiLi Ching ChangZbigniew LechowiczLi JiangZbigniew NadolnyLi LiuZbigniew PaterLi LuZbigniew PedzichLi QibinZbigniew RaszewskiLi Wei TsengZbigniew SaternusLia RimondiniZbigniew StarowiczLian C. T. ShouteZbigniew StojekLiang FanZbigniew SurmaLiang HongZbynek RaidaLiang ZhangZbyšek PavlíkLiangbo HuZdenek DrozdLiangfeng HuangZdenek RemesLiangjun XiaZe’ev PoratLiangliang WeiZeeshan KhanLiangting SunZeeshan Ur RehmanLiangyun LanZefeng WenLianshe FuZeferino Gamiño-ArroyoLianxiang YangZehra EdisLibing ZhengZeinab Abdel HamidLida HengZeinab Malekshahi BeiranvandLidia BeneaZeki CandanLidia GavicŽelimir JelčićLidija Slemenik PerseZeljka PetrovicLidón Gil EscrigŽeljko AlarLihua WangZeman OliverLijun LiZengbo WangLijun XiaoZenghui DiaoLili HuZenghui LiuLilia CroitorZengping ZhangLiliana Argueta-FigueroaZengsheng MaLiliana BizoZengxia MeiLiliana PorojanZeno ToffanoLiliana Reynoso CuevasZewei YuanLiliya DunyushkinaZeynep Güven ÖzdemirLillian GungatZeyu ZhangLily PoulikakosZhai YueLin ChengZhandos TauanovLin LiZhangjian ZhouLin MuZhanyong ZhaoLin QiuZhanzhan TangLin ZhangZhao DingLina BufalinoZhao ShangLina ZhaoZhaodong XuLinda J. JohnstonZhaogui WangLinda LorenzZhaoming TianLinda PetersZhaoqiang ChuLing SunZhaorong HuangLingbing KongZhaoxi SunLinghang WangZhaoxia RaoLingju MengZhaoye QinLingling XiaZhaozhu ZhengLingling ZhaoZhe KanLingqian ZhangZhe WangLingwei LiZhen Zhang (Huazhong University Lingwei MaZhen Zhang (Tongji University)Lingxiao WangZhenbo LuLingyao MengZhenbo ZhangLinh Van Hong BuiZhendong ShaLinjiang ChaiZheng Ze PanLino BiancoZheng Zhang (China)Linping ZhaoZheng Zhang (USA)Linrong ShiZhengfang YeLinyue TongZhengkai WuLionel DesgrangesZhengkai XuLionel FlandinZhengming HuangLior MedinaZhengran HeLioua KolsiZhengyan YangLiping GuoZhenHua TangLiping LiuZhenhuan YiLippo V. J. LassilaZhenli MiLiqiang WangZhenlin ZhangLiqiu ZhengZhenxin WangLiqun LiuZhenyi ZhangLiqun XuZhenyu LiuLirio SchaefferZhenyu ZhangLisa BiasettoZhenyuan YinLisa BurrisZhexenbek ToktarbayLisandro SimãoZhexi LinLiseane Padilha ThivesZheyu LuoLiseane ThivesZhi Guo LiLisha PengZhi LinLisiane SantanaZhi ZhangLiu KuiZhichao LouLiu ZhengjianZhidan SunLiudmila A. GorelovaZhidong ZhangLiudmila LoghinaZhifang ShiLiudmila N. StepanovaZhifei YanLiudmila RadionovaZhifeng WangLiudmila TsygankovaZhifu CaoLiudmyla StorozhukZhi-Gang GuLiuyi RenZhigang YinLiviu DutaZhigang ZangLiwei D. GengZhiguo ZhangLiwei YangZhihai HeLiwei YeZhi-Hao CuiLiyuan ShengZhihe DouLjerka Slokar BenićZhiheng HuLjiljana Damjanović-VasilićZhihua XiongLjiljana KljajevićZhila IzadiLjubica AndjelkovicZhili LiLjubisa NikolicZhiming YanLlewellyn MorseZhi-Peng WuLluis TorresZhiping ChenLluïsa EscodaZhiping XiongLoganathan MohanZhiping ZengLoganathan RangasamyZhiqian WangLohan Nicoleta MonicaZhiqiang LiangLok Lew Yan VoonZhiqing LiLok ShresthaZhitian ShiLokanath PatraZhitong BaiLoke Kok FoongZhixing GanLokendra SinghZhixiong ZengLokesh Koodlur SannegowdaZhiyong GaoLokesh SainiZhiyong LiLokman GemiZhiyuan ChenLong ChenZhiyuan LiuLong WuZhiyuan QianLong XuZhiyuan WeiLong Y. ChiangZhong YangLong ZhangZhongchen LuLongchao ZhuoZhongfa MaoLongfei LiZhonghai XuLonghai GuoZhonghao LiuLonghui ZhangZhongjing RenLonglong MaZhongmin XiaoLongtao MaZhongpeng WangLóránt Dénes DávidZhongwei HuLoredana ContrafattoZhongwei MengLorena DeleanuZhongwei ZhangLorena SegaleZhongwei ZhaoLorenza MaddalenaZhongxia ShangLorenzo AppoloniaZhou LiLorenzo BevilacquaZhuguo LiLorenzo BreschiZhujin YangLorenzo Paolo IngrassiaZhuojia FuLoreta Tamašauskaitė-TamašiūnaitėZhuoran JiangLoreto ValenzuelaZhwan Dilshad Ibrahim SktaniLoris MolentZia Ur RehmanLotfi Ben SaidZian Cheak TiuLotfi BessaisZian JiaLothar SpießZidan GongLouay S. YousufZidong WangLouis HennetŽiga GosarLouis W. Y. LiuZiga SmitLouiza DimowaZihao DingLourdes Maria Silva De SouzaZihao OuLozica IvanovicZihe GaoLu JiangZijian SongLu RenZike WangLu WangZimina MariiaLuana Da Silva Magalhães ForeziZine El Abiddine FellahLubinda WalubitaZingway PeiĽubomír ČaplovičZinovi DashevskyĽubomír MedveckýZiwen LiuLubos HesZixian JiaĽuboš KaščákZixiang WengLubos KristakZlata Kelar Tučeková TučekováLuboš SmutkaZobia NoreenLuc HébrardZoha AziziLuca CirilloZohaib KhurshidLuca Esposito (University of Milan)Zohra BenzartiLuca Esposito (University of Napoli)Zohre Mousavi NejadLuca GiachettiZoi G. LadaLuca GiorleoZoia DuriaginaLuca GironiZoltán Ádám TamusLuca LavagnaZoltán DudásLuca MaioloZoltan HomonnayLuca MenichettiZoltan KarolyLuca PezzatoZoltan PasztoryLuca QuagliatoZoltán WeltschLuca ScottiZongan LiLuca SorrentinoZongbin LiLuca SpiridigliozziZoran KarlovićLuca ValentiniZoran PrijićLuca ZaniniZoran RadakovicLucas Bragança CarvalhoZoran StevićLucas Da Fonseca Roberti GarciaZorica LopičićLucas Massaro SousaZu Zeng QinLucélia HoehneZuankai WangLucia BaldinoZuan-Tao LinLucia CarlucciZubair SyedLucia Carmen TrincăZuhair JamainLucia CavigliZulfirdaus ZakariaLucia D’accoltiZulhelmi AmirLucia FagiolariZulqarnain SajidLucia GarijoZuojia WangLucia MeleZupeng ChenLucía NasiZuxing ZhangLucia-Antoneta ChicosZuzana BirčákováLucian Gabriel ZamfirZuzana KronekovaLucian PetrescuZuzana MarcalikovaLuciana PereiraZuzana MelichováLuciana Reyes Pires KassabZuzana MurčinkováLuciano CantoneZuzana ZajíčkováLuciano Pessanha MoreiraZuzana ZvakovaLucie HympánováZuzanka TrojanovaLucie JezerskaZuzanna BielanLucien VelevaŽydrunas KavaliauskasLucilla IacuminZygmunt MiknoLúcio FrigoŽymantas Rudžionis

